# FIREVAT: finding reliable variants without artifacts in human cancer samples using etiologically relevant mutational signatures

**DOI:** 10.1186/s13073-019-0695-x

**Published:** 2019-12-17

**Authors:** Hyunbin Kim, Andy Jinseok Lee, Jongkeun Lee, Hyonho Chun, Young Seok Ju, Dongwan Hong

**Affiliations:** 1 Bioinformatics Analysis Team, National Cancer Center, 323 Ilsan-ro, Ilsandong-gu, Goyang-si, Gyeonggi-do 10408 Republic of Korea; 2 Department of Mathematics and Statistics, Boston University, Boston, MA 02215 USA; 3 Graduate School of Medical Science and Engineering, Korea Advanced Institute of Science and Technology, Daejeon, 34141 Republic of Korea

**Keywords:** Cancer genomics, Somatic mutations, Variant filtering, Mutational signatures, Sequencing artifact, Bioinformatics software, Computational oncology

## Abstract

**Background:**

Accurate identification of real somatic variants is a primary part of cancer genome studies and precision oncology. However, artifacts introduced in various steps of sequencing obfuscate confidence in variant calling. Current computational approaches to variant filtering involve intensive interrogation of Binary Alignment Map (BAM) files and require massive computing power, data storage, and manual labor. Recently, mutational signatures associated with sequencing artifacts have been extracted by the Pan-cancer Analysis of Whole Genomes (PCAWG) study. These spectrums can be used to evaluate refinement quality of a given set of somatic mutations.

**Results:**

Here we introduce a novel variant refinement software, FIREVAT (FInding REliable Variants without ArTifacts), which uses known spectrums of sequencing artifacts extracted from one of the largest publicly available catalogs of human tumor samples. FIREVAT performs a quick and efficient variant refinement that accurately removes artifacts and greatly improves the precision and specificity of somatic calls. We validated FIREVAT refinement performance using orthogonal sequencing datasets totaling 384 tumor samples with respect to ground truth. Our novel method achieved the highest level of performance compared to existing filtering approaches. Application of FIREVAT on additional 308 The Cancer Genome Atlas (TCGA) samples demonstrated that FIREVAT refinement leads to identification of more biologically and clinically relevant mutational signatures as well as enrichment of sequence contexts associated with experimental errors. FIREVAT only requires a Variant Call Format file (VCF) and generates a comprehensive report of the variant refinement processes and outcomes for the user.

**Conclusions:**

In summary, FIREVAT facilitates a novel refinement strategy using mutational signatures to distinguish artifactual point mutations called in human cancer samples. We anticipate that FIREVAT results will further contribute to precision oncology efforts that rely on accurate identification of variants, especially in the context of analyzing mutational signatures that bear prognostic and therapeutic significance. FIREVAT is freely available at https://github.com/cgab-ncc/FIREVAT

## Background

High-throughput sequencing technology has led to an explosion in the sheer volume of genomic data in the past decade. Because this technology produces genome-wide somatic mutation profiles within a reasonable timeframe and at a reasonable cost [1], many research groups, particularly International Cancer Genome Consortium (ICGC) and The Cancer Genome Atlas (TCGA), have produced population-scale whole-exome sequencing (WES) and whole-genome sequencing (WGS) datasets from most common cancer types. As a consequence, at least 100,000 tumor samples have been sequenced and computationally analyzed to date [2–8]. The vast majority of the sample-specific mutation calls, usually in Variant Call Format (VCF), are publicly available through online databases [9–11], which are invaluable sources of future genome studies and precision oncology. Simultaneously, tumor samples continue to be sequenced in hospitals to inform clinical decisions.

However, as sequencing data are produced and analyzed by heterogeneous groups, marked differences in the precision and sensitivity of the mutation calls become apparent. Due to variations in cost, capacity, and approach to bioinformatic analysis, provisional mutation calls include numerous false positives and negatives [12]. Sometimes, mutation calls are contaminated by experimental artifacts that accumulate during tissue handling and sequencing procedures, such as preparation of tissues into formalin-fixed paraffin-embedded (FFPE) samples, 8-oxoG-mediated errors, chimeric reads, and others [13–15]. Artifactual variants have been shown to be a major culprit of clinical misinterpretations. In a routine genotyping of *KRAS* mutations in metastatic colorectal cancer patients, 53 (4.7%) out of 1130 FFPE samples had *KRAS* mutations that were validated as artifacts due to DNA fragmentation [16]. In another study validating the detection of the T790M mutation in the epidermal growth factor receptor (*EGFR)* gene in non-small cell lung cancer (NSCLC) patients, up to 48.5% of T790M mutations were FFPE-related artifacts [17]. Moreover, in a clinical trial that investigated the efficacy of the tyrosine kinase inhibitor (TKI) erlotinib in NSCLC patients [18], previously unidentified *EGFR* mutations were reported. The study concluded a lack of survival benefits in using erlotinib for patients with *EGFR* mutations and no clinical evidence to distinguish *EGFR*-mutant and wild-type patients for administration of the TKI. However, these novel mutations were later shown to be artifacts arising from the paraffin fixation process [19]. Therefore, false positive calls should be systematically eliminated for accurate downstream genome analysis at a population scale.

To eliminate false positives, somatic variant refinement is usually performed and often involves manual inspection of binary alignment map (BAM) files or experimental validation. However, the manual nature of this task may hamper reproducibility and scalability. To address this issue, a standard operating procedure for manual review has been developed [20], but the process remains labor-intensive and time-consuming. While machine learning- and deep learning-based approaches to variant calling and refinement have been published [21, 22], results generated from these black-box models lack human interpretability, which hinders their widespread usability. In addition, these intensive bioinformatic analyses often require re-exploration of raw datasets (i.e., BAM files), necessitating massive computing power and data storage. Sometimes, the acquisition of original BAM files for publicly available mutation calls is technically intricate due to the large file size. Collectively, flexible and comprehensive algorithms that allow quick mutation screening and efficient variant refinement are imperative for conducting downstream analyses at a population scale using VCF files.

Recently, the Pan-cancer Analysis of Whole Genomes (PCAWG) consortium generated 65 single-base substitution (SBS) mutational signatures from over 4600 whole cancer genomes and 19,000 cancer exomes [23]; these signatures have been incorporated as version 3 into the v89 release of Catalog of Somatic Mutations in Cancer (COSMIC) [24]. Each of the signatures exhibits an expected spectrum of mutations by certain mutational processes. For example, one of the signatures, termed SBS7, features preferential C>T mutations [25], whereas SBS4, a mutational spectrum of tobacco smoke exposure, is characterized by C>A mutations with a strong transcriptional strand bias [26]. Interestingly, a subset of these signatures is thought to be artifact-mediated calls. In fact, the single nucleotide substitutions observed in these artifactual signatures were shown to be enriched in false positive variant calls by a previous investigation on the reliability of WES in breast cancer samples [27]. Signatures that correspond to the enriched contexts are also reportedly correlated with germline variant contamination and DNA damage during experimental processes [23]. These findings suggest the feasibility of using mutational signatures to perform variant refinement.

Here we present FInding REliable Variants without ArTifacts (FIREVAT), an open source software toolkit that eliminates sequencing artifacts from biologically and clinically relevant point mutations in human cancer samples. Our toolkit automatically decomposes the spectrum of mutation calls in user-supplied VCF files into 65 known mutational signatures, and filters variant calls that better fit error signatures. FIREVAT outputs a Hypertext Markup Language (HTML) report for each sample that undergoes variant refinement along with VCF files of refined and artifactual mutations, which can be used for downstream analysis. FIREVAT is implemented as an R package and can run on computers with limited resources such as a laptop. We validate the performance of FIREVAT by carrying out various benchmark experiments on three publicly available mutation callsets comprising 678 tumor-normal pairs obtained from multi-center validated sequencing, multiple cancer types, and multi-region WES. Along with variant refinement optimized for each sample, our novel evaluation method implemented in FIREVAT can be used as a proxy for quality control of other post variant calling efforts.

## Implementation

### Overview of FIREVAT

**Fig. 1:**
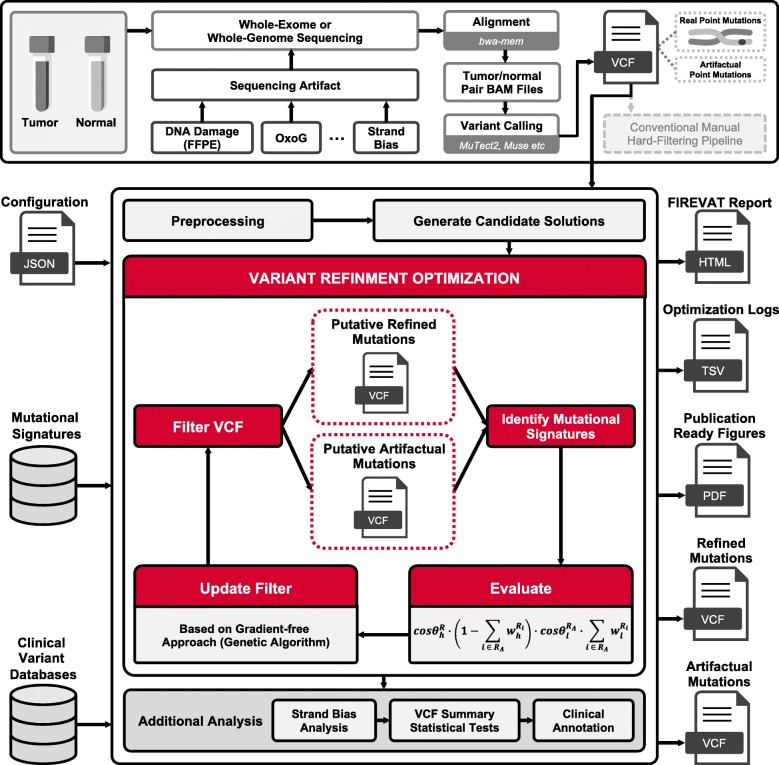
Overview of the FIREVAT workflow. The top panel represents the conventional workflow for processing sequencing data of the tumor and matched normal genomes. Sequencing artifacts are introduced in various steps of the experimental process. After calling variants with software such as MuTect2 and Muse, manual filtering is performed to mitigate false positives. FIREVAT automates this variant refinement task by leveraging the COSMIC mutational signatures (version 3). The primary input parameters for FIREVAT include a VCF file and a configuration JSON file, which specifies instructions on how to compute the desired filter parameters. In the preprocessing step, FIREVAT derives these parameters for each point mutation. A set of candidate solutions is generated to help the optimization process converge faster. Next, FIREVAT searches for a set of parameters that maximizes the objective function (“Evaluate”) using GA, a gradient-free approach. At each iteration, the original set of mutations is divided into refined and artifactual groups, followed by a mutational signature analysis of each group. FIREVAT further analyzes the refinement outcomes by assessing strand bias and significant differences between the refined and artifactual variant groups. Each variant is also annotated using clinical databases. FIREVAT generates output files for the following items: HTML report, optimization logs, and VCFs for refined, and artifactual variants

Unrefined mutations can lead to inaccurate mutational signature analysis inundated with artifactual signatures that obscure the identification of etiologically relevant mutational patterns (Additional file 1: Note S1, Additional file 2: Figures S1–S4). FIREVAT addresses this problem by performing variant refinement guided by mutational signatures known to be representative of sequencing artifacts. By iteratively evaluating weights attributed to sequencing artifact signatures, FIREVAT determines optimal filtering cutoff values that effectively separate artifactual variants from real variants, thereby isolating biologically and clinically relevant mutagenesis signatures (Fig. 1).

To sequester somatic point mutations that exhibit latent artifactual characteristics, FIREVAT employs mutational signatures extracted by the PCAWG consortium [23]. In particular, 18 signatures associated with sequencing artifacts are exploited: SBS27, SBS43, SBS45, SBS46, SBS47, SBS48, SBS49, SBS50, SBS51, SBS52, SBS53, SBS54, SBS55, SBS56, SBS57, SBS58, SBS59, and SBS60 (Additional file 2: Figures S5 and S6). FIREVAT utilizes a VCF file as the primary input, preferably called from a tumor and matched normal pair, and a JavaScript Object Notation (JSON) configuration file that specifies how each filter parameter should be derived and treated (Additional file 1: Note S2). Here we define a filter as a set of numerical values that corresponds to the various quality metrics (e.g., average reference allele base quality in the tumor sample) either reported by the caller or computed by the user for each variant.

Next, somatic point mutations are selected and each desired filter parameter is obtained for each mutation. A preliminary mutational signature analysis is conducted to determine if variant refinement is necessary based on the weights of the artifact signatures. Each filter parameter is independently traversed while dividing the set of given mutations into putative refined and artifactual mutations. This process results in a set of candidate solutions that is later used to expedite convergence in the FIREVAT variant refinement optimization stage.

The FIREVAT objective function aims to maximize cosine similarity score of the resulting signature analysis, minimize the summed weights of the artifact signatures in the set of refined mutations, and thereby enrich the contribution of artifact signatures in the low-quality artifactual mutations. Hence, maximization of the FIREVAT objective function is an abstraction of the multiobjective optimization problem that yields Pareto optimal values of the four aforementioned criteria [28]. Various information supporting each variant, such as quality scores and read depths computed by variant calling software, constitutes the FIREVAT algorithm search space, which is explored using a genetic algorithm (GA), a gradient-free approach.

After the most optimized parameters are determined, FIREVAT performs strand bias analysis, analyzes the statistical significance of each parameter, and annotates each variant using clinical variant databases such as ClinVar [29] and COSMIC [24]. Filtered mutations in clinically actionable genes can be rescued at the final stage. FIREVAT outputs VCF files of high-quality refined variants and low-quality artifactual variants and provides a thorough report of the refinement processes and results. These results are presented to the user in the form of an HTML file, which includes intuitive publication-ready figures and tables. FIREVAT also supports multiprocessing on each VCF file and enables a scalable computation of multiple samples on demand.

### Inputs

FIREVAT utilizes a VCF file as the primary input as well as a JSON configuration file detailing instructions on how to extract desired filter parameters. FIREVAT uses the bedr R software package [30] to read the input VCF file. The genomic assembly of the VCF file is processed using the BSgenome R software package. The FIREVAT R software package already includes default configuration files for some of the widely used variant callers. Users are also able to generate custom configuration files on demand. Another important input is the reference mutational signature matrix. The COSMIC mutational signature version 3 matrix is included in the package as FIREVAT requires this a priori information to operate its variant refinement function. Alternatively, users can supply their own matrix provided that signatures with artifactual characteristics are included.

### Preprocessing

FIREVAT first selects point mutations in the user-supplied VCF file. To execute mutational signature analysis, FIREVAT prematurely terminates if the number of point mutations does not satisfy the minimum requirement of 50 point mutations. A preliminary signature analysis is performed to assess whether refinement is necessary. FIREVAT deems a given set of mutations refined if the initial sum of artifact signature weights, obtained from unrefined mutations, is lower than the minimum threshold (default = 0.05). Next, each of the desired filter parameters in the input configuration JSON file is computed for each point mutation.

### Candidate solution generation

To create benchmark objective values that help the GA optimization converge faster, FIREVAT generates a set of candidate solutions. Each candidate solution is a vector of cutoff values for quality-related attributes for the variants called (e.g., variant allele fraction ≥ 5% and tumor reference allele read depth ≥ 10). Each filter parameter is then traversed from the minimum to the maximum value observed in the input VCF file. At each increment, FIREVAT divides the set of point mutations into refined and artifactual groups depending on each filtering criterion. Subsequently, signature analysis is conducted for both groups to derive the objective value (see the “Variant refinement optimization” section below). Parameter values that yield nonzero objective values are passed to the GA optimization stage as potential solutions.

### Variant refinement optimization

#### Variant filtering

Given the original unrefined set of point mutations *M*, we define the subset of mutations *M*_*h*_ as the set of variants that satisfy all filter parameters *f*_1_, *f*_2_, *f*_3_,..., *f*_*k*_, collectively referred to as *F* henceforth. The remaining mutations are defined as *M*_*l*_. Therefore, *M = M*_*h*_ + *M*_*l*_, where:

$$ {\displaystyle \begin{array}{l}{M}_h={f}_1\wedge {f}_2\wedge {f}_3\cdots \wedge {f}_k\\ {}{M}_l=\neg \left({f}_1\wedge {f}_2\wedge {f}_3\cdots \wedge {f}_k\right)\end{array}} $$

#### Reference mutational signatures

We define the reference mutational signatures as follows:

$$ R=\left[\begin{array}{lll}{r}_1^1& \cdots & {r}_{65}^1\\ {}\vdots & \ddots & \vdots \\ {}{r}_1^{96}& \cdots & {R}_{65}^{96}\end{array}\right] $$

Note that ${r}_i^j$ represents the probability of the mechanism *i* (e.g., ultraviolet radiation) to cause a mutation type of *j* (e.g., cytosine to thymine transition at dipyrimidines) in the COSMIC mutational signatures (65 signatures in version 3). Let *R*_*A*_ be the matrix of signatures related to sequencing artifacts (18 signatures, Additional file 2: Figure S5).

#### Mutational signature identification

Given *M*_*x*_ and *R*, the identification of underlying mutational signatures can be written as:

$$ S\left({M}_x,R\right)\to \mathit{\cos}{\theta}_x^R, sig{s}_x^R,{w}_x^R $$

The results of signature identification *S* include the cosine similarity score $\mathit{\cos}{\theta}_x^R$, vector of identified mutational signatures $sig{s}_x^R$, and vector of weights for each identified mutational signature ${w}_x^R$. Therefore, the identification of mutational signatures in the set of refined mutations and artifact mutations can be expressed as follows:

$$ {\displaystyle \begin{array}{l}S\kern0.28em \left({M}_h,R\kern0.28em \right)\to \cos {\theta}_h^R, sig{s}_h^R,{w}_h^R\\ {}S\kern0.28em \left({M}_l,R\kern0.28em \right)\to \cos {\theta}_l^R, sig{s}_l^R,{w}_l^R\end{array}} $$

In particular, FIREVAT computes the summed weights of sequencing artifact-related signatures, denoted as:

$$ \sum \limits_{i\in A}{w}_x^{R_i} $$

Our algorithm uses deconstructSigs [31] to construct the trinucleotide spectrum matrix, MutationalPatterns [32] to derive the objective value, and Mutalisk [33] to narrow down biologically feasible signatures.

#### Objective function

FIREVAT explores various filter parameters *F* to find the most optimized filtering parameter cutoffs to maximize the following objective value:

$$ {\displaystyle \begin{array}{l}\operatorname{maximize}\kern0.28em \cos {\theta}_h^R.\left(1-\sum \limits_{i\in A}{w}_h^{R_i}\right).\cos {\theta}_l^{R_A}.\sum \limits_{i\in A}{w}_l^{R_i}\\ {} subject\kern0.34em to\\ {}\cos {\theta}_h^R\in S\left({M}_h,R\kern0.28em \right),\\ {}{w}_h^R\in S\left({M}_h,R\kern0.28em \right),\\ {}\cos {\theta}_l^{R_A}\in S\kern0.28em \left({M}_l,{R}_A\kern0.28em \right),\\ {}{w}_l^R\in S\left({M}_l,R\kern0.28em \right)\end{array}} $$

To compute the most optimized objective value, FIREVAT uses the GA R package [34], which is an implementation of the genetic algorithm. Each “gene” in the initial GA population constitutes a vector of arbitrary filter parameters. In subsequent generations, the GA selects members of the population that have higher objective values. Shown below is the pseudocode for the FIREVAT variant refinement optimization algorithm.

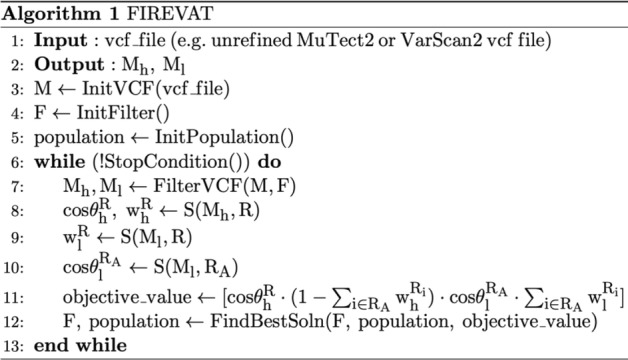



The objective function is both an abstraction and a mathematical estimation of refinement outcomes using mutational signatures. We created and tested 10 different objective functions that vary in their combination and weights of the four variables from signature analysis results (Additional file 1: Method S1). We used 28 MC3 samples to benchmark the performance of the objective functions as well as FIREVAT input parameters (Additional file 2: Figure S7).

### Additional analysis

#### Strand bias analysis

Strand bias found in a putative variant is known to be indicative of sequencing artifacts [35]. To account for this error, FIREVAT uses forward and reverse read counts of reference and alternate alleles to perform strand bias analysis using Fisher’s exact test and corrects for multiple testing.

#### Filter parameter statistical significance test

FIREVAT applies the conjunction of various filter parameters, specified by the user, to derive refined mutations. For this reason, the resulting distribution of the artifact mutations does not always start or end at the hard-filtering value. After the variant refinement is complete, FIREVAT tests whether the distributions of values for each parameter are significantly different (Mann Whitney) among the original, refined, and artifactual sets of mutations. This information can be used to assess which specific filter significantly affected the refinement outcome in the HTML report.

#### Variant annotation

Each variant is annotated using the user-supplied clinical variant database. Variants classified as artifacts but bearing clinical significance of any study-specific reasons can be salvaged. For all of our analyses, we annotated pathogenic variants in ClinVar (20190211 version) [29].

### Outputs

The standard FIREVAT output includes a report of the refinement processes and outcomes as an HTML file, vector graphic files of all figures generated in the HTML report, a refinement optimization log file, a RData file of all FIREVAT generated data for downstream analyses, a VCF file comprised of refined mutations, and a VCF file comprised of artifactual mutations. All resulting figures are generated using the ggplot2 [36] and ggpubr R software packages.

### Validation data and processing

For the evaluation of FIREVAT variant refinement performance, we used three datasets: the MC3 dataset [37], TCGA Genomic Data Commons (GDC) dataset, and multi-region WES of breast cancer dataset [27] (Additional file 1: Note S3). For the evaluation of post FIREVAT signature analysis, we used the following TCGA datasets (Additional file 3: Table S1): head and neck squamous cell carcinoma (HNSC) [38], breast invasive carcinoma (BRCA) [39], pancreatic adenocarcinoma (PAAD) [40], and stomach adenocarcinoma (STAD) [41]. For the characterization of artifactual signatures, we used the TCGA-HNSC, TCGA-BRCA, TCGA-PAAD, and TCGA-STAD datasets as well as five additional TCGA datasets: glioblastoma multiforme (GBM) [42], kidney renal clear cell carcinoma (KIRC) [43], acute myeloid leukemia (LAML) [44], lung adenocarcinoma (LUAD) [45], and liver hepatocellular carcinoma (LIHC) [46]. We used R version 3.5.1 to run FIREVAT v0.4.2 on these datasets (Additional file 1: Method S2 and Method S3). We also downloaded DToxoG (v1.14.4.1) from https://seqwaremaven.oicr.on.ca/artifactory/seqware-dependencies/org/broadinstitute/DToxoG/1.14.4.1/ and used it for benchmarking purposes [13].

### Performance validation (multi-center mutation calling in multiple cancers (MC3)) dataset

We downloaded the Multi-Center Mutation Calling in Multiple Cancers (MC3) dataset [37] from the National Cancer Institute (NCI) GDC data portal [3]. To define ground truth data, we used the “mc3.v0.2.9.CONTROLLED_lt3_b.maf” file. At the outset, we retained point mutations in this Mutation Annotation Format file. Next, we selected samples that had WES, WGS, and RNA-seq mutation validation status information. We further selected samples that had only one matching normal sample. To establish ground truth, we herein describe how we determined real somatic and artifactual mutations in the MC3 dataset. To identify real somatic mutations, we first selected variants that were captured in the targeted exonic region. We further selected variants that were validated and statistically powered in either WGS or targeted sequencing in terms of read evidence according to the MC3 definition (“mutval_targeted_status” = “validated_powered” or “mutval_wgs_status” = “validated_powered”). Among these variants, we finally selected variants that were also validated and statistically powered in WES (“mutval_wex_status” = “validated_powered”). To identify artifactual variants, we first selected variants that did not satisfy the requirements for classification of real somatic mutations. Then, we screened for variants that were unvalidated and statistically unpowered in WGS (“mutval_wgs_status” = “unvalidated_unpowered”), labeling these as artifactual mutations. We used 774 VCF files from 360 samples that had more than 500 real somatic or artifactual mutations as part of our variant refinement performance validation study (Additional file 1: Note S3). We also selected 28 samples that had less than 500 real somatic or artifactual mutations and used these to benchmark the performance of various objective functions and FIREVAT input parameters.

The MC3 dataset includes a number of different callsets for each sample. We used the MuTect, Muse, SomaticSniper, and Varscan hg19 callsets in our FIREVAT refinement validation study. Of these, we excluded the SomaticSniper callset because our preliminary analysis on several SomaticSniper VCF files yielded initial sum of artifact signature weights lower than the minimum threshold, indicating that mutational signature-based FIREVAT refinement may not be necessary. For the remaining MuTect, Muse, and Varscan callsets, we ran FIREVAT v0.4.2 using their respective configuration files (Additional file 3: Table S2). We evaluated FIREVAT variant refinement performance using the ground truth data. To compare FIREVAT refinement performance, we applied three other manual hard-filtering approaches on the same MC3 validation samples. These approaches were suggested by variant caller developing groups: Lancet filter (LAN-F) [47], MuTect filter (MUT-F) [48], and Varscan filter (VAR-F) [49] (Additional file 1: Method S2). Subsequently, for each filtering method, we analyzed the performance evaluation metrics (precision, sensitivity, specificity, F1 score, and accuracy) against the sum of signature artifact weights. We also used the PCAWG Platinum mutational signatures for benchmarking purposes [50].

### Consistency validation (multi-region whole-exome sequencing data of breast cancer) dataset

We downloaded the FASTQ files of the 24 breast cancer WES pairs (technical and biological replicates) [27] from the Sequence Read Archive (SRA) with accession number SRP070662. The raw sequences were aligned to hg19 using bwa-mem [51]. The bam files were sorted and assigned into read groups using SAMtools [52]. The PCR duplicates were marked with Picard (http://broadinstitute.github.io/picard/). Realignment and base recalibration were performed using GATK [53] with 1000G_phase1.indels.hg19.vcf and Mills_and_1000G_gold_standard.indels.hg19.vcf as known targets. We also used dbsnp_b141.vcf for the base recalibration. Variant calling was performed using MuTect2 [48] for tumor/normal paired calling with default parameters. Only the PASS (high confidence somatic mutations) calls were used for the subsequent downstream validation analyses.

To evaluate the FIREVAT variant refinement performance on the multi-region WES breast cancer samples, we downloaded the supplementary tables from the original manuscript for technical and biological replicates. For the benchmark study between DToxoG, a variant was considered real if “Ampliseq Call” was somatic and artifactual otherwise. Our MuTect2 point mutations present in the tables were used for evaluation. To generate the UpSet and Venn diagrams pertaining to this dataset, we used the UpSetR [54] R package and the python matplotlib [55] library.

### Signature analysis (TCGA-GDC) dataset

The MuTect2 hg38 VCF files were downloaded from the GDC. The TCGA drug response data were downloaded using the R package TCGAbiolinks [56]. The clinical data (version 2016-04-27) for TCGA-HNSC and TCGA-LUAD were downloaded from Xena Browser [11].

For the mutational signature analysis of TCGA-HNSC and TCGA-LUAD samples, we ensured that Mutalisk considered the tobacco smoking signatures SBS4 and SBS29 in the unrefined, refined, and artifactual mutation sets. For the mutational signature identification of samples with platinum therapy response data (TCGA-BRCA, TCGA-PAAD, and TCGA-STAD) [57–59], we ensured that Mutalisk considered the HR-deficiency signature SBS3 in the unrefined, refined, and artifactual mutation sets while keeping all other FIREVAT parameters default. For other TCGA cohorts, we used the default options of FIREVAT to determine the most likely signatures. To analyze the enrichment of sequence contexts, we used the ggseqlogo [60] R package.

### HCC1954

We obtained the variants list (VCF file) called from the whole-genome sequencing of HCC1954 from the ICGC data portal.

### FFPE dataset

We obtained the formalin-fixed paraffin-embedded (FFPE) dataset to characterize FFPE-specific variants from the Sequence Read Archive with the accession numbers PRJNA301548 and SRP065941 [61]. Reads were aligned with bwa-mem to hg19 and variants were called with MuTect2 with the default settings.

### ICGC-TCGA-DREAM somatic mutation calling challenge dataset

We downloaded the synthetic variant data from the DREAM Challenge [62]. Using the set 1 from the Challenge, we called somatic mutations using MuTect, Muse, and Varscan with the default settings and additionally using MuTect with a panel of normal and the TLOD option to compare against submitted refinement methods. Among the methods submitted to the challenge, pipelines that used custom alignment and BAM file generation or callers other than MuTect, Muse, or Varscan were excluded from our comparative analysis in order to objectively evaluate the post hoc filtering performance of FIREVAT. We also compared DToxoG [13] results for all of the callsets that had read count information for each strand.

### Mutational signature matrices

We used the COSMIC mutational signatures version 3 [23] for primary analyses and used the PCAWG Platinum mutational signatures [50] to validate presence of artifactual signatures.

## Results

### Evaluation of FIREVAT variant refinement performance on real-world datasets

We evaluated the validity and reliability of FIREVAT variant refinement using two publicly available real-world datasets comprising 384 total samples (Additional file 1: Note S3).

The first dataset was the MC3 dataset [37], which consists of mutation calls in VCF file format from multiple callers for over 11,000 tumor samples (Additional file 3: Table S3). Because confidence in the detection of somatic mutations can be ascertained by additional sequencing methods (e.g., WES, WGS, and/or RNA-seq), we can determine whether a variant is a clonal true positive. We used the MuTect, Muse, and Varscan MC3 callsets comprising 774 VCF files from 360 samples. The MuTect callset included 191,118 (93.86%) clonal true positives (real somatic mutations) and 12,511 (6.14%) false positives (artifactual mutations). In the Muse callset, 207,784 (82.17%) real somatic mutations and 45,091 (17.83%) artifactual mutations were present. The Varscan callset had the highest number and proportion of artifactual mutations, including 117,803 (60.73%) real somatic mutations and 76,179 (39.27%) artifactual mutations. In total, 516,705 (79.43%) real somatic mutations and 133,781 (20.57%) artifactual mutations were utilized. We used five metrics to evaluate FIREVAT performance: precision, sensitivity, F1 score (harmonic mean of the precision and sensitivity), specificity, and accuracy (Additional file 1: Method S2). We compared the variant refinement performance of FIREVAT on the MC3 samples against three other filtering methods (LAN-F, MUT-F, and VAR-F; Additional file 1: Method S2). Each filtering approach was independently applied on each of the three MC3 callsets.

**Fig. 2:**
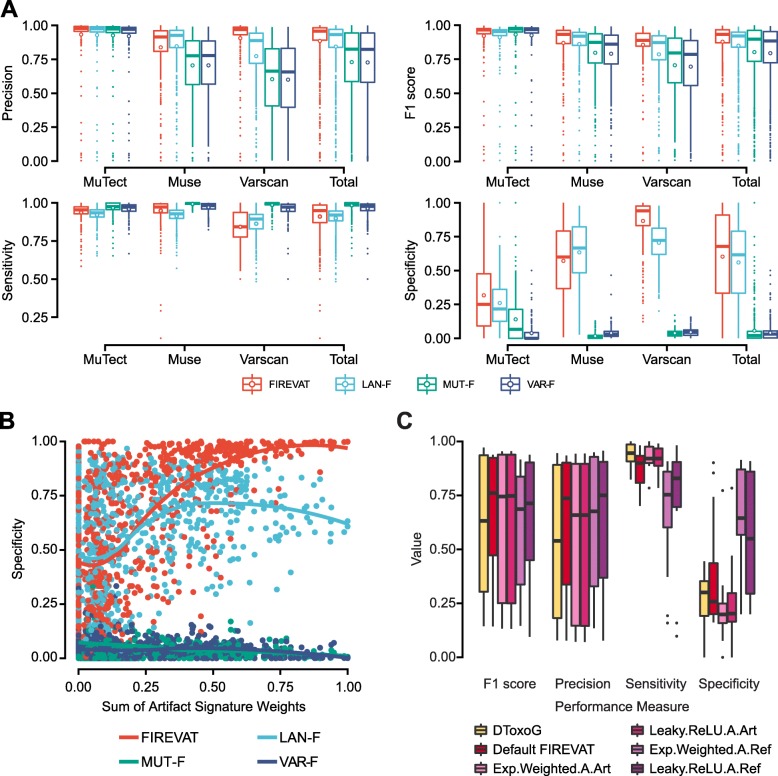
Evaluation of FIREVAT variant refinement performance on real-world datasets against ground truth. **a** FIREVAT variant refinement performance on 360 MC3 samples with known ground truth data against three other manual hard-filtering approaches: Lancet (light blue: LAN-F), MuTect (green: MUT-F), and Varscan (navy: VAR-F). FIREVAT refinement results yielded the highest F1 score when evaluated on combined callsets. **b** Scatterplot of the specificities and the initial sum of artifact signature weights for FIREVAT and the other filtering approaches. FIREVAT refinement specificity showed a positive correlation with the initial sum of artifact signature weights. **c** Variant refinement was performed using FIREVAT and DToxoG on the WES data of 6 breast cancer samples with technical replicates (*n* = 12). We used five different objective functions that assign different weights to each of the four terms constituting the objective value. When evaluated against the ground truth data, FIREVAT achieved the highest precision level and F1 score against DToxoG

FIREVAT performed at the highest level for four of the five metrics compared with the three widely adopted manual filtering approaches when evaluated on all mutations called by MuTect, Muse, and Varscan (Fig. 2a): precision (median = 0.958), F1 score (median = 0.933), specificity (median = 0.678), and accuracy (median = 0.908) (Additional file 3: Tables S4 and S5).

We additionally compared the FIREVAT performance in each callset. Using the mutations called by MuTect, FIREVAT yielded the highest level of precision and specificity with median values of 0.978 and 0.250, respectively, while achieving a sensitivity comparable to those of the other filtering approaches. While the MUT-F and VAR-F methods yielded higher median F1 scores (MUT-F = 0.972, VAR-F = 0.966) than FIREVAT (median = 0.964), these methods performed poorly in terms of specificity (MUT-F = 0.067, VAR-F = 0.000).

In the Muse callset, FIREVAT yielded the highest F1 score with a median of 0.932. Similar to the performance observed in the MuTect callset, the median sensitivities of the MUT-F (0.996) and VAR-F (0.982) methods were higher than that of FIREVAT (0.973), but the median specificities were lower (MUT-F = 0.010, VAR-F = 0.031, FIREVAT = 0.601). The LAN-F method resulted in the highest specificity (0.667) in the Muse callset but had a lower sensitivity (0.927) than FIREVAT (0.973).

The FIREVAT refinement results of the MC3 Varscan callset yielded the highest specificity with a median of 0.941, filtering out 88.9% of the original variants on average. In contrast, 23.4% and 10.8% mutations were filtered out in the Muse and MuTect callsets. The MUT-F and VAR-F methods achieved higher median sensitivities (MUT-F = 0.996, VAR-F = 0.972) than FIREVAT (0.842). However, the FIREVAT median specificity was dramatically higher than those of the other methods (MUT-F = 0.034, VAR-F = 0.043, FIREVAT = 0.941).

The proportion of filtered variants was positively correlated to the filtering stringency identified by FIREVAT on each callset. For example, the median cutoff for the minimum number of altered reads in the tumor sample was 3 for the MuTect callset and 8 for the Varscan callset (Additional file 2: Figures S8–S10). The need for stricter filtering parameters determined by FIREVAT was consistent with a previously published benchmark study that reported that Varscan variant caller has the highest false positive rate among the widely used variant callers [63]. Next, we assessed the characteristics of samples that resulted in low refinement performance. We observed that the specificity was positively correlated (Pearson correlation *r* = 0.62) with the initial sum of sequencing artifact weights (Fig. 2b) when considering all mutations from all three callers (Additional file 2: Figures S11–S17).

The second dataset utilized for evaluation of FIREVAT variant refinement performance was the multi-region WES dataset, where breast cancer samples were sequenced multiple times by biological and technical replicates. To evaluate whether variant refinement can successfully shortlist the true positive mutations validated by targeted sequencing [27], we benchmarked FIREVAT with DToxoG [13]. We compared our objective functions to DToxoG for benchmarking purposes (Fig. 2c). We found that FIREVAT consistently yielded the highest median precision level (0.736 for Default.Obj.Fn) compared to DToxoG (precision = 0.533) for the different objective functions used (Additional file 3: Table S6). FIREVAT refinement of the 6 pairs of technical replicates resulted in increased proportion of validated variants among the total number of variants (Additional file 2: Figure S18). We also applied FIREVAT to the 18 biological replicate data from the 6 breast cancer cases, for which the presence of intratumoral heterogeneity was also validated with targeted sequencing (Additional file 2: Figure S19, Additional file 3: Table S7) [27]. The increase in precision resulting from FIREVAT refinement was also observed in the ICGC-TCGA-DREAM Somatic Mutation Calling Challenge dataset [62] and in additional benchmarking studies. We also found that refinement outcomes are more accurate when FIREVAT is applied with built-in filters in variant calling software (Additional file 1: Note S4).

### FIREVAT leads to enrichment of biologically relevant signatures

**Fig. 3:**
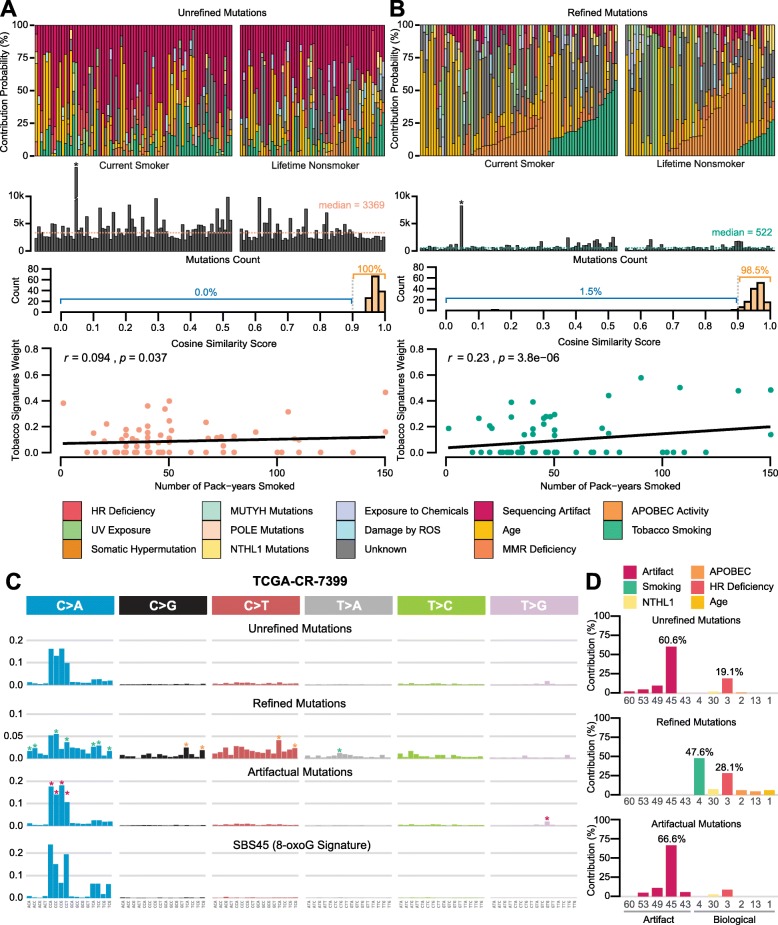
Marked improvement in mutational signature analysis explicability in the TCGA-HNSC samples using FIREVAT. **a**, **b** Each panel is comprised of the following plots from top to bottom: distribution of signature weights for the TCGA-HNSC samples (*n* = 130), bar plot of the number of mutations in each sample, histogram of cosine similarity scores from signature analysis, and correlation between the sum of tobacco signature weights and the number of pack-years among current smokers. In the two plots of signature weights, the green bars indicate the contribution weights of smoking-related signatures in each sample while the dark red bars represent that of artifactual signatures. **a** Mutational signature analysis without variant refinement. Of the 130 TCGA-HNSC samples, substantially high levels of artifactual signature weights were identified (median weight sum = 45.3%, min = 3.2%, max = 100%). The Pearson correlation between the sum of tobacco signatures and the number of pack-years was negligent using an unrefined variant list (*r* = 0.094). In particular, one sample had somatic hypermutations (15.6 mutations/Mb; denoted with an asterisk). **b** Mutational signature analysis with variant refinement by FIREVAT. Compared to the unrefined callset, the correlation between the sum of tobacco signature weights and the number of pack-years was higher (*r* = 0.23) and the weights of artifactual signatures were decreased (median weight sum = 0%, min = 0.0%, max = 30.6%). **c**, **d** Unveiling biologically relevant mutational signatures by removing mutations of artifactual signatures. **c** Mutation frequency spectrum of unrefined, refined, and artifactual mutations from the case TCGA-CR-7399 (HNSC) and SBS45 (8-oxoG signature). In the spectrum plot of refined and artifactual mutations, the asterisks represent frequency peaks found in different signatures (green = SBS4, orange = SBS2 and SBS13, red = SBS43, SBS45, SBS49, and SBS53). **d** Mutational signature weights of unrefined, refined, and artifactual mutations from TCGA-CR-7399. The tobacco smoking and APOBEC-related signatures were identified only from the signature analysis results of FIREVAT-refined mutations

To further investigate the FIREVAT refinement performance, we applied FIREVAT to public calls from 130 TCGA-HNSC (head and neck cancer) VCF files from the NCI GDC data portal [3] (Fig. 3, Additional file 2: Figure S20, Additional file 3: Table S8). We decomposed the mutational spectrums using the 65 COSMIC mutational signatures (version 3). All samples had a cosine similarity score of 0.9 or higher in the decomposition of mutational signatures. However, a substantial fraction of mutations was attributed to sequencing error signatures such as SBS45 (median weight = 28.4%), SBS43 (median weight = 14.9%), and SBS50 (median weight = 14.7%) (Fig. 3a). We refined the raw callsets using FIREVAT. FIREVAT completed the mutation refinement process in approximately 475 min (with 208 central processing units (CPUs), a GA maximum iteration = 100, and a GA population size = 200), filtering out 81.5% of the mutation calls on average (min = 49.3%, max = 98.8%, Additional file 3: Table S8). Overall, the refined callsets led to a substantially higher fraction of biologically relevant mutational signatures. The linear correlation between the amount of lifetime tobacco smoking (in pack-years at the time of diagnosis) and the mutational burden of tobacco smoking-related signatures (SBS4 and SBS29) increased (from 0.094 to 0.230) after the FIREVAT refinement process (Fig. 3b, Additional file 2: Figure S20D). In the case of TCGA-CR-7399, 5084 (88.59%) out of 5739 somatic mutation calls were labeled as artifacts by FIREVAT (Fig. 3c, Fig. 3d, Additional file 4). The C>A peaks that were apparent in the unrefined mutations of this sample exhibited a high weight of SBS45 (60.6%), the spectrum of which is known to be associated with 8-oxoG artifacts [13]. The 8-oxoG contamination was mostly attenuated (from 60.6 to 0.0%; Fig. 3c) after FIREVAT refinement and other biologically relevant mutational signatures reciprocally emerged, for example the tobacco smoking signature SBS4 (from 0.0 to 47.6%) and the APOBEC-mediated signatures SBS2 (from 1.1 to 6.1%) and SBS13 (from 0.0 to 4.6%). Of note, this head and neck cancer patient had smoked 135 pack-years at the time of diagnosis.

Moreover, we applied the FIREVAT refinement process to mutation calls from 79 TCGA samples with platinum therapy response data: 10 BRCA samples, 10 PAAD samples, and 59 STAD samples (Additional file 2: Figure S21, Additional file 3: Table S9). In one STAD WGS sample (TCGA-FP-8211), the homologous recombination (HR) deficiency signature (SBS3) emerged only in the FIREVAT-refined callset and was masked by an artifact signature (SBS60) in the original unrefined callset (Additional file 2: Figure S22). As previously suggested, the HR-deficiency signature mutational signature is a predictive marker of platinum therapy response [57]. Intriguingly, the patient showed a complete response to platinum therapy (oxaliplatin).

**Fig. 4:**
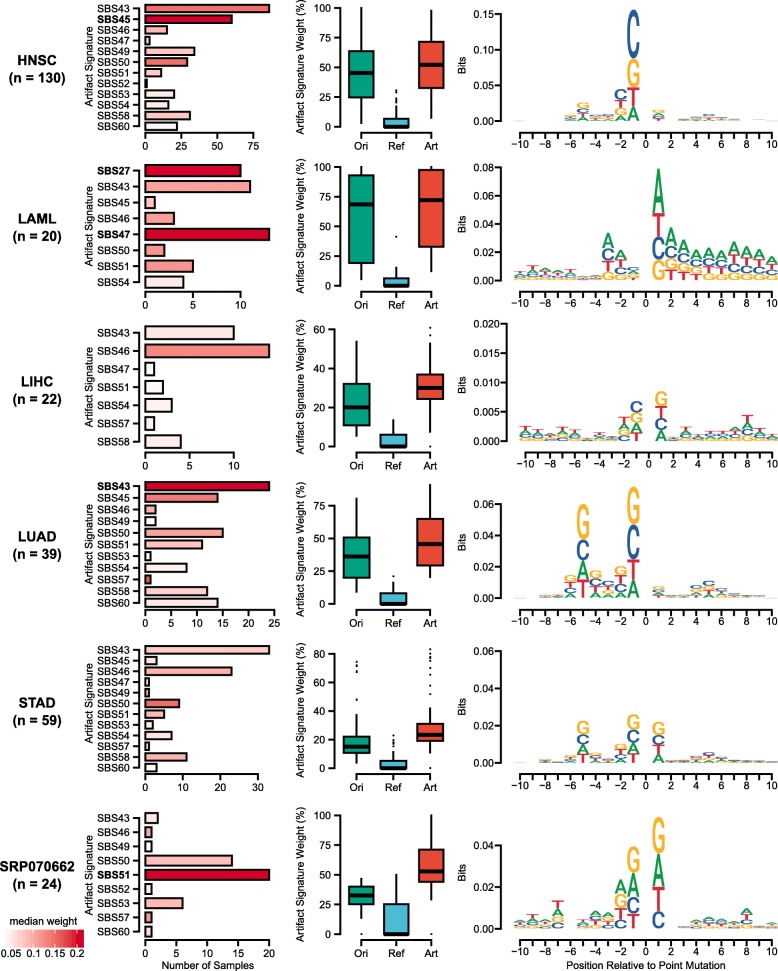
Characteristics of artifactual variants identified by FIREVAT in publicly available VCF callsets. Analysis of artifactual variants identified by FIREVAT using MuTect2 callset of multiple TCGA cohorts and multi-region WES breast cancer samples (SRP070662). From the left, the first plot for each sample group shows the profiling of the sequencing artifactual signature occurrences and weights using unrefined mutations. The bar color intensity (white to red) represents the median weight of the observed artifactual signature and the bar length represents the number of samples that had the corresponding signature as the most heavily weighted artifactual signature. The artifactual signatures with a median weight higher than 0.2 are highlighted in bold font. The second plot shows the distribution of artifactual signatures among the original (green: Ori), refined (blue: Ref), and artifactual (orange: Art) sets of mutations, respectively. The last plot shows the enrichment of sequence motifs in the artifactual variants 10 bases upstream and downstream of each variant position

Using 9 TCGA cohorts and the multi-region WES dataset (signature analysis dataset and consistency validation dataset, Additional file 1: Note S3), we further found that error-mediated signatures are widespread in publicly available VCF files (Fig. 4, Additional file 2: Figure S23). The sum of artifact signature weights varied by cancer study. For example, the median sums of artifact signature weights were 68.6% and 6.6% for acute myeloid leukemia (TCGA-LAML) and TCGA-BRCA, respectively (Additional file 3: Table S10). The signature SBS43 was the most recurrently observed among artifactual variants across the studies that had 20 or more samples, with lung adenocarcinoma (TCGA-LUAD) having the highest median weight of 22%. Certain artifact signatures were enriched in specific studies. For example, TCGA-HNSC samples had a median weight of 28.4% in SBS45, and TCGA-LAML samples had median weights of 37.4% and 37.8% in SBS27 and SBS47, respectively. Furthermore, the study-specific enrichment of artifact signatures was also observed in sequence contexts. For example, in the TCGA-LAML cohort, the artifactual mutations favored regions of repeated adenine sequences (3 bases upstream and 7 bases downstream). In the TCGA-LUAD and TCGA-STAD cohorts, guanine was redundantly found at the first and fifth bases upstream of the variant position. These cohorts shared relatively high median weights of SBS43, suggesting that the sequence context is reflective of this artifact signature. In the multi-region WES dataset (SRP070662), SBS51 was detected in 20 (83.3%) out of 24 samples with a median weight of 21.1%, while the first base immediately upstream or downstream of the variant position was commonly guanine.

**Fig. 5:**
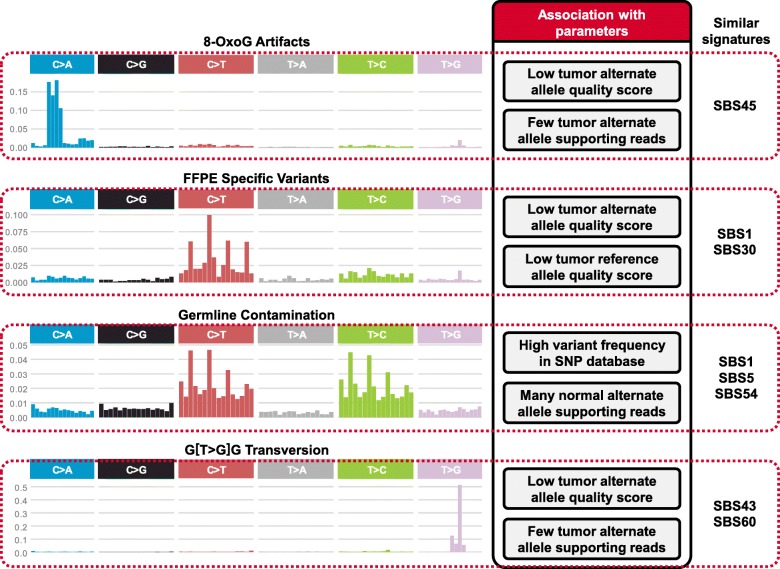
Currently known artifact signatures identified by FIREVAT in publicly available VCF callsets. There are four distinct types of artifacts that are currently identified by FIREVAT: (1) 8-oxo-guanine artifact, (2) FFPE-specific artifacts, (3) germline contamination, and (4) Thymine to Guanine (T>G) transversion artifact. The left column shows the 96 single nucleotide substitution peaks corresponding to each type of artifact. The middle column shows the quality metrics associated with each type of artifact and the last column lists the COSMIC mutational signatures (version 3) similar to the peaks observed in each artifact

Furthermore, we investigated and summarized the currently known artifact signatures that FIREVAT effectively removes via its refinement. In short, there are four types of artifacts that FIREVAT accurately identifies and filters out: (1) 8-oxoG artifacts (Additional file 4), (2) FFPE-specific artifacts (Additional file 5), (3) germline contamination (Additional file 6), and (4) Thymine to Guanine transversion artifacts in Guanine-rich context (i.e., G [T>G] G substitution) (Fig. 5, Additional file 7). Detailed analysis on each type of artifact found that these artifact signatures are associated with variants supported by low base quality scores and by a lack of alternate allele read evidence (Additional file 1: Note S5).

## Discussion

FIREVAT is the first publicly available software toolkit that performs variant refinement guided by mutational signatures. This software is easy to install and is implemented as an R package. We have herein shown the high performance of our novel variant refinement approach. Our software only requires VCF files and simplifies existing variant refinement processes, which often require the computationally expensive interrogation of BAM files [64] and manual inspection of candidate variants [20]. Such inspection also necessitates well-trained bioinformaticians. In contrast, FIREVAT automates variant refinement using single nucleotide substitution profiles of sequencing artifacts. In addition, we have shown that sequencing artifacts incorporated in mutational patterns have distinct contexts and can be correctly distinguished. FIREVAT effectively reduces the number of false positives in user-supplied VCF files and renders comprehensive reports that detail the refinement processes and outcomes.

Post-processing of variant calling is an indispensable step in the conventional cancer genomics analysis pipeline that ensures retention of high-quality real somatic variants and removal of artifactual variants. Inclusion of artifactual variants in downstream analysis leads to clinical errors and misinformed discovery of novel biological findings in research [16–19]. Variant calling by multiple callers and a series of filtering tasks have shown high levels of sensitivity and specificity [37, 63]. However, the massive computational resources required to operate such tasks are burdensome [64]. Here we have demonstrated the high performance of FIREVAT on three independent real-world datasets with 678 tumor samples. We have shown that mutational signatures can be used to strategically remove artifactual variants. FIREVAT successfully achieves this task while taking advantage of sequencing artifact patterns observed in over 23,000 samples, namely the COSMIC mutational signatures (version 3) [23].

Mutational signature analysis lends biologically and clinically relevant etiologies and concisely captures mutations observed in cancer genomes [65]. For this reason, this technique is widely used in cancer research. Based on this comprehensibility, the FIREVAT evaluation method can also be used as a proxy for data quality control in various steps of conventional cancer genomics pipelines, ranging from initial variant calling to variant refinement. FIREVAT will have broad applicability in future research studies that rely on accurate mutational signature analysis. FIREVAT is also able to handle custom matrices of mutational signatures. This allows versatile identification of diverse mutational patterns, including platform-specific artifact signatures previously unreported before. FIREVAT can be used to evaluate whether a particular sample should be included in downstream analysis based on the sum of sequencing artifact signature weights. The samples that were found to have certain sequencing artifact signatures were indeed blacklisted for having poor sequencing data quality [23]. FIREVAT will be of great utility for cancer biologists, bioinformaticians, and clinicians because it can run conveniently on a personal laptop with limited resources and streamline the multitude of computational tasks popularly performed today. With FIREVAT, a quick quality assessment of variants identified from sequencing data is possible.

Furthermore, accurate signature analysis is implicated in potential predictions of therapeutic responses in cancer. For instance, the signature related to HR deficiency is known to predict the responses of breast and pancreatic cancer patients to platinum therapy [57, 58]. The APOBEC-mediated signature is also known to predict the responses of NSCLC patients to immunotherapy [66]. FIREVAT accurately separates biological signals and technical noise by identifying enriched peaks reflective of sequencing artifacts, effectively discriminating variants while optimizing filtering cutoff parameters. Our validation study on the MC3 dataset showed that FIREVAT dynamically adjusts the filtering stringency based on the prevalence of artifactual mutations. In addition, the HNSC samples as well as the STAD sample whose mutational patterns were dramatically altered before and after FIREVAT refinement exemplify the clinical utility of our software toolkit.

In addition, we have shown that the sequencing artifacts incorporated in mutational patterns have distinct contexts and can be correctly isolated. FIREVAT uses this novel analytical method to effectively reduce the number of false positives in user-supplied VCF files and renders comprehensible reports that detail the refinement processes and outcomes. Of note, the mutational signature-based variant refinement strategy implemented in FIREVAT can be more broadly applied as a signature extraction tool. For example, our FIREVAT approach can be used to identify ranges of VAF that are enriched in biologically and clinically important signatures such as SBS2, SBS3, SBS4, and SBS13 (Additional file 1: Note S6).

Existing variant filtering approaches often require a computationally expensive interrogation of BAM files and manual inspection of candidate variants [20]. In contrast, FIREVAT automates the post hoc variant refinement process using the 96 single nucleotide substitution profiles of sequencing artifacts while optimizing cutoff parameters for each tumor sample.

To use FIREVAT, some prerequisite conditions should be met. For example, accurate deconvolution of the aggregated characteristics of mutation calls requires a sufficient number of mutations from a VCF file [32]. Second, sufficient supporting evidence on each mutation should be provided for accurate variant refinement. For instance, as previously reported, the variant allele fraction is one of the most important features for accurate variant refinement [21]. In the MC3 validation study using the Varscan callset, FIREVAT imposed more stringent filtering parameters and resulted in a higher percentage of filtered mutations than the originally identified sum of artifact signature weights. Third, a subset of the latest COSMIC mutational signatures, such as SBS12 and SBS46, have similar punctuated trinucleotide substitution peaks and exemplify challenges in signature analysis. This problem may be aggravated by a smaller number of mutations, such as those obtained from targeted sequencing. Recently, the development of a mutational signature analysis toolkit that leverages machine learning has managed to resolve this issue for the HR-deficiency signature [67]. Similar approaches can be applied to the task of mutational signature guided variant refinement to further improve differentiation between signatures.

Going forward, as mutational signatures become more exhaustive and sensitive to biological, clinical, and experimental patterns in cancer mutations, we anticipate that the FIREVAT performance will concurrently improve its performance. In this light, FIREVAT lays the foundation for variant refinement based on mutational signatures and the approaches described herein suggest the feasibility of a persistent evaluation method for variant refinement. Going forward, the FIREVAT approach can be similarly applied to small insertions and deletions (indels) [23], copy number alterations [68], and structural variations if sufficient evidence of the effects of sequencing artifacts can be profiled at these genomic levels.

## Conclusions

In conclusion, we have developed a publicly available software toolkit that efficiently removes artifactual variants in cancer samples using mutational signatures. We have shown that mutational signatures can be used as a variant refinement strategy. Our novel FIREVAT approach, which we have validated to perform highly on 384 tumor samples, should hereafter be widely used. FIREVAT secures reliability in refining mutations called from widely used variant callers and outperforms existing manual filtering methods while addressing the issue of arbitrarily determined hard-filtering parameters. The FIREVAT refinement process is streamlined for users with the call of a single function using VCF files, and presents a conveniently accessible quality control report to the user.

## Availability and requirements

The availability and requirements are listed as follows:

Project name: FIREVAT

Project home page: https://github.com/cgab-ncc/FIREVAT

Archived version: https://github.com/cgab-ncc/FIREVAT/releases/tag/v0.4.2

Operating system(s): Linux, Windows or MacOS

Programming language: R

Other requirements: R (version > = 3.5.0)

License: MIT.

Any restrictions to use by non-academics: none

## Supplementary information

**Additional file 1.** Note S1. Need for sequencing artifact signature guided variant refinement, Note S2. FIREVAT configuration file, Note S3. Overview of FIREVAT validation studies, Note S4. Additional benchmarking studies, Note S5. Characteristics and signatures of artifacts in conventional tumor sequencing, Note S6. An example of broader utility of FIREVAT, Method S1. FIREVAT objective functions, Method S2. Validation refinement evaluation methods, Method S3. R sessionInfo for validation and downstream analyses scripts.

**Additional file 2.** **Figure S1 to S4.** Artifactual signatures in the TCGA (MC3) dataset, **Figure S5.** Spectrums of mutational signatures related to sequencing artifact, **Figure S6.** Hierarchical clustering result of the mutational signatures, **Figure S7.** Benchmark test results of objective functions and GA parameters, **Figure S8 to S10.** Convergence of filter parameters in the FIREVAT refinement, **Figure S11 to S13.** Correlation between FIREVAT performance and the artifactual signature weights, **Figure S14 to S17.** Scatterplots of performance evaluation metrics on the MC3 validation dataset from the FIREVAT and other post variant-caller filtering methods, **Figure S18 and S19.** FIREVAT refinement on the multi-region whole exome sequencing data of breast cancer cases, **Figure S20.** Before and after FIREVAT refinement on the TCGA-HNSC, **Figure S21.** Before and after FIREVAT refinement on the TCGA platinum therapy responder and non-responder samples, **Figure S22.** FIREVAT results of TCGA-FP-8211, **Figure S23.** Characteristics of artifactual variants in TCGA-BRCA, TCGA-GBM, TCGA-KIRC, and TCGA-PAAD.

**Additional file 3. Table S1.** Summary of artifact signatures in publicly available callsets, **Table S2.** FIREVAT VCF attribute usage and configuration by callset on the mc3 performance validation dataset, **Table S3.** MC3 performance validation dataset samples, **Table S4.** FIREVAT performance summary on the mc3 performance validation dataset, **Table S5.** FIREVAT performance on the mc3 performance validation dataset, **Table S6 and S7.** FIREVAT refinement on the multi-region whole-exome sequencing of breast cancer dataset, **Table S8.** TCGA-HNSC dataset before and after firevat refinement, **Table S9.** TCGA platinum therapy response dataset before and after FIREVAT refinement, **Table S10.** Characteristics of artifactual variants identified by FIREVAT in publicly available VCF callsets.

**Additional file 4.** FIREVAT Report on TCGA-CR-7399. The FIREVAT variant refinement report on the sample TCGA-CR-7399.

**Additional file 5.** FIREVAT Report on TCGA-44-2662-01B. The FIREVAT variant refinement report on the sample TCGA-44-2662-01B.

**Additional file 6.** FIREVAT Report on TCGA-EE-A29B. The FIREVAT variant refinement report on the sample TCGA-EE-A29B.

**Additional file 7.** FIREVAT Report on HCC1954. The FIREVAT variant refinement report on the sample HCC1954.

## Data Availability

The following public data were used: The MC3 dataset https://gdc.cancer.gov/about-data/publications/mc3-2017 [37]. The multi-region WES breast cancer dataset from the SRA with the accession number SRP070662 https://www.ncbi.nlm.nih.gov/sra/?term=SRP070662 [27]. The TCGA datasets from the GDC data portal https://portal.gdc.cancer.gov/ [3]. The HCC1954 cell line WGS data from the ICGC data portal https://dcc.icgc.org/releases/PCAWG/cell_lines/HCC1954 [10]. The FFPE and fresh frozen WES dataset from the SRA with the accession number PRJNA301548 https://www.ncbi.nlm.nih.gov/sra/?term=PRJNA301548 [61]. The ICGC-TCGA DREAM Somatic Mutation Calling Challenge dataset https://console.cloud.google.com/storage/browser/public-dream-data?pli=1 [62]. The COSMIC mutational signatures version 3 https://www.synapse.org/#!Synapse:syn11726602 [23]. The PCAWG Platinum mutational signatures matrix 10.1016/j.cell.2019.02.012 [50]. The ClinVar annotation database (20190211 version) ftp://ftp.ncbi.nlm.nih.gov/pub/clinvar/ [29].

## Abbreviations

BAM: Binary alignment map
BRCA: Breast invasive carcinoma
COSMIC: Catalog of Somatic Mutations in Cancer
CPU: Central processing units
EGFR: Epidermal growth factor receptor
FFPE: Formalin-fixed paraffin-embedded
FIREVAT: FInding REliable Variants without ArTifacts
GA: Genetic algorithm
GBM: Glioblastoma multiforme
GDC: Genomic Data Commons
HNSC: Head-neck squamous cell carcinoma
HR: Homologous recombination
HTML: Hypertext Markup Language
ICGC: International Cancer Genome Consortium
JSON: JavaScript Object Notation
KIRC: Kidney renal clear cell carcinoma
LAML: Acute myeloid leukemia
LAN-F: Lancet filter
LIHC: Liver hepatocellular carcinoma
LUAD: Lung adenocarcinoma
MC3: Multi-Center Mutation Calling in Multiple Cancers
MUT-F: MuTect filter
NCI: National Cancer Institute
NSCLC: Non-small cell lung cancer
PAAD: Pancreatic adenocarcinoma
PCAWG: Pan-cancer Analysis of Whole Genome
SBS: Single-base substitution
SRA: Sequence Read Archive
STAD: Stomach adenocarcinoma
TCGA: The Cancer Genome Atlas
TKI: Tyrosine kinase inhibitor
VAR-F: Varscan filter
VCF: Variant call format
WES: Whole-exome sequencing
WGS: Whole-genome sequencing
