# Supplementary material for: FIREVAT: finding reliable variants without artifacts in human cancer samples using etiologically relevant mutational signatures
Source: Genome Med. 2019 Dec 17;11:81. doi: 10.1186/s13073-019-0695-x (PMC6916105; doi:10.1186/s13073-019-0695-x)
Supplement: Supplementary file 1 — Additional file 1. Note S1. Need for sequencing artifact signature guided variant refinement, Note S2. FIREVAT configuration file, Note S3. Overview of FIREVAT validation studies, Note S4. Additional benchmarking studies, Note S5. Characteristics and signatures of artifacts in conventional tumor sequencing, Note S6. An example of broader utility of FIREVAT, Method S1. FIREVAT objective functions, Method S2. Validation refinement evaluation methods, Method S3. R sessionInfo for validation and downstream analyses scripts. [file 13073_2019_695_MOESM1_ESM.docx]

**SUPPLEMENTARY NOTES AND METHODS**

**FIREVAT: finding reliable variants without artifacts in human cancer samples using etiologically relevant mutational signatures**

**Hyunbin Kim^1,*^, Andy Jinseok Lee^1,*^, Jongkeun Lee^1^, Hyonho Chun^2^, Young Seok Ju^3^, and Dongwan Hong^1,#^**

**Affiliations**

1. Bioinformatics Analysis Team, National Cancer Center, Goyang 10408, Republic of Korea
2. Department of Mathematics and Statistics, Boston University, Boston, MA 02215, USA
3. Graduate School of Medical Science and Engineering, Korea Advanced Institute of Science

and Technology, Daejeon 34141, Republic of Korea

* Co-first authors with equal contribution

# Correspondence

**Address for correspondence**

Dongwan Hong, PhD

Chief Researcher

Bioinformatics Analysis Team, National Cancer Center,

323 Ilsan-ro, Ilsandong-gu, Goyang-si, Gyeonggi-do 10408, Republic of Korea

E-mail: dwhong@ncc.re.kr

Tel: +82-31-920-2433

Fax: +82-31-920-2006

**Table of Contents**

**Supplementary Notes**

**Note S1.** Need for Sequencing Artifact Signature Guided Variant Refinement

**Note S2.** FIREVAT Configuration File

**Note S3.** Overview of FIREVAT Validation Studies

**Note S4.** Additional Benchmarking Studies

**Note S5.** Characteristics and Signatures of Artifacts in Conventional Tumor Sequencing

**Note S6.** An Example of Broader Utility of FIREVAT

**Supplementary Methods**

**Method S1.** FIREVAT Objective Functions

**Method S2.** Validation Refinement Evaluation Methods

**Method S3.** R sessionInfo for Validation and Downstream Analyses Scripts

**References**

**Supplementary Notes**

**Note S1. Need for Sequencing Artifact Signature Guided Variant Refinement**

We first performed mutational signature analysis on over 11,000 The Cancer Genome Atlas (TCGA) samples using the 65 COSMIC mutational signatures (version 3) and MutationalPatterns. The analysis of unrefined point mutations showed that sequencing artifact signatures are widespread across all 33 cancer types with varying degrees of contribution weights depending on the variant caller used to generate the callsets (Additional file 2: Figures S1-4). The unrefined mutations called by Varscan yielded the highest median artifact signature weight sum of 0.317, followed by SomaticSniper (0.128), Muse (0.112), and MuTect (0.0833) (see the table below). In particular, signature SBS58 was observed in 9,058 (82.5%) samples (contribution weight ≥ 0.05) in Varscan mutations with a median weight of 0.143. Some artifact signatures were observed at higher proportion in certain cancer types. For example, signature SBS46 was present in over 60 kidney chromophobe cancer samples (TCGA-KICH) in both Muse and Varscan callsets with a median weight of 0.255 and 0.236, respectively. Similarly, signature SBS49 was recurrently identified with high median weights in the melanoma (SKCM), kidney renal papillary cell carcinoma (KIRP), and thyroid carcinoma (THCA) samples in both MuTect and Muse callsets. These preliminary evidences highlighted the need for variant refinement guided by sequencing artifact signatures for a more accurate mutational signature analysis.

**Signature Analysis Summary of TCGA (MC3) Samples Using
65 COSMIC Mutational Signatures (Version 3)**

| **Caller** | **Median sequencing artifact signature weights sum** | **Number of samples with nonzero sequencing artifact weights sum** | **Total number of samples** | **Total number of cancer cohorts represented** |
| --- | --- | --- | --- | --- |
| MuTect | 0.083 | 3241 | 7490 | 30 |
| Muse | 0.112 | 6745 | 10809 | 33 |
| SomaticSniper | 0.128 | 7177 | 9847 | 33 |
| Varscan | 0.317 | 10798 | 10982 | 33 |

An artifact signature was included only if its weight was 0.05 or higher.

**Note S2. FIREVAT Configuration File**

**
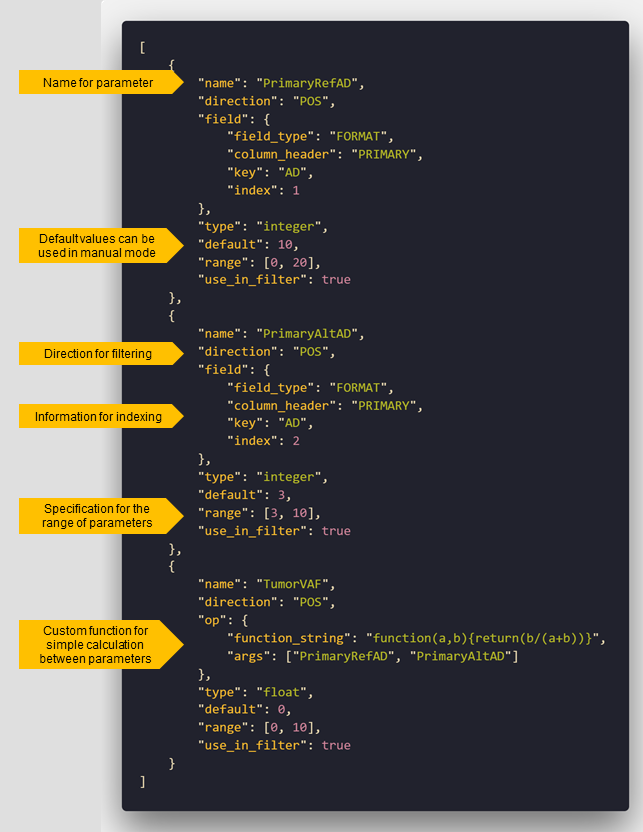
**

Different variant calling softwares often output different supporting information about each variant detected. The naming of the supporting information varies across the softwares, too. When handling VCF files, as a result, the variant attributes must be parsed manually to conduct downstream analysis, including variant refinement. To accommodate such variations in VCF files, we have developed a systematic approach to specify desired filter parameters and instructions on how to treat each filter. We have implemented this functionality in FIREVAT as an input parameter. This input parameter is supplied by the user as a FIREVAT configuration (JSON) file. Users are able to either use the default configuration files provided in our FIREVAT R package or choose to create their own custom configuration setups. Shown above is an example structure of the FIREVAT configuration file.

**Note S3. Overview of FIREVAT Validation Studies**

# **
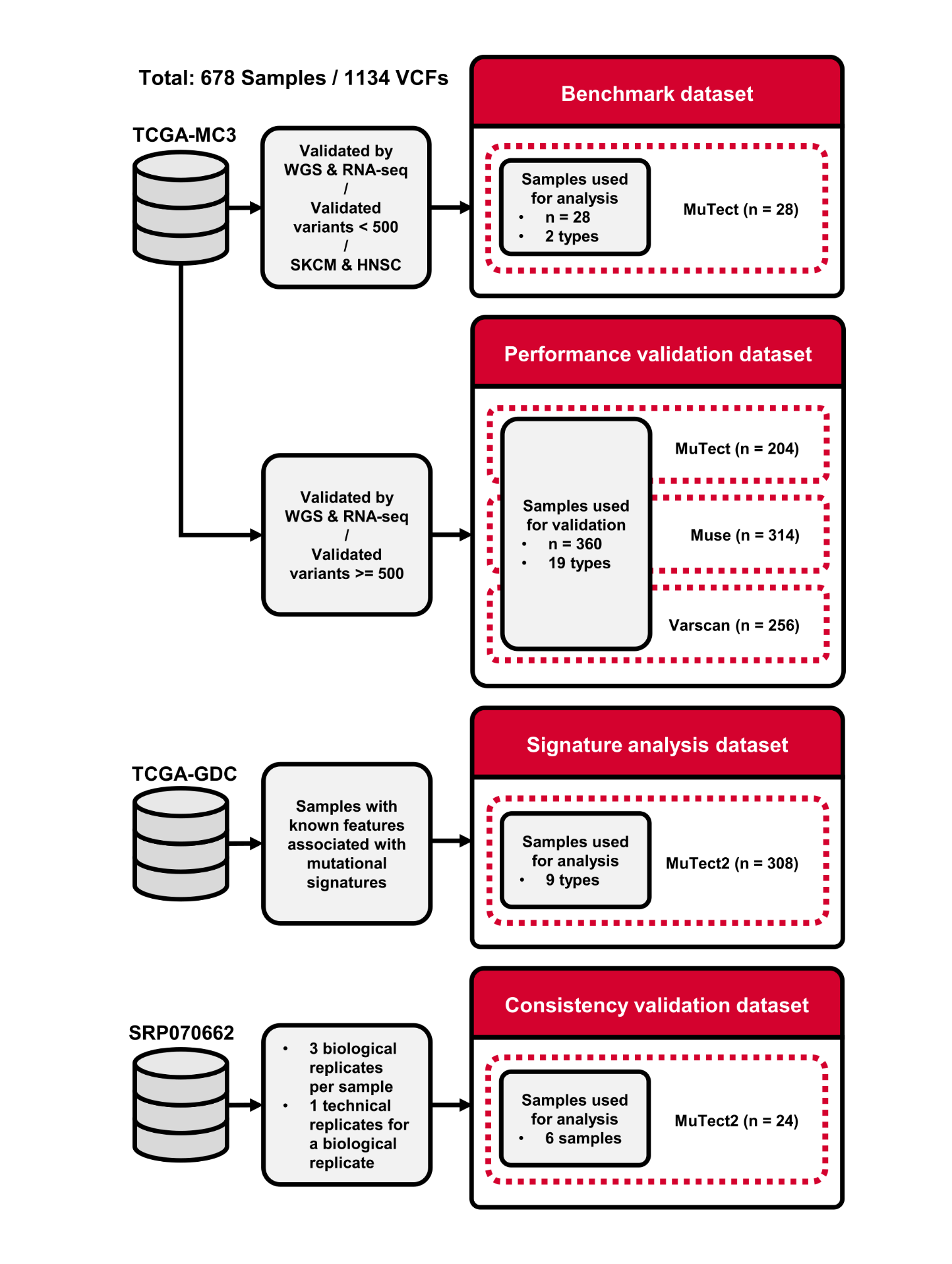
**

We evaluated FIREVAT on three different objectives: variant refinement performance (validity), signature analysis accuracy (validity), and consistency (reliability). We used the following datasets to conduct the validation studies: (1) MC3 dataset, (2) TCGA-GDC dataset, and (3) multi-region whole-exome sequencing of breast cancer dataset (SRP070662).

1. **Benchmark dataset.** A total of 28 MC3 samples were used to test FIREVAT parameters.
2. **Performance validation dataset.** A total of 360 MC3 samples were used to evaluate FIREVAT variant refinement performance. The MC3 dataset is comprised of variants detected in multiple sequencing methods for each sample and facilitates determination of variants that are clonal true positives. Such information allows for variant refinement evaluation against ground truth. We evaluated FIREVAT on three different VCF datasets, each called from a different variant calling software: MuTect (n = 204), Muse (n = 314), and Varscan (n = 256).
3. **Signature analysis dataset.** We used 308 TCGA-GDC samples to perform signature analysis using point mutations before and after FIREVAT variant refinement. The clinical annotation data for each cancer sample (e.g., tobacco smoking history) allows evaluation of concordance between signature analysis results and the known clinical history. The cancer types represented in this dataset were head and neck cancer (HNSC, n = 130), stomach cancer (STAD, n = 59), lung adenocarcinoma (LUAD, n = 39), liver hepatocellular carcinoma (LIHC, n = 22), acute myeloid leukemia (LAML, n = 20), glioblastoma multiforme (GBM, n = 11), breast cancer (n = 10), pancreatic cancer (n = 10), and kidney renal clear cell carcinoma (KIRC, n = 7).
4. **Consistency validation dataset.** We used the 24 multi-region whole-exome sequencing samples (18 biological replicates and 6 technical replicates) obtained from six breast cancer patients (SRP070662) to evaluate whether FIREVAT is able to eliminate false positives known to be abundant in the intratumoral heterogeneity analysis dataset. The candidate variants initially identified in this dataset were independently validated using targeted sequencing.

**Cancer Types in the MC3 (Performance Validation) Dataset**

| **Cohort** | **Number of Samples** |
| --- | --- |
| BLCA | 12 |
| BRCA | 34 |
| CESC | 5 |
| COAD | 27 |
| DLBC | 3 |
| GBM | 2 |
| HNSC | 24 |
| KICH | 4 |
| KIRC | 1 |
| LGG | 1 |
| LIHC | 17 |
| LUAD | 95 |
| LUSC | 36 |
| READ | 5 |
| SARC | 15 |
| SKCM | 38 |
| STAD | 15 |
| UCEC | 24 |
| THCA | 2 |

**Note S4. Additional Benchmarking Studies**

As a signature based variant filtering tool which utilizes the weights of artifact signature to evaluate filtering performance, we have performed additional analyses to study the extent to which FIREVAT removes true positives.

1) ICGC-TCGA-DREAM Somatic Mutation Calling Challenge

First, we performed a benchmark study against other published methods in the ICGC-TCGA-DREAM Somatic Mutation Calling Challenge [1] using the set 1. We conducted somatic mutation calling with MuTect, Muse, and Varscan with the default settings and additionally MuTect with panel of normal and the TLOD option to compare with submitted refinement methods. Among the methods submitted to DREAM challenge, pipelines that used custom alignment and BAM file generation or callers other than MuTect, Muse and Varscan were excluded from our comparative analysis comparison in order to objectively evaluate the post-hoc filtering performance of FIREVAT. We also compared DToxoG [2] results for all of the callsets that had read count information for each strand.

| **Method_name** | **TP** | **FP** | **FN** | **Precision** | **Recall** | **F1_score** |
| --- | --- | --- | --- | --- | --- | --- |
| Varscan_RAW | 3507 | 26413 | 30 | 0.117 | 0.992 | 0.210 |
| Varscan_FIREVAT | 3459 | 14271 | 78 | 0.195 | 0.978 | 0.325 |
| Varscan_FIREVAT_leaky_objfn | 3377 | 12882 | 160 | 0.208 | 0.955 | 0.341 |
| Muse_RAW | 3451 | 5294 | 86 | 0.395 | 0.976 | 0.562 |
| Muse_FIREVAT | 3432 | 1254 | 105 | 0.732 | 0.970 | 0.835 |
| Muse_FIREVAT_leaky_objfn | 3384 | 1128 | 153 | 0.750 | 0.957 | 0.841 |
| MuTect_RAW | 3506 | 58027 | 31 | 0.057 | 0.991 | 0.108 |
| MuTect_PASS | 3468 | 1942 | 69 | 0.641 | 0.980 | 0.775 |
| MuTect_RAW_FIREVAT | 3445 | 13359 | 92 | 0.205 | 0.974 | 0.339 |
| MuTect_PASS_FIREVAT | 3465 | 1295 | 72 | 0.728 | 0.980 | 0.835 |
| MuTect_RAW_FIREVAT_leaky_objfn | 3347 | 10895 | 190 | 0.235 | 0.946 | 0.377 |
| MuTect_PASS_FIREVAT_leaky_objfn | 3462 | 1281 | 75 | 0.730 | 0.979 | 0.836 |
| MuTect_PASS_PoN_TLOD10 | 3438 | 1108 | 99 | 0.756 | 0.972 | 0.851 |
| MuTect_PASS_PoN_TLOD10_FIREVAT | 3398 | 732 | 139 | 0.823 | 0.961 | 0.886 |
| MuTect_PASS_DToxoG | 3468 | 1747 | 69 | 0.665 | 0.980 | 0.793 |
| Varscan_DToxoG | 3451 | 25415 | 86 | 0.120 | 0.976 | 0.213 |

We found that FIREVAT refinement increased precision by 16.65% on average for the three variants lists obtained from three different callers while decreasing sensitivity by 1.14% on average (maximum of 1.98%) with the original unrefined variants list as the benchmark. FIREVAT achieved the highest sensitivity and precision levels in the Muse callset. Besides the methods that used visual inspection and panels of normal samples, which are not publicly available, FIREVAT achieved the highest level of precision while yielding a sensitivity level (96.07%) that was only 1.35% lower than the method that achieved the highest level of sensitivity (98.05%) in the MuTect callset. In the Varscan callset, FIREVAT’s sensitivity (97.79%) was 1.61% lower than the highest performer for sensitivity (99.41%).

**
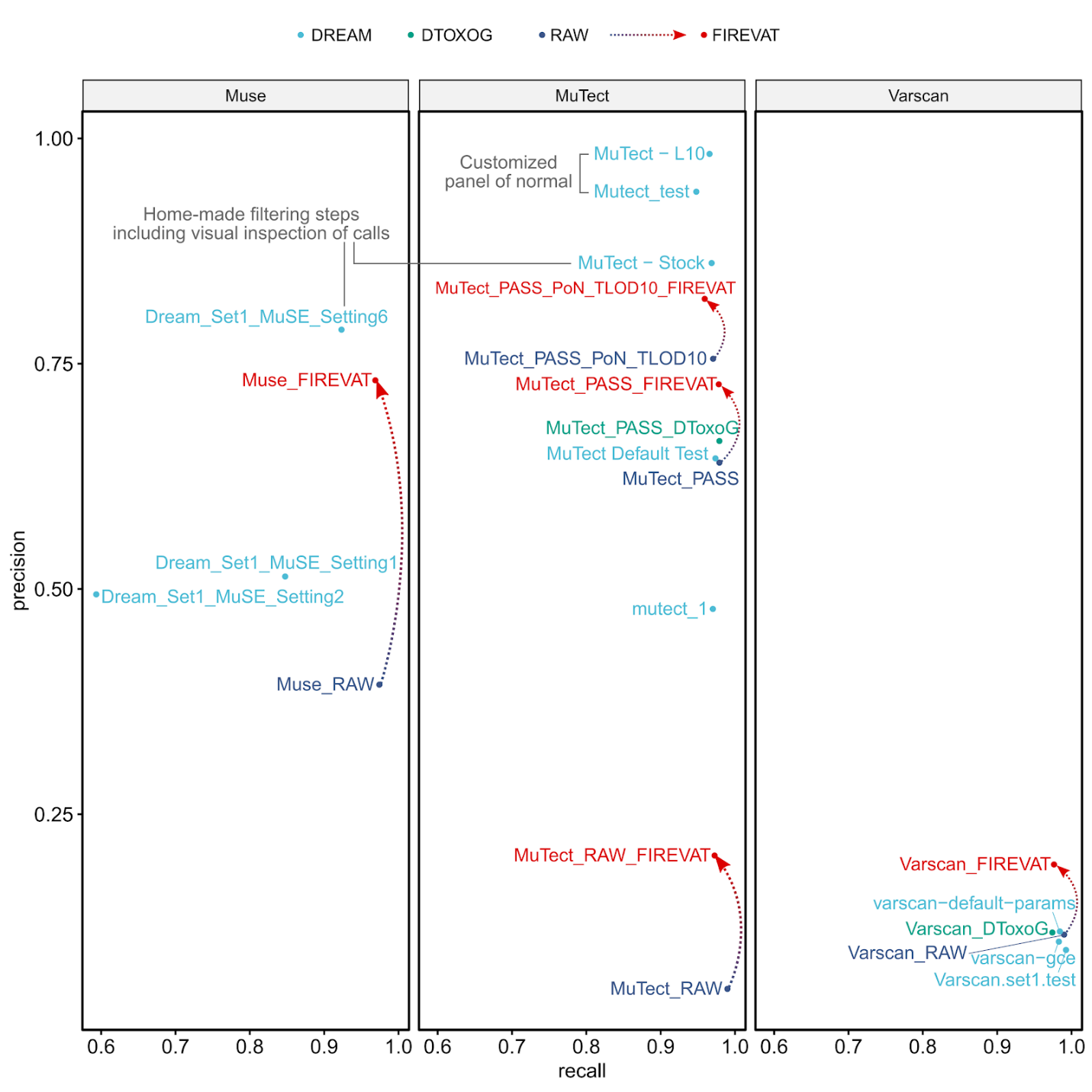
**

Moreover, FIREVAT increased both the precision and the F1 score from the raw lists of variants called. FIREVAT ranked second in F1 scores of methods using Muse. The Dream_Set1_MuSE_Setting6 method scored better F1 scores (0.851) than FIREVAT (0.835), but performed visual inspection as part of its refinement strategy. Compared to the Dream_Set1_MuSE_Setting6 method, variants called with Muse and filtered with FIREVAT contained more false-positives (Setting 6 = 877; FIREVAT = 1,254) but less false-negatives (Setting 6 = 266; FIREVAT = 105). FIREVAT correctly filtered out 4,040 (76.31%) false positive variants from the 5,294 false positives in raw MuSE call, while keeping 3,432 (99.45%) true positives in the refined set.

Although the default setting of Varscan did not provide base quality scores for called variants and therefore provided limited quality information, FIREVAT performed best among the methods using Varscan callset. FIREVAT removed 12,142 (45.97%) false-positives from the Varscan-called 26,413 false positive variants and incorrectly filtered out 48 (1.37%) variants of the 3,507 true positives.

FIREVAT increased the F1 score from 0.107 to 0.339 in the MuTect callset by accurately filtering out 44,668 (76.98%) false positive variants. The F1 score for FIREVAT using the raw variants list MuTect was much lower than the F1 score of 0.775 for the variants that passed the MuTect’s built-in filter. This shows that our signature-based filtering optimization method is less stringent compared to built-in filters in variant callers. However, FIREVAT application on variants that passed the MuTect’s built-in filters further improved the F1 score from 0.775 to 0.835 by filtering out 647 (33.32% of false positives in MuTect PASS variants) false positive variants and generating only 3 false negatives. With variants called from more stringent MuTect settings, FIREVAT additionally filtered out 376 false variants from the total of 1,108 false positives.

Among all MuTect called methods benchmarked, FIREVAT ranked 4th with the MuTect_PASS_PoN_TLOD10_FIREVAT setting. Two of the three methods that achieved higher F1 scores than FIREVAT used a customized panel of normal (submitted by BroadSMC team), which we could not obtain during the benchmark experiment. The other method submitted by SLC platform utilized home-made post-hoc filtering steps with 8 filters: read depth filtering, mapping quality filter, read position filter, strand bias filter, match normal filter, simple repeats filter, centromere filter and panel of normal filter.

In summary, variants filtered with FIREVAT contained more false positive variants compared to those filtered with visual inspection or home-made filtering by experts. However, the benchmarking results showed that applying FIREVAT to the output of the variant callers can reduce the false positive variants from the raw list of variants called.

2) PCAWG Platinum Signature Set

The TCGA samples were included in the extraction of the COSMIC mutational signatures version 3 [3]. We originally presented the prevalence of artifact signatures in the TCGA (MC3) callsets using the COSMIC mutational signatures (Additional file 2: Figure S1-S4). Using the same dataset from which the signatures were generated to remove artifacts can raise questions on the lack of control in concern. To address this point, we closely examined another set of mutational signatures that was published recently, namely the PCAWG Platinum signatures [4]. Although the same research group worked on the extraction of the Platinum signatures, the samples used to extract the signatures was a subset (n = 2,709) of all samples used to generate COSMIC version 3 signatures (n > 23,000). The Platinum signatures extracted 9 technology-related artifact signatures (SBSR1 to SBSR9). We examined whether these nine signatures correlate with any of the 18 signatures from COSMIC version 3 and found an analogous COSMIC version 3 artifact signature for all of the Platinum technology-related artifact signatures.


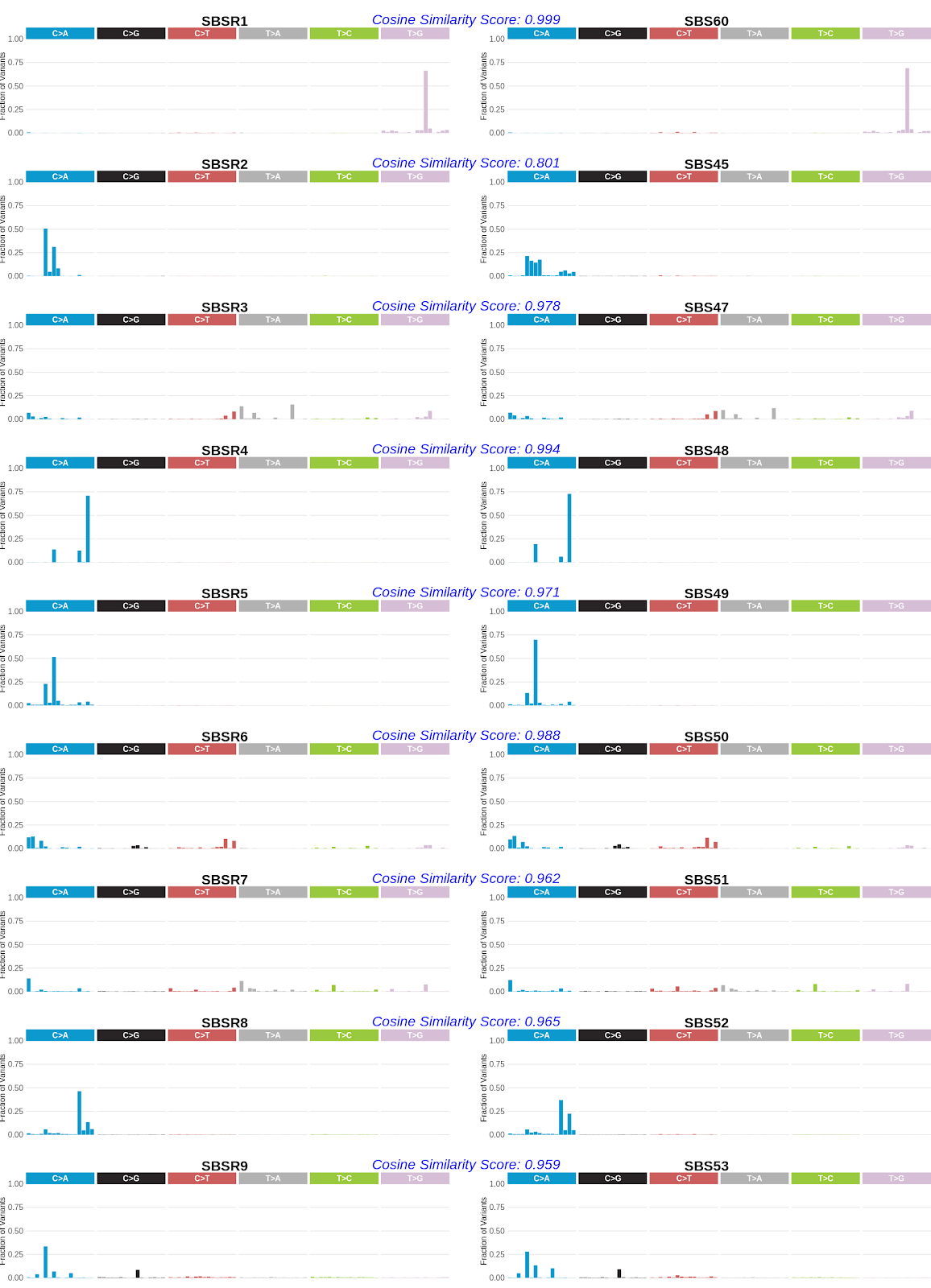


Except for SBSR2, the cosine similarity score for all of the pairs was above 0.9. The replicated extraction of highly comparable artifact signatures suggests that the trinucleotide spectrums exhibited by these mutational patterns do capture sequencing error. We therefore confirmed that the artifact signatures are not TCGA-specific.

Following these results, we further analyzed whether the reported prevalence of artifact signature weights in the unrefined mutations in the TCGA (MC3) callsets (Additional file 2: Figure S1-S4) can also be replicated. For this analysis, we identified and excluded all of the TCGA samples that were used to generate the PCAWG Platinum signatures from the MC3 validation dataset. The matching PCAWG Platinum artifact signature was detected at a similarly high contribution weight as the COSMIC version 3 signatures.


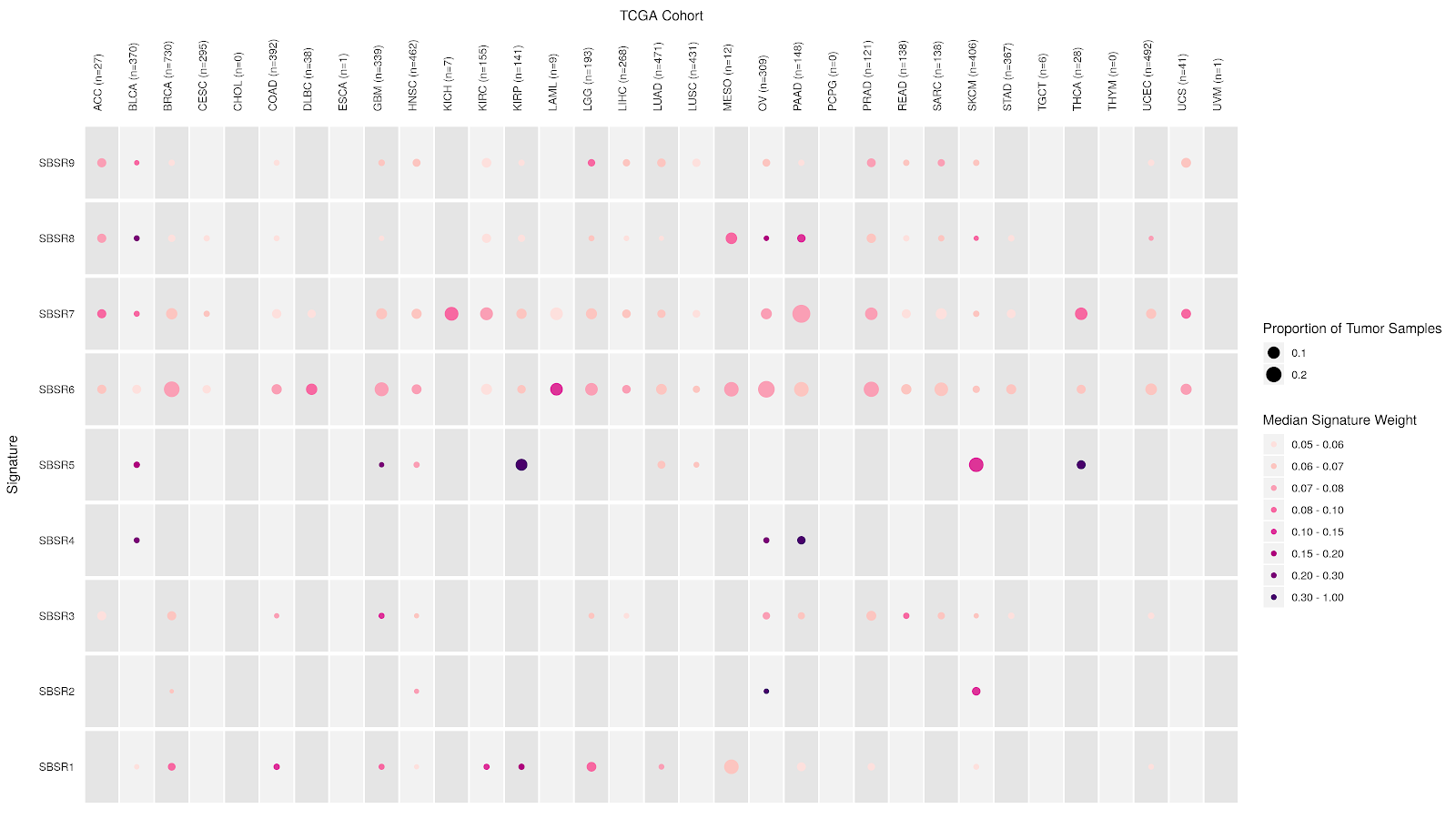


Distribution of artifact signatures reported from PCAWG platinum dataset (SBSR1 - R9) in the TCGA (MC3) MuTect dataset (n = 6,536, 30 cancer types). We identified the 6,536 MuTect-called VCFs from TCGA samples which were not included in PCAWG platinum signatures dataset. Especially, SBS R6 was observed in most of the TCGA cohorts.


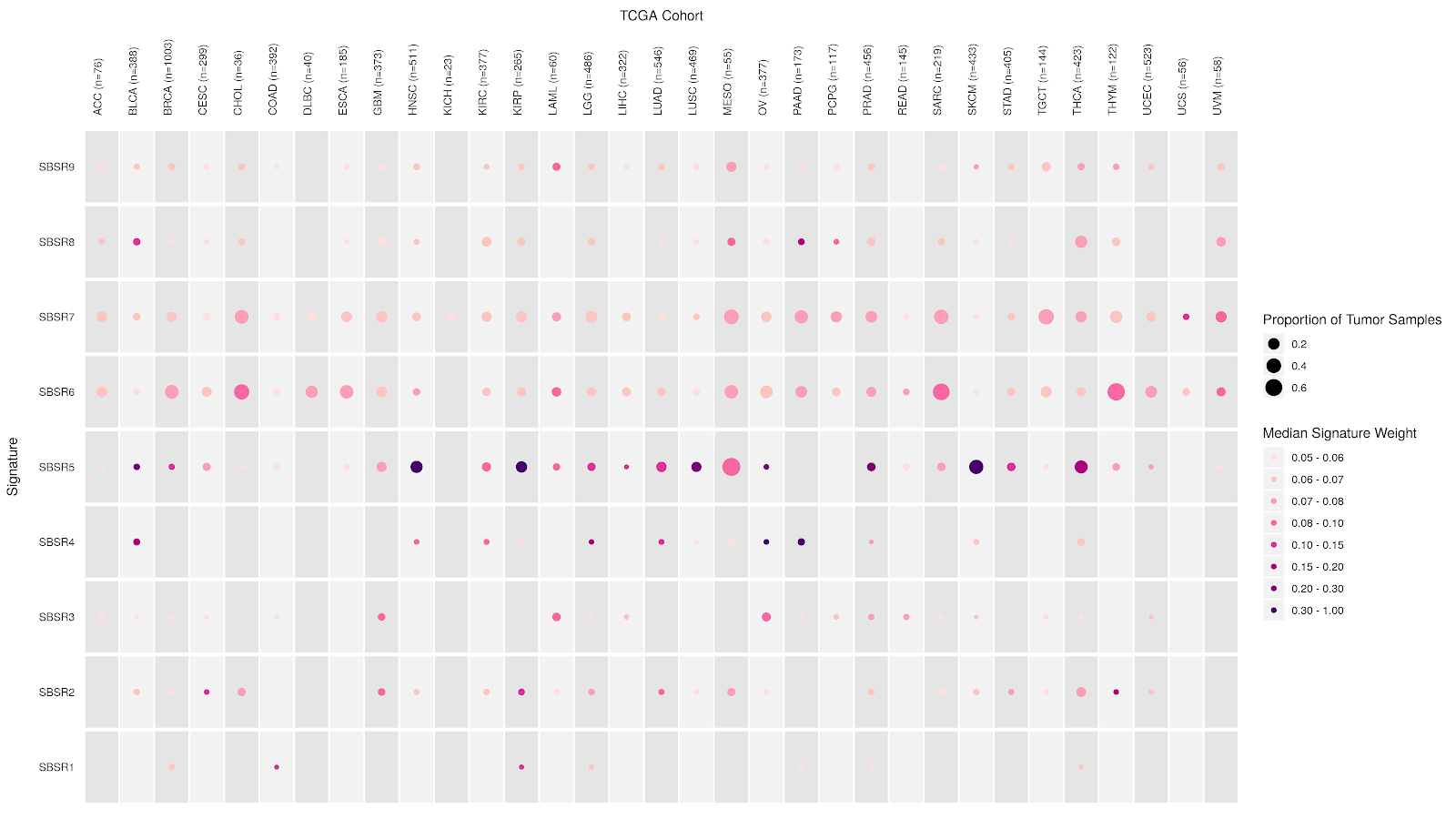


Distribution of artifact signatures reported from PCAWG platinum dataset (SBSR1 - R9) in TCGA Muse dataset (n = 9,557, 33 cancer types).


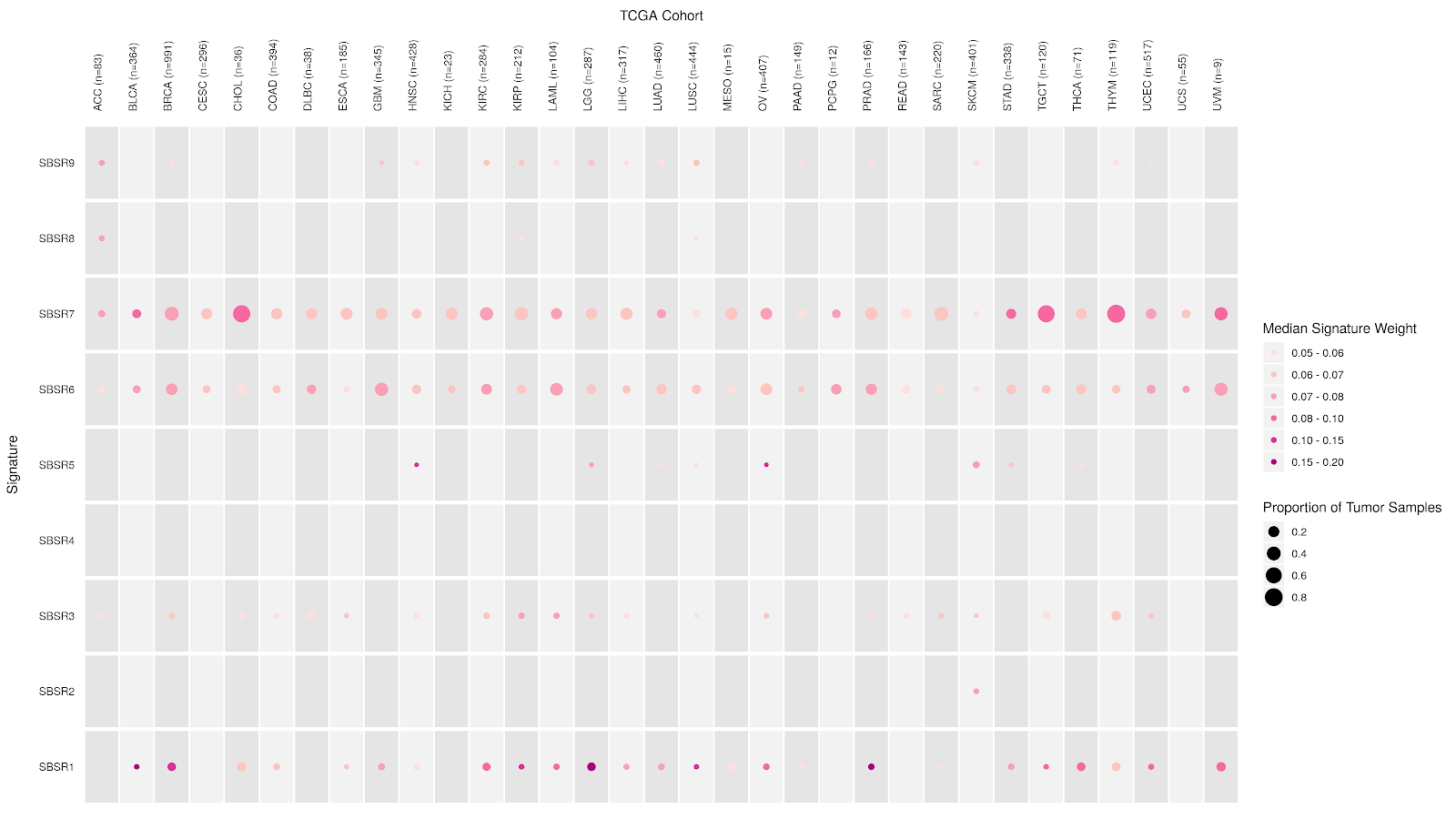


Distribution of artifact signatures reported from PCAWG platinum dataset (SBSR1 - R9) in TCGA SomaticSniper dataset (n = 8,033, 33 cancer types).


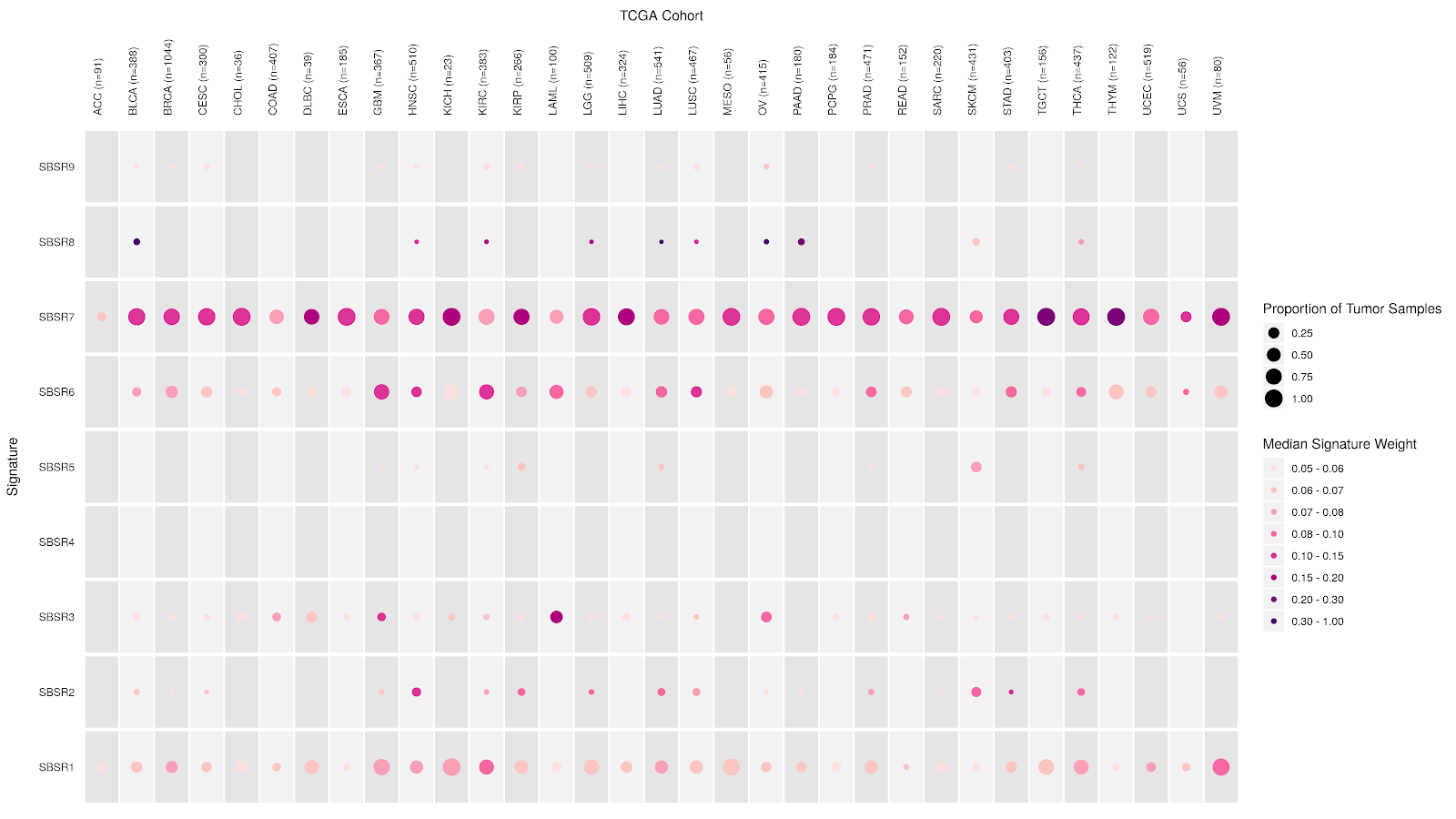


Distribution of artifact signatures reported from PCAWG platinum dataset (SBSR1 - R9) in TCGA Varscan dataset (n = 9,862, 33 cancer types).


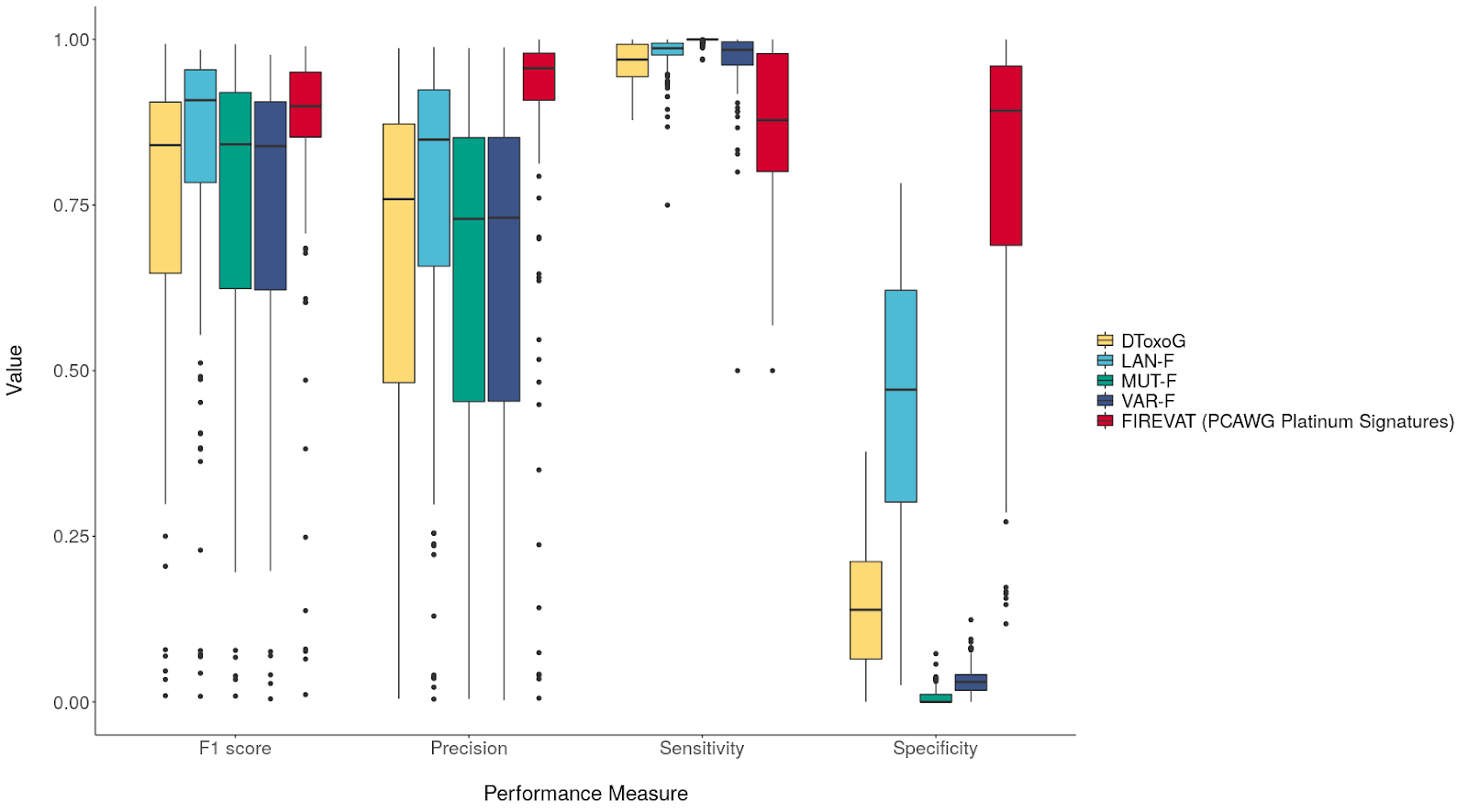


We benchmarked FIREVAT refinement performance using the Platinum signatures. Out of the originally selected 360 validation samples from the MC3 dataset, we confirmed that 157 samples were used to extract the Platinum signatures. With the remaining 203 samples, we proceeded to perform a benchmark test using the Platinum signatures against DToxoG [2] as well as the other manual filters previously included in the manuscript. FIREVAT refinement resulted in the highest precision and specificity against DToxoG and the other three manual filtering approaches. These results were commensurate with the previous results we obtained with the COSMIC signatures version 3 (Figure 2A).

3) Comparison to MuTect Built-in Filter

We analyzed the variant refinement performance using unrefined variants and variants filtered using caller’s built-in filters. We performed FIREVAT using all mutations called in the MC3 MuTect callset. Of the 360 samples in the validation dataset, we selected 102 samples that had 200,000 or less point mutations in the raw MuTect VCF file and applied FIREVAT on these. Variants subject to evaluation were the MuTect filtered (i.e. FILTER=PASS) variants. We then compared the refinement performance of these results with the results obtained from running with MuTect filtered variants for the same 102 samples. We found that sensitivity was in fact higher in the FIREVAT results obtained using MuTect PASS variants only on average (87.21% for raw VCF file and 93.67% for MuTect filtered VCF), whereas precision and specificity were higher for raw VCF files (figure shown below).

Similarly, in the DREAM challenge MuTect benchmarking results, we observed that MuTect built-in filter is necessary to reduce most of the false positives. Based on these benchmark results, we recommend the users to use FIREVAT as a post-hoc filtering tool in variant calling pipelines.


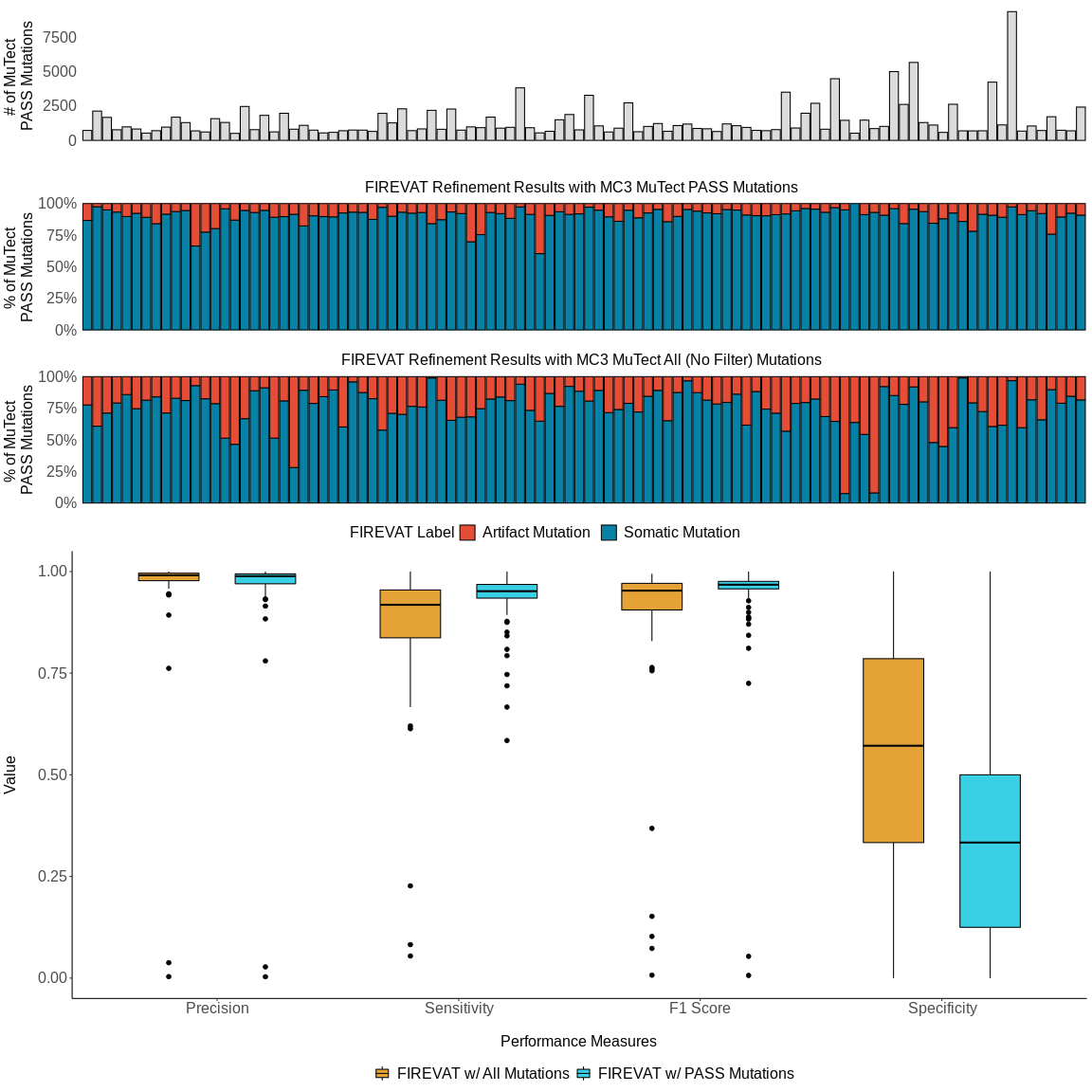


Comparison of FIREVAT variant refinement performance using the unrefined MC3 MuTect callset and the MuTect built-in filter applied callset (n =102).

**Note S5. Characteristics and Signatures of Artifacts in Conventional Tumor Sequencing**

We have further investigated the characteristics of artifacts identified by FIREVAT. As part of this additional analysis, we applied FIREVAT on formalin fixed paraffin embedded (FFPE) tissues and also evaluated FIREVAT’s capabilities in accurately filtering out germline variants. In summary, there are four distinct types of artifacts that are accurately identified by FIREVAT: (1) 8-oxo-guanine artifact, (2) Thymine to Guanine (T>G) transversion artifact, (3) FFPE artifact, and (4) germline contamination. Summarized below are the explanations and study findings on each type of artifact.


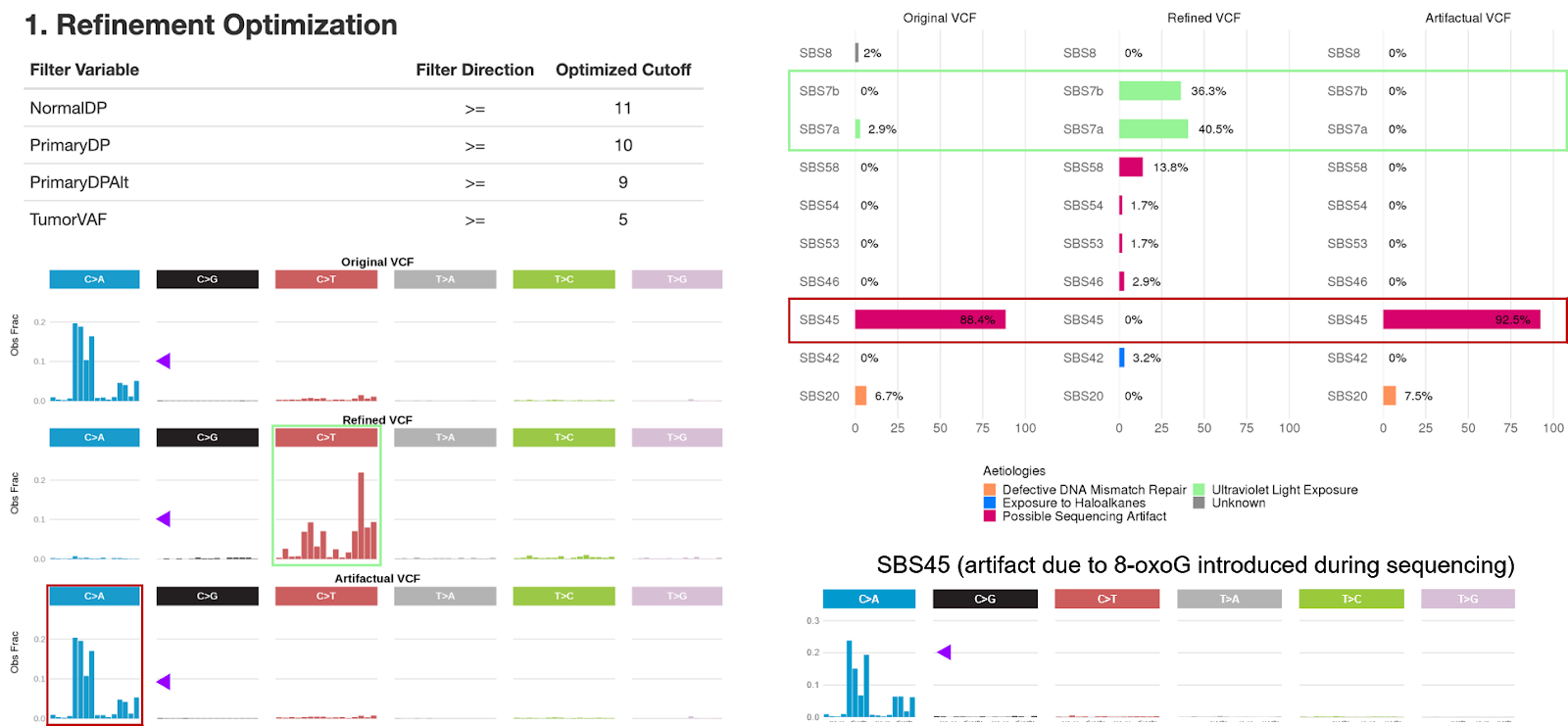


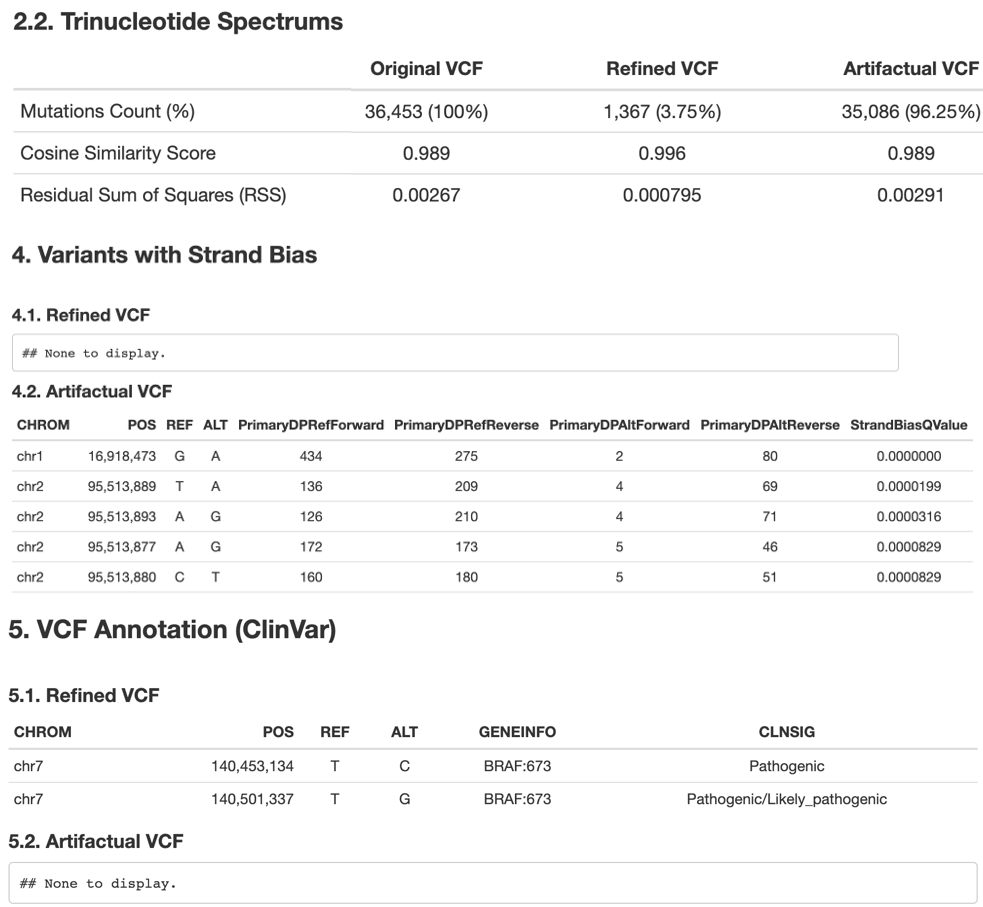


TCGA-EB-A24D FIREVAT report

1) 8-oxoG Artifact Mutations

FIREVAT is able to accurately identify 8-oxoG mutation artifacts based on identification of the C>A subtype peaks found in SBS45 [3], an artifact signature proposed to be significantly reflective of 8-oxoG introduced during sequencing. The FIREVAT refinement outcome on TCGA-EB-A24D (melanoma) best illustrates this point. The original unrefined set of variants (36,453 variants) for this sample exhibited strong peaks corresponding to SBS45 with a contribution probability of 88.4%. FIREVAT was applied to this sample and we found that SBS45 was no longer detected in the refined variants list (1,367 variants, 3.75% of unrefined set), while the same signature was enriched in the filtered-out artifact variants list (35,086 variants, 96.25%). In addition, SBS7a and SBS7b, two ultraviolet (UV) signatures that are known to be enriched in melanoma samples, only emerged in the refined variants list at 40.5% and 36.3%, respectively. ClinVar annotation of the downstream variants lists from FIREVAT further indicated accurate refinement on this sample as the pathogenic c.735A>C (p.Leu245Phe) and c.1801A>G (p.Lys601Glu) variants in BRAF are classified as somatic mutations by FIREVAT. Similarly, effective filtering of artifact mutations due to 8-oxoG was observed in the head and neck cancer sample (TCGA-CR-7399). As we have previously reported in the manuscript, unobserved smoking signature was identified in the FIREVAT refined variants set (Figure 3C). Likewise, with high fidelity FIREVAT is able to identify false somatic variants arising from 8-oxoG during sequencing.

2) Thymine to Guanine (T>G) Transversion Artifact Mutations

The Thymine to Guanine (T>G) transversion artifact mutations in Guanine-rich context have been previously described by two independent research groups [5, 6]. By identifying the G[T>G]G transversion peaks in SBS43, SBS51, and SBS60, we found that FIREVAT is able to accurately filter the transversion artifact variants. For example, in the case of HCC1954 (DKFZ), we found that the transversion peak is attenuated by filtering out variants with low VAF.


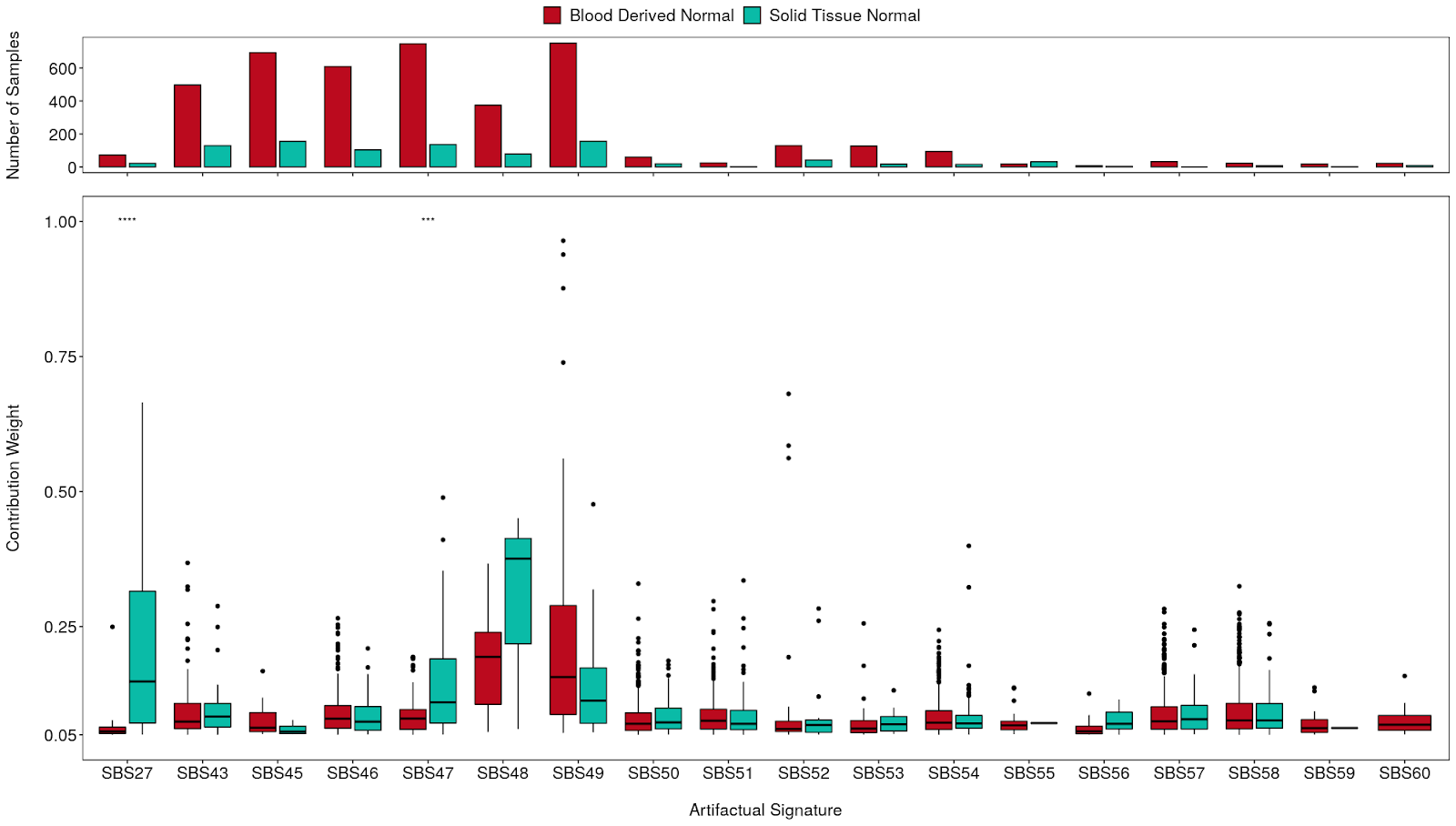


Furthermore, contribution weights of certain artifact signatures varied by the normal control types. In particular, SBS60 was only found in samples whose normal control for variant calling was derived from a blood sample in MC3 MuTect callset. The T>G transversion has been reported to be associated with control depth coverage imbalance [5]. The enrichment of SBS60 in samples with blood-derived normal control therefore might be correlated with the convention of performing sequencing at a lower coverage depth for control samples.

3) FFPE-specific artifact mutations

Conventional guidelines on accurate sequencing and analysis of tumor samples advise against the use of FFPE samples because the library preparation step induces exogenous DNA damage and introduces artifact mutations [7]. As a result, variant calling of FFPE tumor samples leads to false positives that can render downstream analyses equivocal. Comparative analyses with fresh frozen samples have shown that somatic mutations called from FFPE samples yield higher fraction of C>T substitutions compared to those called from fresh frozen tissues [8, 9]. We compared FIREVAT refinement outcomes on FFPE and non-FFPE tumor samples obtained from the same cancer patient in three independent cases. In the two TCGA-GDC cases (TCGA-44-2662 lung adenocarcinoma sample and TCGA-A7-A13E breast cancer sample), the FFPE-specific variants were effectively removed using the 18 artifact signatures from COSMIC version 3.


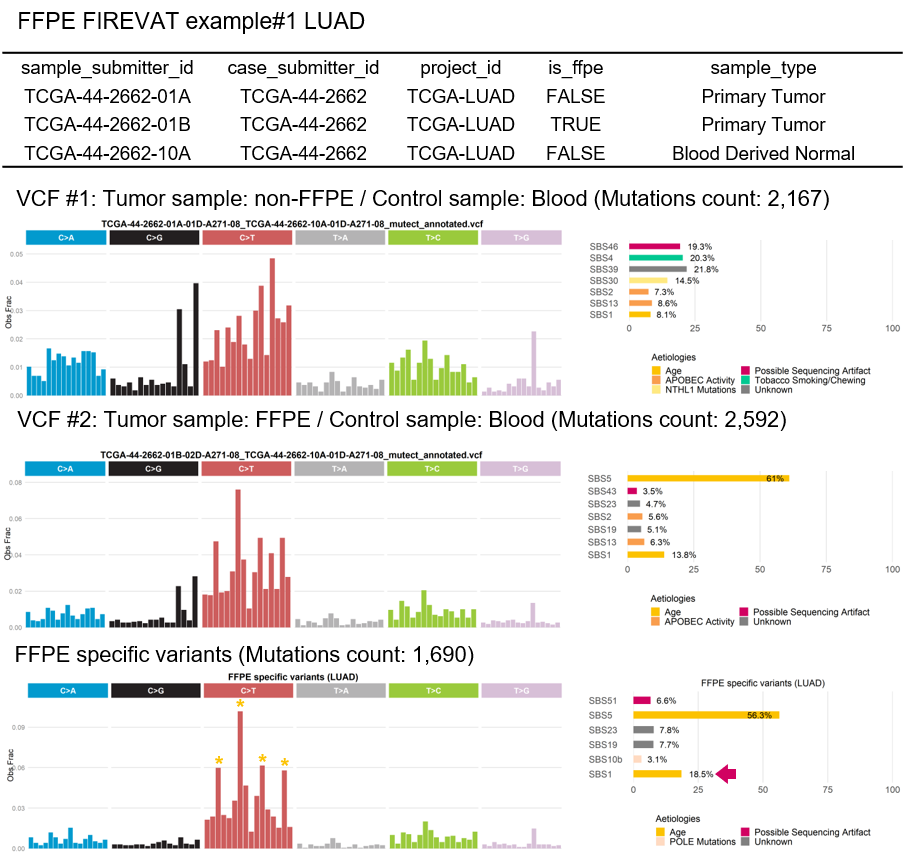


FFPE example: TCGA-44-2662-01B


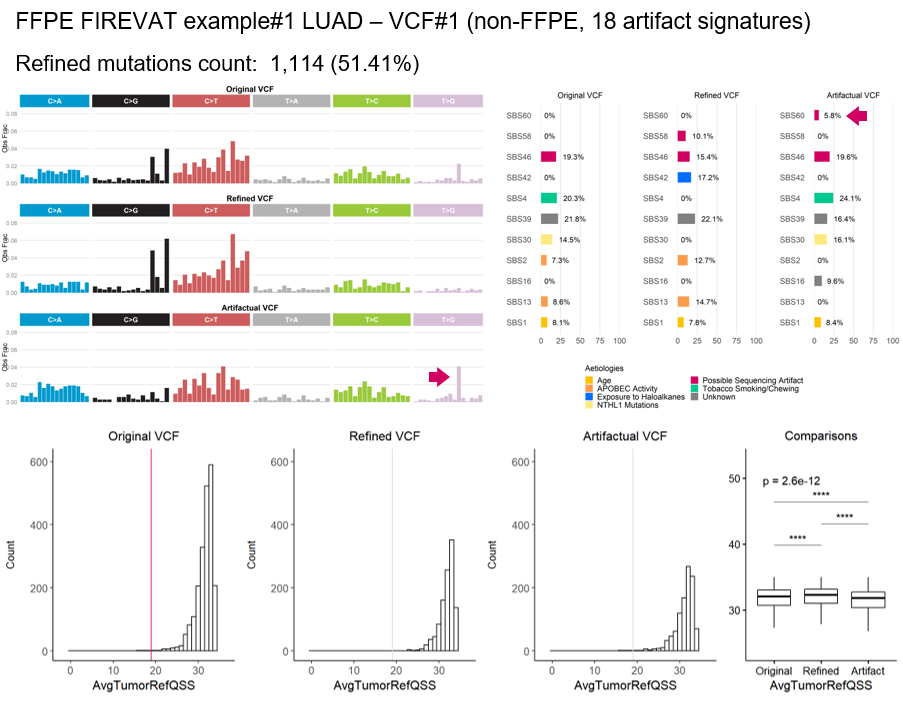


FIREVAT result of non-FFPE LUAD sample with 18 artifact signatures


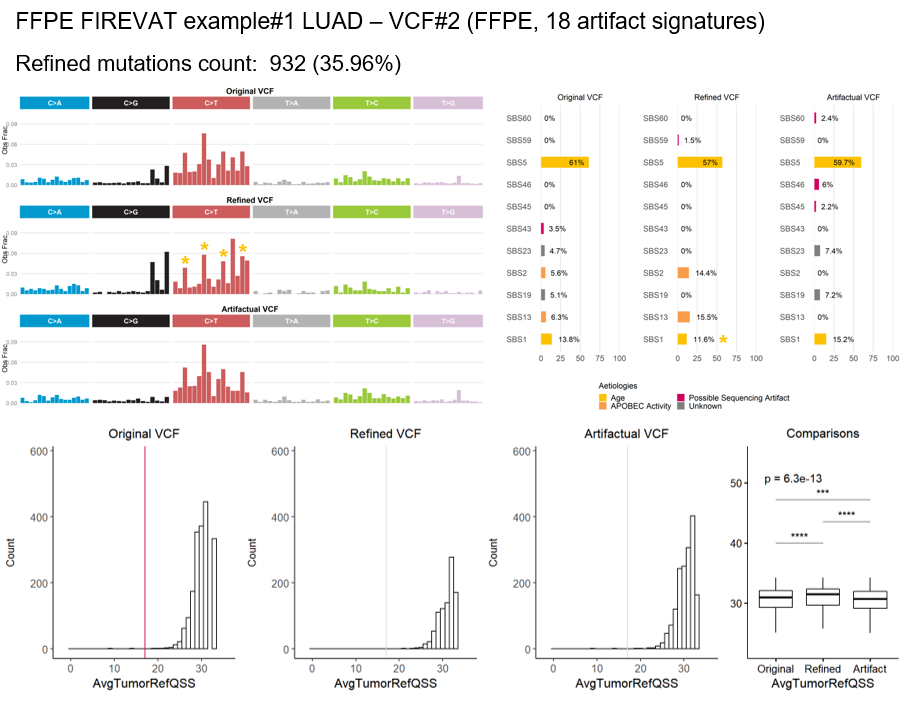


FIREVAT result of FFPE LUAD sample with 18 artifact signatures.

To begin with, there were 2,592 variants called in the TCGA-44-2662 FFPE tumor sample. Among these, 1,690 variants were determined to be FFPE-specific artifact variants compared to the non-FFPE tumor sample. FIREVAT refinement led to filtering out 1,282 (75.6%) of the FFPE-specific mutations. The cosine similarity score between trinucleotide spectrums of the FFPE-specific variants and artifact variants determined by FIREVAT was 0.990 for this sample. By visual inspection, however, the C>T spectrum in the FIREVAT refined set of mutations appeared to still include the C>T peaks found in the FFPE-specific variants for TCGA-44-2662, which resulted in relatively higher cosine similarity of 0.775 between the spectrums of FFPE-specific variants and FIREVAT refined variants. Evidently, the FIREVAT optimized average tumor reference allele quality score (AvgTumorRefQSS) cutoff was commensurate between the non-FFPE and FFPE samples for this sample: 19 for the non-FFPE sample and 17 for the FFPE sample.


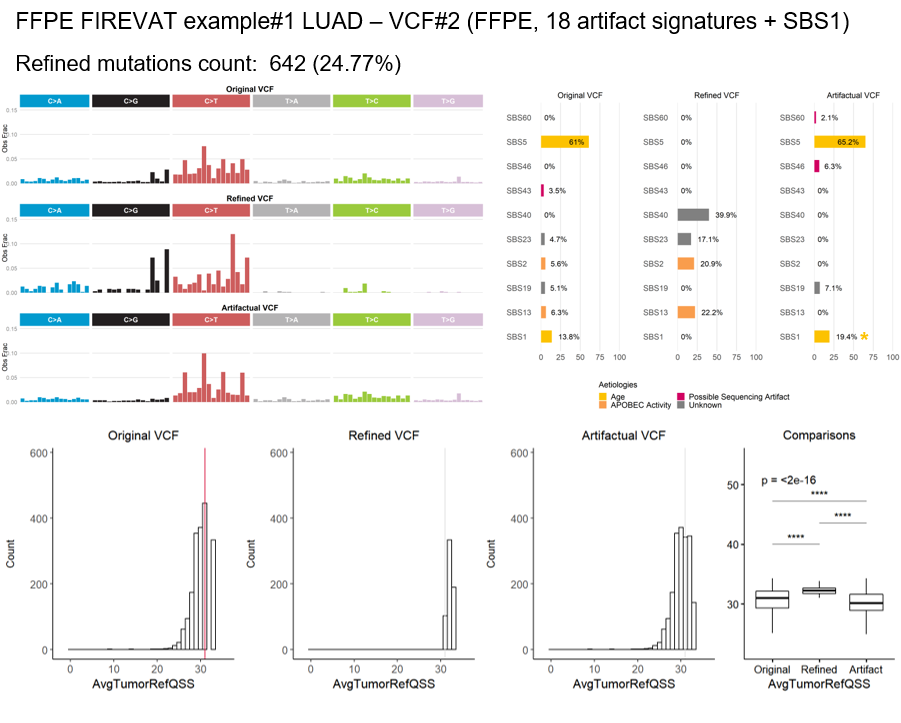


 FIREVAT result of FFPE LUAD sample with SBS1 and 18 artifact signatures

To impose stricter filtering conditions, we supplied SBS1 as well as the original 18 signatures as the input artifact signatures in order to attenuate the C>T peaks reflective of FFPE-specific variants. While maintaining high cosine similarity between FFPE-specific variants and artifact variants filtered out by FIREVAT (0.985), this experimental FIREVAT setting resulted in a lower cosine similarity score between trinucleotide spectrums of the FFPE-specific variants and the refined set of mutations: 0.775 with only 18 signatures and 0.473 with the addition of SBS1. This FIREVAT setting picked 31 as the cutoff for the quality score of reference allele in tumor, which is much higher than 17 in the setting of 18 artifact signatures.


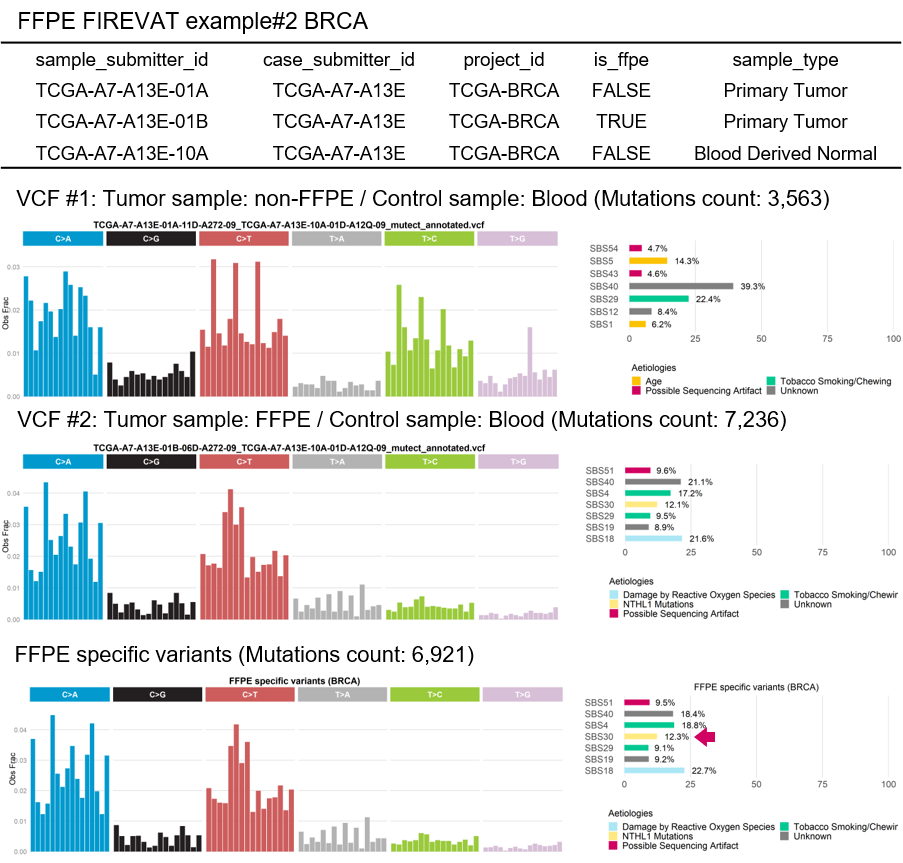


FIREVAT refinement performed comparably in a TCGA-BRCA case with FFPE sample annotation; TCGA-A7-A13E. Along with the artifact signatures SBS51, SBS52, and SBS53, the *NHTL1* mutation defective repair mechanism signature SBS30 was detected in both the original unrefined and artifact sets of mutations. It should be highlighted that the signatures SBS1 and SBS30 are predominantly comprised of C>T substitutions. In fact, the C>T peaks corresponding to these signatures have been recently shown to reflect discordant variants found in FFPE breast cancer samples compared to fresh frozen samples [9].


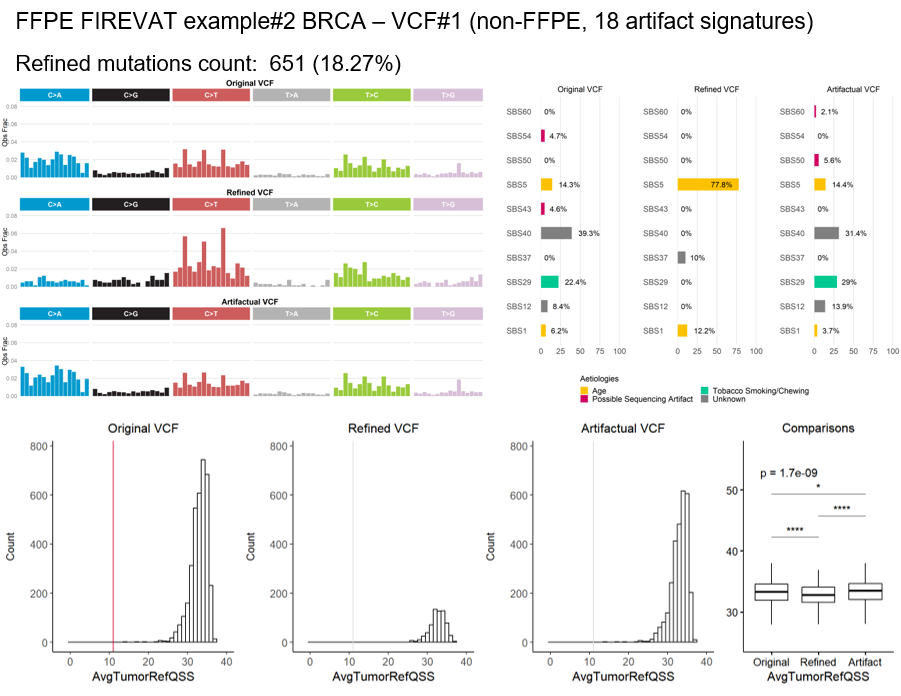


FIREVAT result of non-FFPE BRCA sample with 18 artifact signatures
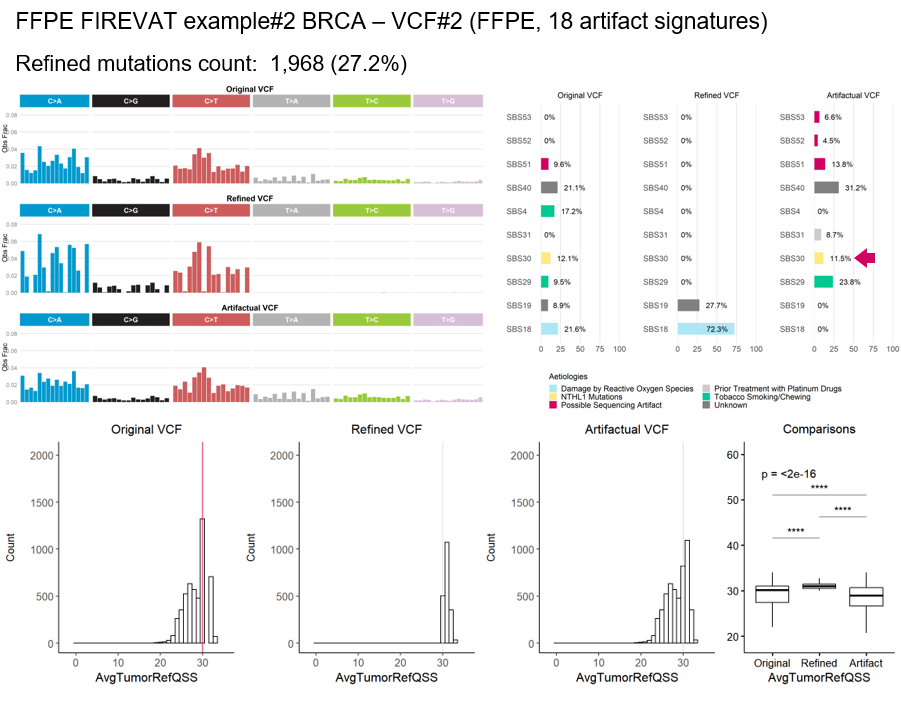


FIREVAT result of FFPE BRCA sample with 18 artifact signatures

In this case, we observed that the distribution of quality scores of both alleles in FFPE sample was shifted to the left compared to the distribution of non-FFPE sample; median 30.15 and 25.80 for the quality scores of FFPE reference allele and alternate allele, and median 33.31 and 33.00 for the quality scores of non-FFPE reference allele and alternate allele. By picking higher cut-off for reference allele quality score in the result of FFPE sample, our signature-based filtering process yielded a cosine similarity score of 0.969 between the FFPE-specific variants and the artifact variants determined.


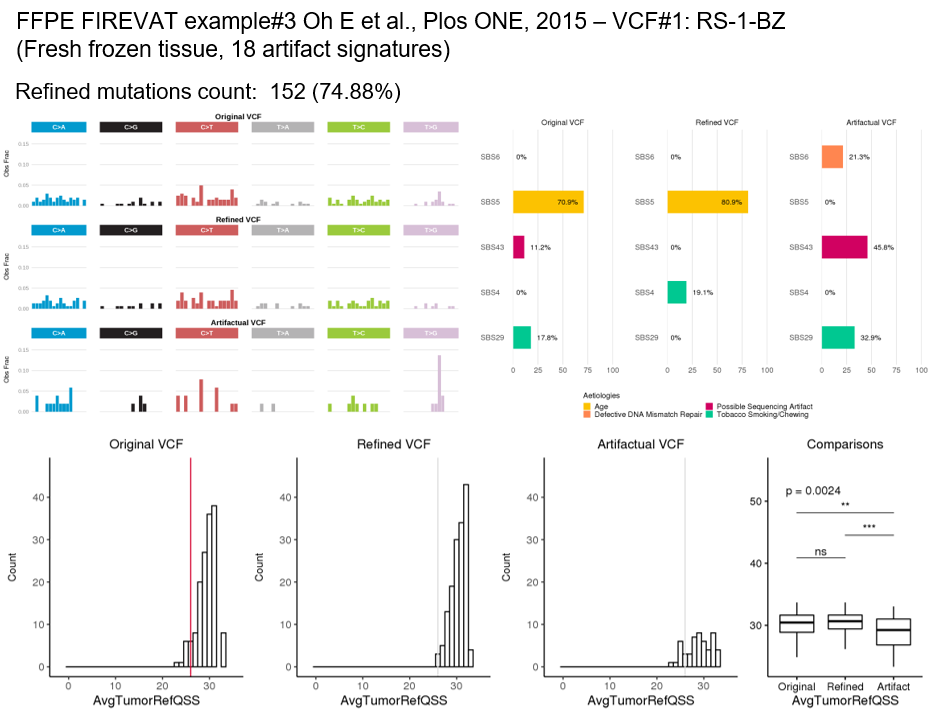


FIREVAT result of publicly reported fresh frozen sample with 18 artifact signatures
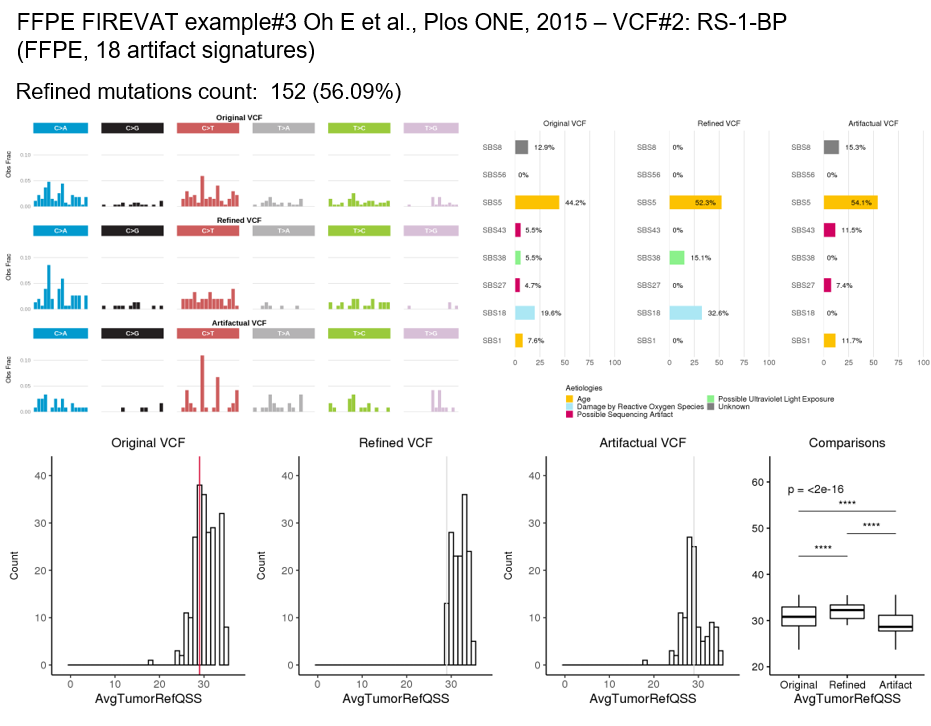


FIREVAT result of publicly reported FFPE sample with 18 artifact signatures.

Finally, to assess FIREVAT’s ability in selecting FFPE-specific artifact mutations, we analyzed a pair of FFPE and fresh frozen tumor samples (pair 1) [8]. Our findings showed that none of the artifact signatures could be identified from the refined mutations. On the other hand, the filtered-out variants decomposed into SBS1 (11.7%), SBS5 (54.1%), SBS8 (15.3%), SBS27 (7.4%), SBS43 (11.5%).

FFPE samples are frequently used both in the clinic and research settings for prolonged storage life, albeit the high potential for erroneous calling of false positive mutations. With 3 FFPE case studies above, we confirmed that the spectra of FFPE-derived variants are enriched with C>T substitutions which are shown as SBS1 or SBS30 in signature analysis results. We also found the distribution of quality scores in both alleles of FFPE samples at lower mean compared to non-FFPE samples. As we have shown, FIREVAT is able to filter out FFPE-specific artifact mutations by setting higher quality score cut-offs to FFPE samples and we expect that our tool to accommodate a more accurate analysis of FFPE-derived variant callsets.

4) Germline Variants

Here we show that FIREVAT is capable of selecting germline variants based on the presence of the germline contamination signature SBS54 and the clock-like mutational signatures SBS1 and SBS5. We demonstrate FIREVAT’s capability in accurately removing germline contamination using the MC3 MuTect callset [10]. We selected 102 samples that had at most 200,000 point mutations in the unrefined MuTect VCF file, which included all candidate variants assessed by the built-in filters in MuTect. In this callset, each variant was annotated by dbSNP and this information could be used to infer whether a given variant was likely a single-nucleotide polymorphism (SNP). FIREVAT was executed on the 102 samples and the signature analysis of artifact mutations revealed that signatures SBS1, SBS5, and SBS54 were extracted at 6.04%, 25.84%, and 8.52% on average, respectively. Next, we performed mutational signature analysis with only SBS1, SBS5, and SBS54 for the same 102 samples. Interestingly, the cosine similarity score was positively correlated with the percentage of germline variant in the artifact mutations, suggesting that the amalgamation of SBS1, SBS5, and SBS54 signatures can sufficiently explain trinucleotide spectrums of germline variants. The contribution probability of the signatures SBS1 and SBS54 were also positively correlated with the germline proportion in the artifact mutations, while SBS5 was technically negative correlated although the contribution probability remained high at an average of 84.57% with a minimum of 79.13%. Taken together, these results support the mirroring mutational patterns observed in the C>T and T>C subtypes across the 102 samples. The mirroring pattern emerges from the FIREVAT artifact mutations and when only germline variants are selected from these artifact mutations. Reconstruction of the spectrum successfully captures most of the mirrored peaks in C>T and T>C subtypes with a cosine similarity of 0.940.

**
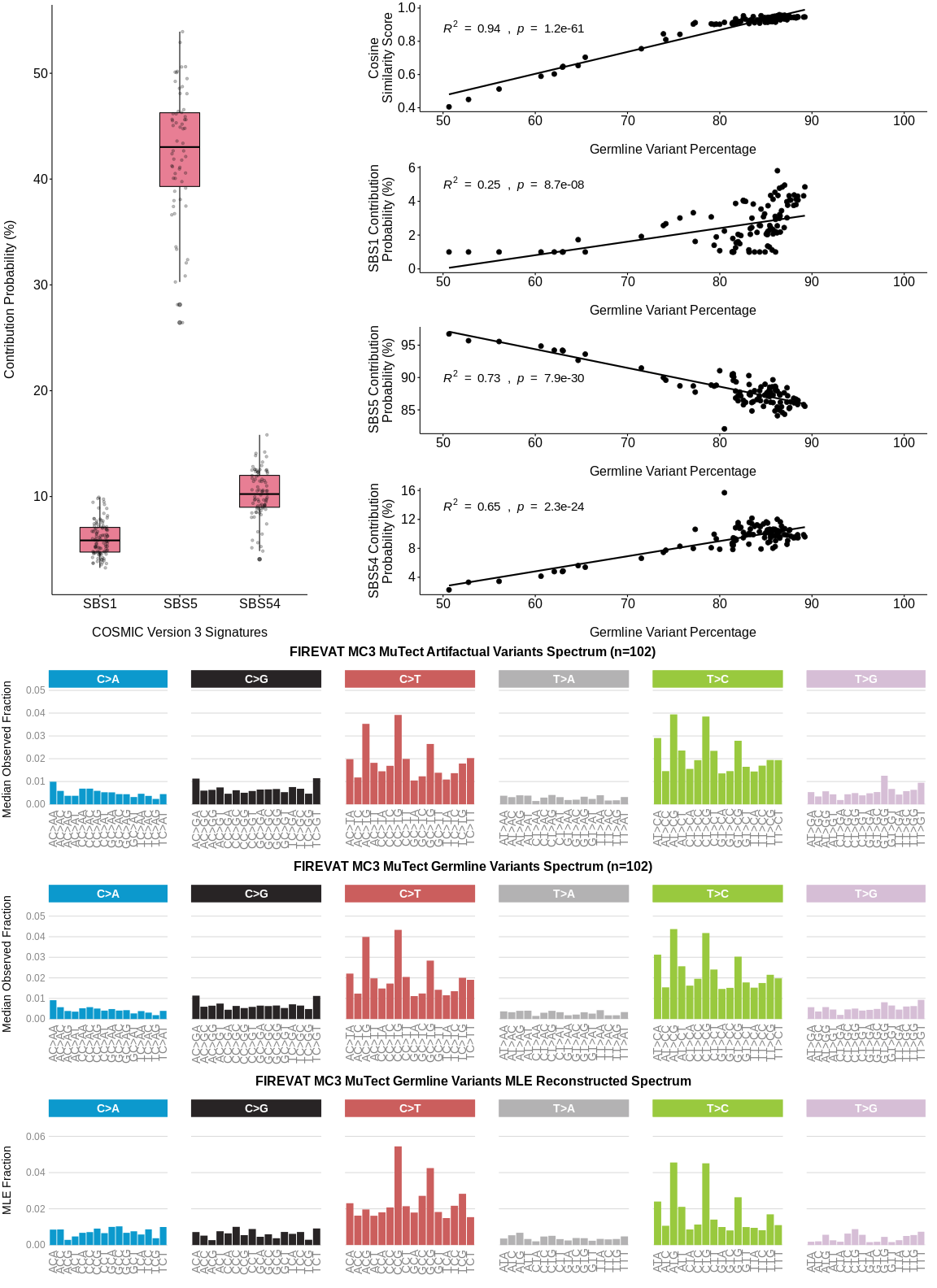
**

Accurate recapitulation of germline contamination by FIREVAT

**Note S6.** **An Example of Broader Utility of FIREVAT**


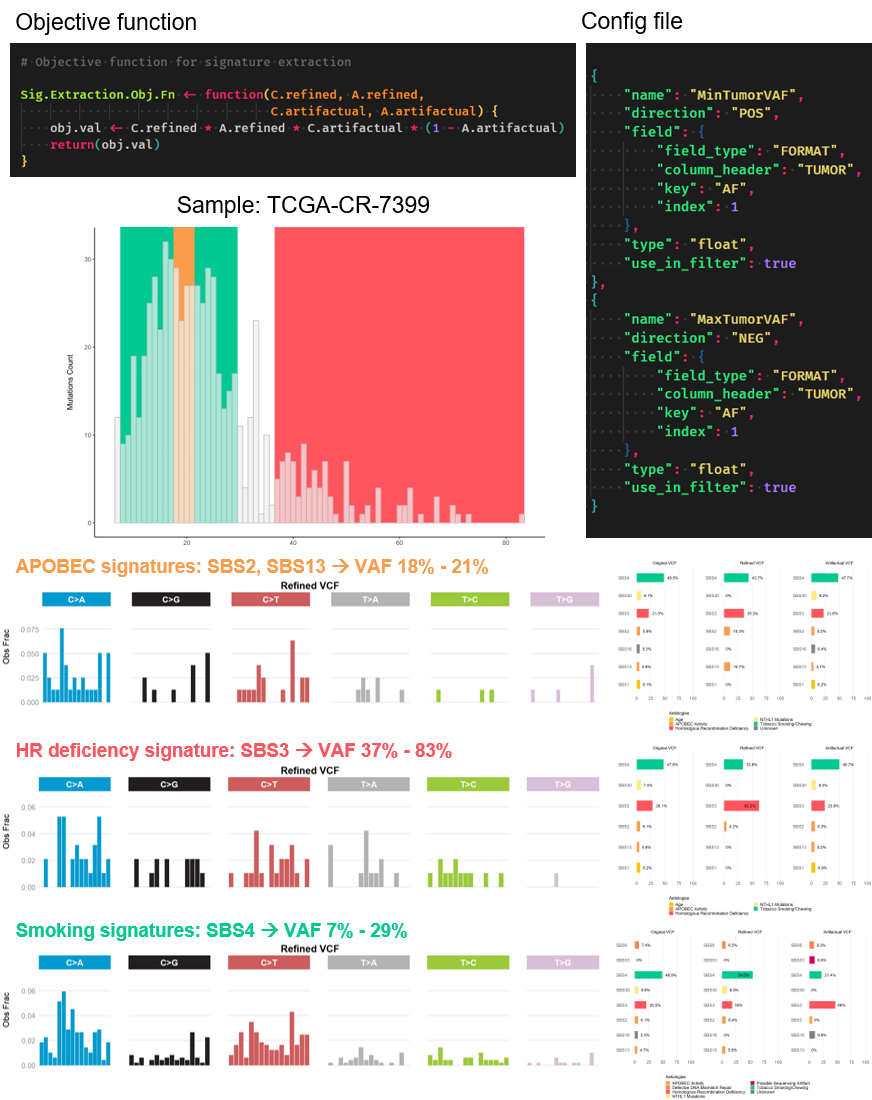


Search for VAF range where a clinical/biological signature of interest is enriched.


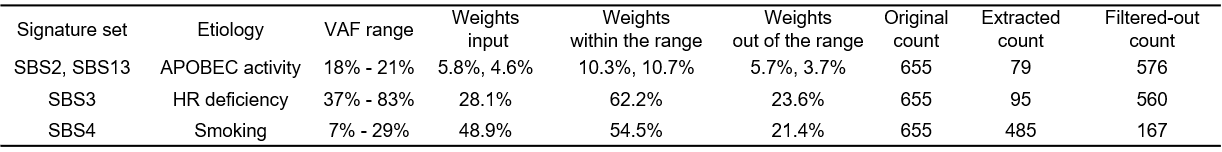


The main idea of FIREVAT is to optimize a set of conditions for a given feature space to separate variants with signatures of interest. Here, we modified FIREVAT as a signature extraction tool for clinically and biologically relevant signatures. Instead of supplying artifact signatures, we tried to find the VAF range where given mutational signatures are enriched. While mutational signature analysis paired with mutation timing have previously applied arbitrary and uniform VAF ranges as the cutoff [4]. The objective function for FIREVAT variant filtering was modified (Sig.Extraction.Obj.Fn) to minimize the weights of signatures in the variants. Thus, in this application, we maximized the weights of signatures in the variants that satisfy all of the conditions. With the FIREVAT refined mutations from the head and neck cancer case (TCGA-CR-7399) currently presented in the manuscript, FIREVAT found that APOBEC-activity related signatures (SBS2 and SBS13) are enriched in 18-21% VAF ranges

**Supplementary Methods**

**Method S1. FIREVAT Objective Functions**


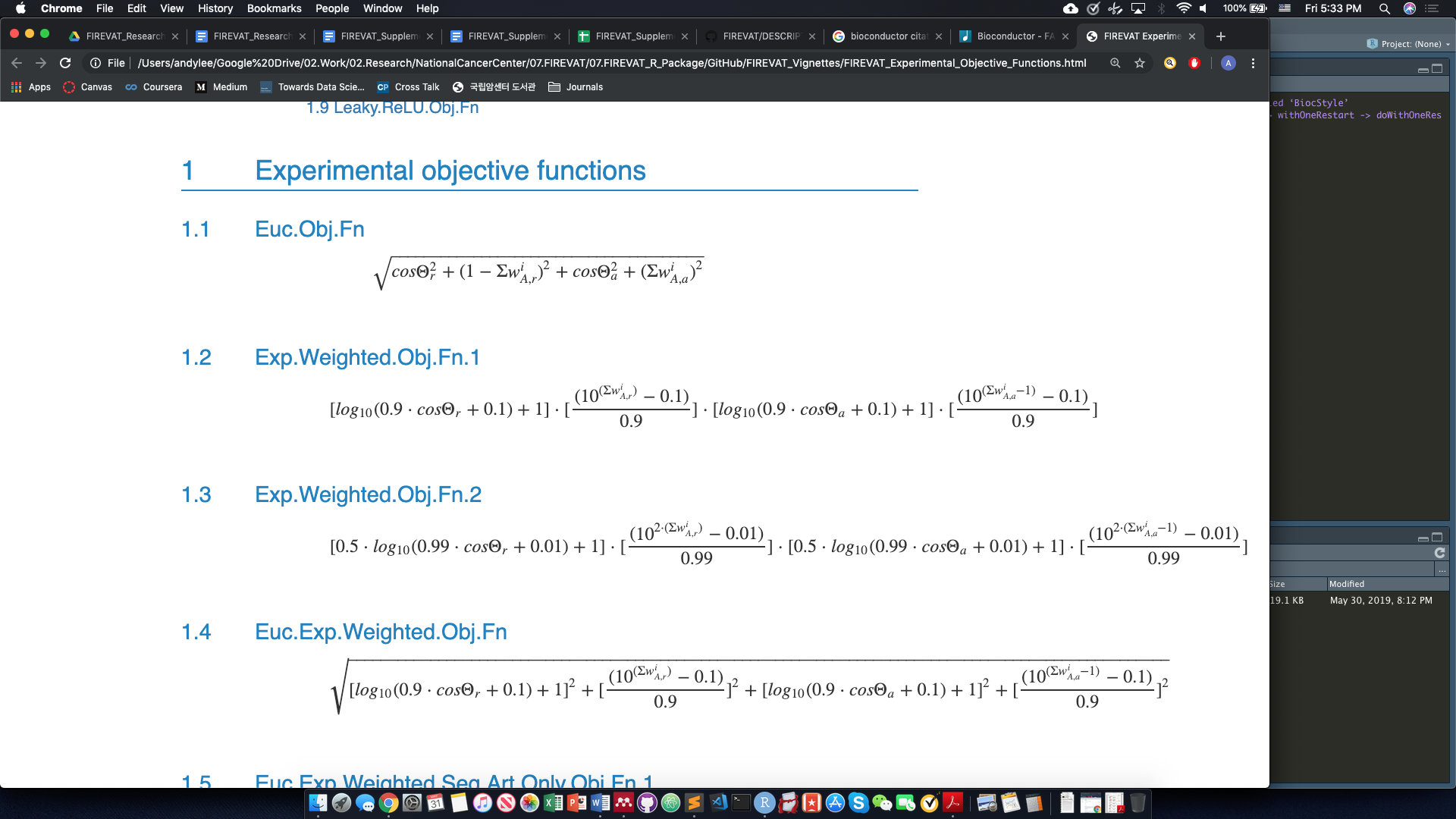


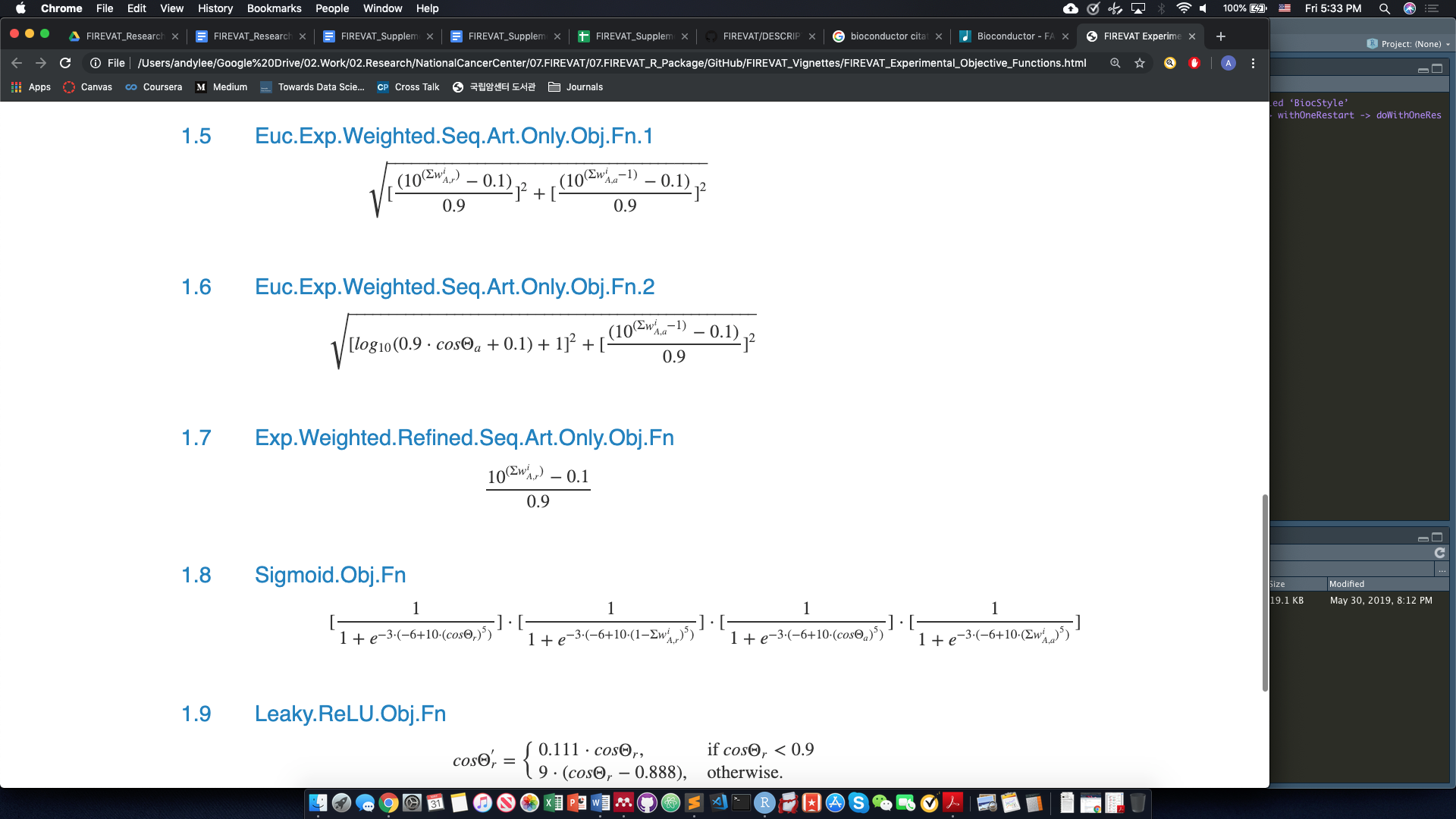


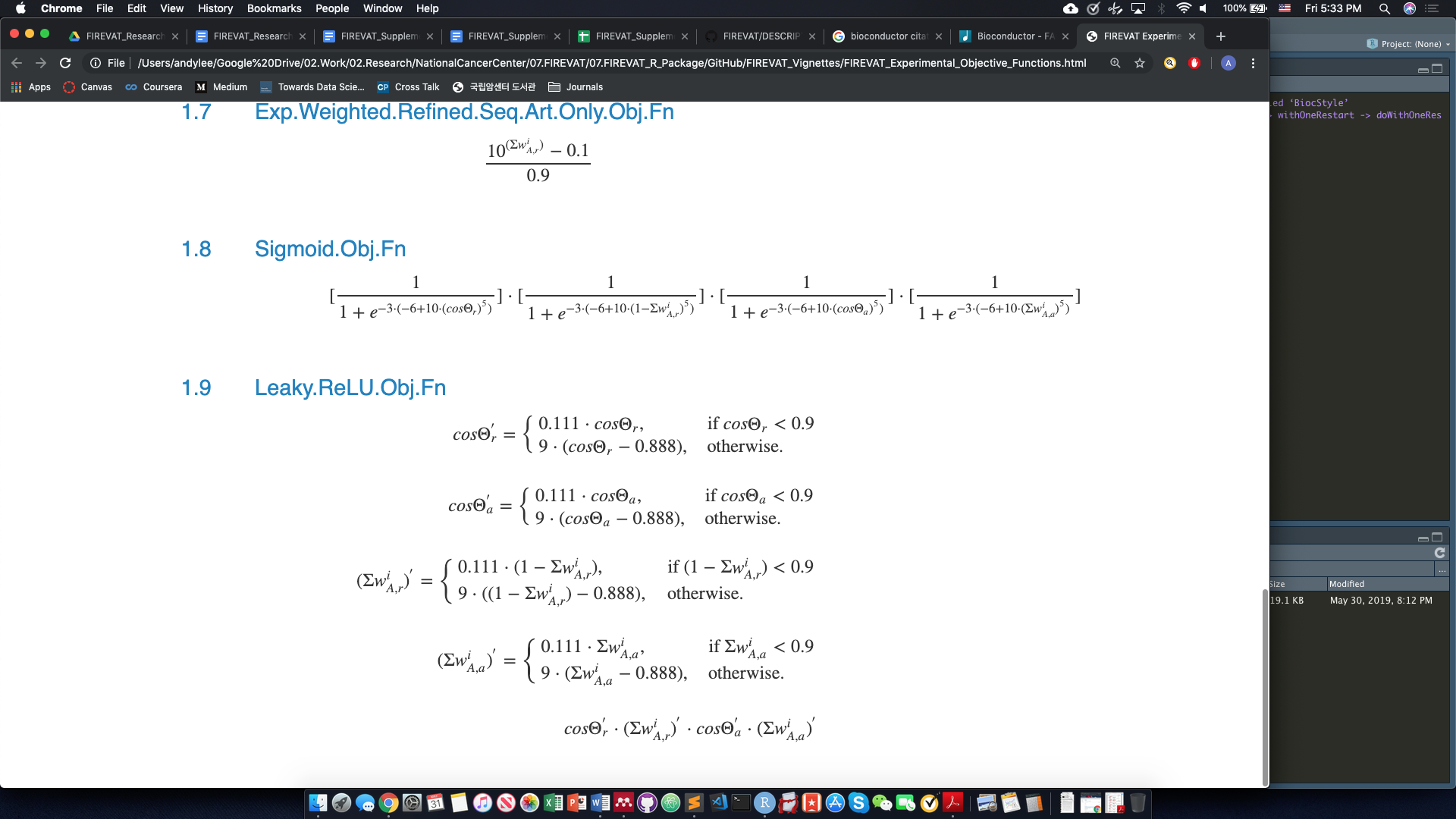


**Method S2. Variant Refinement Evaluation Methods**

To evaluate variant refinement performance, we used the following performance metrics: precision, sensitivity, F1 score, specificity, and accuracy. Here we describe the calculation of each metric.

|  |  | Ground truth | |
| --- | --- | --- | --- |
|  |  | Ground truth real | Ground truth artifact |
| Test outcome | Test outcome  real | True positive (TP) | False positive (FP) |
|  | Test outcome  artifact | False negative (FN) | True negative (TN) |

[
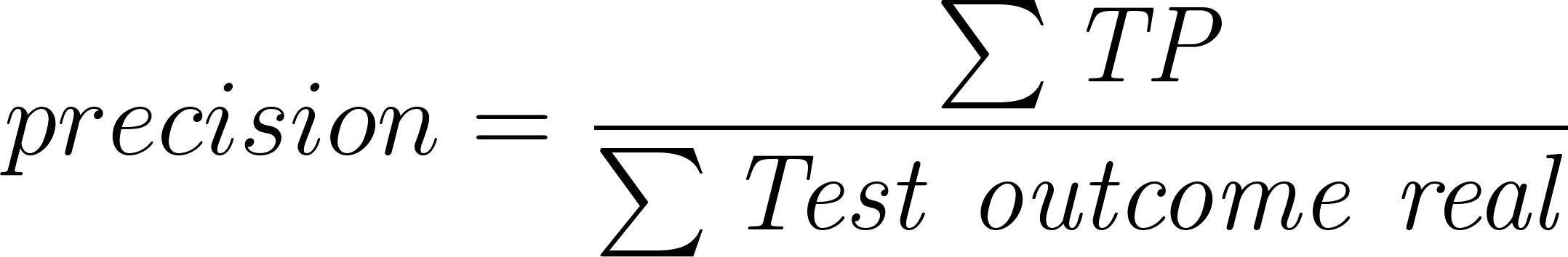
](https://www.codecogs.com/eqnedit.php?latex=%20precision%20%3D%20%5Cmathit%7B%5Cfrac%7B%5Csum%7BTP%7D%7D%7B%5Csum%7BTest%20%5C%20outcome%20%5C%20real%7D%7D%7D%20%250)

[
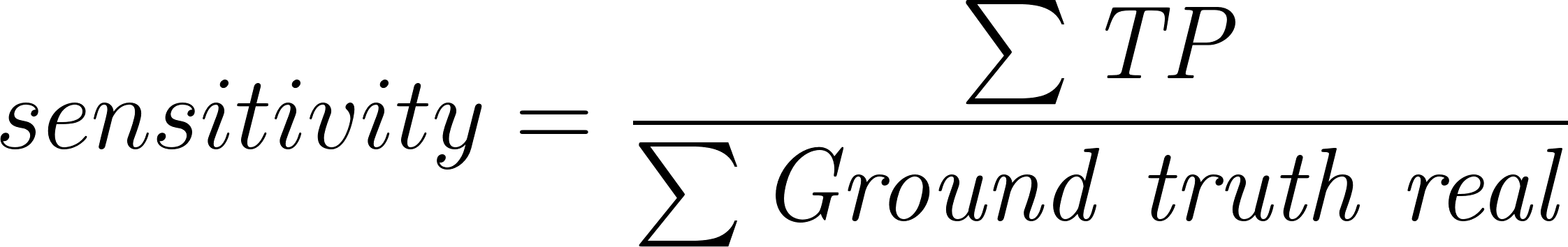
](https://www.codecogs.com/eqnedit.php?latex=%20sensitivity%20%3D%20%5Cmathit%7B%5Cfrac%7B%5Csum%7BTP%7D%7D%7B%5Csum%7BGround%20%5C%20truth%20%5C%20real%7D%7D%7D%20%250)

[
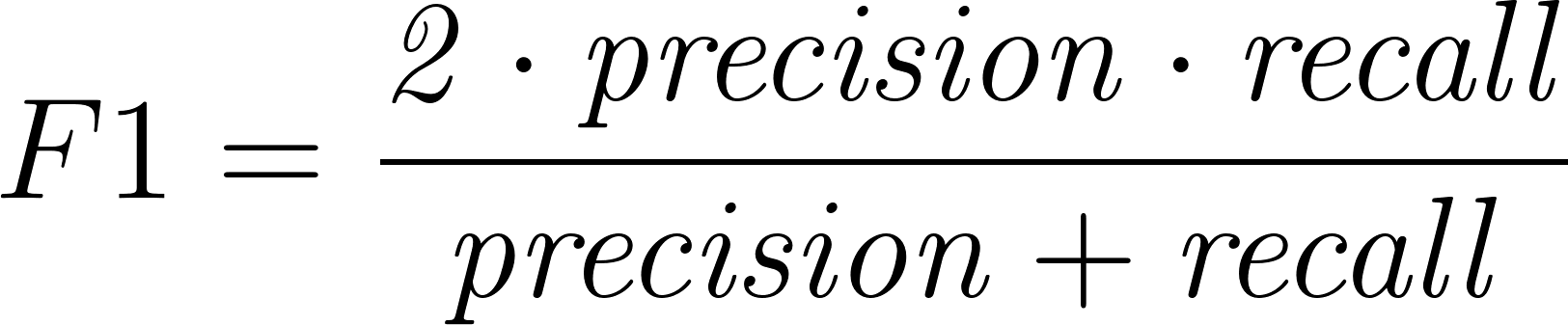
](https://www.codecogs.com/eqnedit.php?latex=%20F1%20%3D%20%5Cmathit%7B%5Cfrac%7B2%20%5Ccdot%20precision%20%5Ccdot%20recall%7D%7Bprecision%20%2B%20recall%7D%7D%20%250)

[
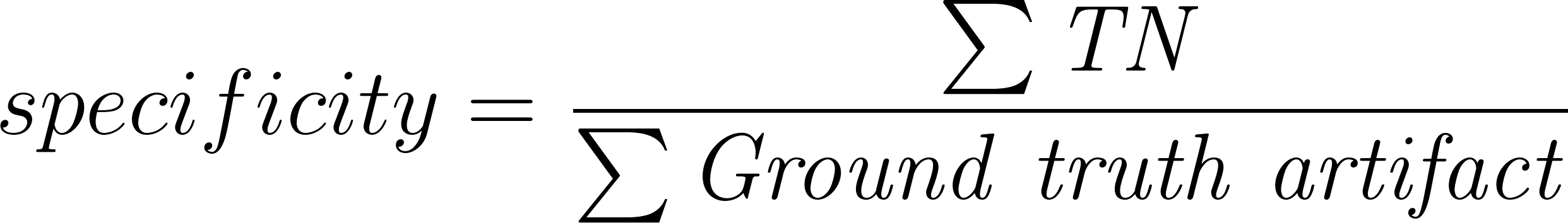
](https://www.codecogs.com/eqnedit.php?latex=%20specificity%20%3D%20%5Cmathit%7B%5Cfrac%7B%5Csum%7BTN%7D%7D%7B%5Csum%7BGround%20%5C%20truth%20%5C%20artifact%7D%7D%7D%20%250)

[
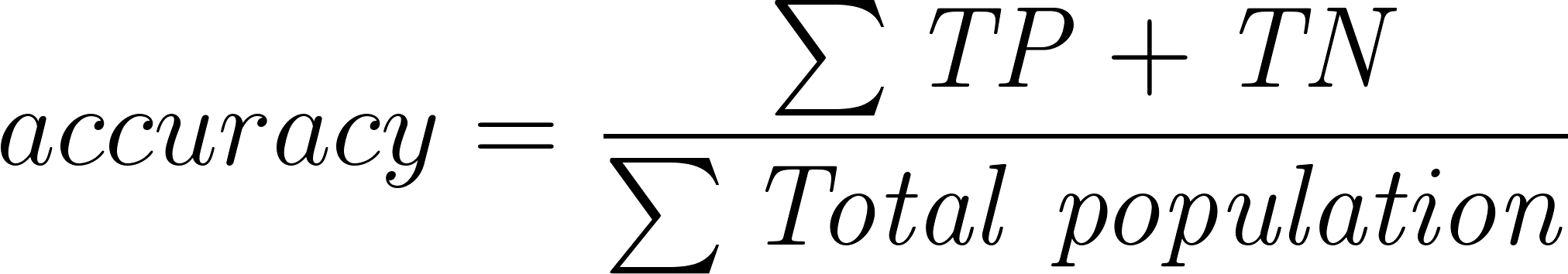
](https://www.codecogs.com/eqnedit.php?latex=%20accuracy%20%3D%20%5Cmathit%7B%5Cfrac%7B%5Csum%7BTP%20%2B%20TN%7D%7D%7B%5Csum%7BTotal%20%5C%20population%7D%7D%7D%20%250)

We compared FIREVAT variant refinement performance against three other filtering approaches suggested in MuTect, Lancet, and Varscan2. Here are the filtering cutoff values in each approach:

| **Filtering method** | **Normal Reference Allele Read Depth** | **Normal Alternative Allele Read Depth** | **Tumor Reference Allele Read Depth** | **Tumor Alternative Allele Read Depth** | **Tumor Base Quality Score** |
| --- | --- | --- | --- | --- | --- |
| Lancet (LAN-F) | 10 | 1 | 4 | 3 | - |
| MuTect (MUT-F) | - | 2 | - | - | - |
| Varscan2 (VAR-F) | 6 | - | 8 | - | 15 |

**Method S3. R sessionInfo for Validation and Downstream Analyses Scripts**

R version 3.5.1 (2018-07-02)

Platform: x86_64-redhat-linux-gnu (64-bit)

Running under: CentOS release 6.6 (Final)

Matrix products: default

BLAS: /usr/lib64/R/lib/libRblas.so

LAPACK: /usr/lib64/R/lib/libRlapack.so

locale:

[1] LC_CTYPE=en_US.UTF-8 LC_NUMERIC=C

[3] LC_TIME=en_US.UTF-8 LC_COLLATE=en_US.UTF-8

[5] LC_MONETARY=en_US.UTF-8 LC_MESSAGES=en_US.UTF-8

[7] LC_PAPER=en_US.UTF-8 LC_NAME=C

[9] LC_ADDRESS=C LC_TELEPHONE=C

[11] LC_MEASUREMENT=en_US.UTF-8 LC_IDENTIFICATION=C

attached base packages:

[1] parallel stats graphics grDevices utils datasets methods

[8] base

other attached packages:

[1] FIREVAT_0.4.2 Biobase_2.42.0 BiocGenerics_0.28.0

loaded via a namespace (and not attached):

[1] lsa_0.73.1 bitops_1.0-6

[3] matrixStats_0.54.0 MutationalPatterns_1.8.0

[5] bit64_0.9-7 doParallel_1.0.14

[7] RColorBrewer_1.1-2 progress_1.2.0

[9] httr_1.4.0 GenomeInfoDb_1.18.2

[11] SnowballC_0.5.1 tools_3.5.1

[13] R6_2.4.0 DBI_1.0.0

[15] lazyeval_0.2.1 colorspace_1.4-0

[17] withr_2.1.2 gridExtra_2.3

[19] tidyselect_0.2.5 prettyunits_1.0.2

[21] bit_1.1-14 compiler_3.5.1

[23] VennDiagram_1.6.20 extrafontdb_1.0

[25] formatR_1.5 DelayedArray_0.8.0

[27] pkgmaker_0.27 ggdendro_0.1-20

[29] rtracklayer_1.42.1 caTools_1.17.1

[31] scales_1.0.0 NMF_0.21.0

[33] stringr_1.3.0 digest_0.6.18

[35] Rsamtools_1.34.1 rmarkdown_1.11

[37] R.utils_2.8.0 XVector_0.22.0

[39] htmltools_0.3.6 pkgconfig_2.0.2

[41] extrafont_0.17 bibtex_0.4.2

[43] deconstructSigs_1.8.0 GA_3.0.2

[45] BSgenome_1.50.0 rlang_0.3.1

[47] RSQLite_2.1.1 jsonlite_1.5

[49] BiocParallel_1.16.6 R.oo_1.22.0

[51] dplyr_0.8.0.1 bedr_1.0.6

[53] VariantAnnotation_1.28.11 RCurl_1.95-4.11

[55] magrittr_1.5 GenomeInfoDbData_1.2.0

[57] futile.logger_1.4.3 Matrix_1.2-14

[59] Rcpp_1.0.0 munsell_0.5.0

[61] S4Vectors_0.20.1 R.methodsS3_1.7.1

[63] yaml_2.2.0 stringi_1.3.1

[65] MASS_7.3-51.1 SummarizedExperiment_1.12.0

[67] zlibbioc_1.28.0 plyr_1.8.4

[69] grid_3.5.1 blob_1.1.1

[71] crayon_1.3.4 lattice_0.20-38

[73] Biostrings_2.50.2 cowplot_0.9.4

[75] GenomicFeatures_1.34.3 hms_0.4.2

[77] knitr_1.20 pillar_1.3.1

[79] ggpubr_0.2 GenomicRanges_1.34.0

[81] rngtools_1.3.1 reshape2_1.4.3

[83] codetools_0.2-16 biomaRt_2.38.0

[85] stats4_3.5.1 futile.options_1.0.1

[87] XML_3.98-1.17 glue_1.3.0

[89] evaluate_0.10.1 lambda.r_1.2.3

[91] data.table_1.12.0 foreach_1.4.4

[93] testthat_2.0.1 Rttf2pt1_1.3.7

[95] gtable_0.2.0 purrr_0.3.0

[97] assertthat_0.2.0 ggplot2_3.1.0

[99] gridBase_0.4-7 xtable_1.8-3

[101] pracma_2.2.2 tibble_2.0.1

[103] iterators_1.0.9 GenomicAlignments_1.18.1

[105] AnnotationDbi_1.44.0 registry_0.5

[107] memoise_1.1.0 IRanges_2.16.0

[109] cluster_2.0.7-1

**References**

1. Ewing AD, Houlahan KE, Hu Y, Ellrott K, Caloian C, Yamaguchi TN, Bare JC, P'ng C, Waggott D, Sabelnykova VY, et al: **Combining tumor genome simulation with crowdsourcing to benchmark somatic single-nucleotide-variant detection.** *Nat Methods* 2015, **12:**623-630.

2. Costello M, Pugh TJ, Fennell TJ, Stewart C, Lichtenstein L, Meldrim JC, Fostel JL, Friedrich DC, Perrin D, Dionne D, et al: **Discovery and characterization of artifactual mutations in deep coverage targeted capture sequencing data due to oxidative DNA damage during sample preparation.** *Nucleic Acids Res* 2013, **41:**e67.

3. Alexandrov LB, Kim J, Haradhvala NJ, Huang MN, Ng AW, Boot A, Covington KR, Gordenin DA, Bergstrom E, Lopez-Bigas N, et al: **The Repertoire of Mutational Signatures in Human Cancer.** *bioRxiv* 2018.

4. Petljak M, Alexandrov LB, Brammeld JS, Price S, Wedge DC, Grossmann S, Dawson KJ, Ju YS, Iorio F, Tubio JMC, et al: **Characterizing Mutational Signatures in Human Cancer Cell Lines Reveals Episodic APOBEC Mutagenesis.** *Cell* 2019, **176:**1282-1294 e1220.

5. Alioto TS, Buchhalter I, Derdak S, Hutter B, Eldridge MD, Hovig E, Heisler LE, Beck TA, Simpson JT, Tonon L, et al: **A comprehensive assessment of somatic mutation detection in cancer using whole-genome sequencing.** *Nat Commun* 2015, **6:**10001.

6. Shi W, Ng CKY, Lim RS, Jiang T, Kumar S, Li X, Wali VB, Piscuoglio S, Gerstein MB, Chagpar AB, et al: **Reliability of Whole-Exome Sequencing for Assessing Intratumor Genetic Heterogeneity.** *Cell Rep* 2018, **25:**1446-1457.

7. Griffith M, Miller CA, Griffith OL, Krysiak K, Skidmore ZL, Ramu A, Walker JR, Dang HX, Trani L, Larson DE, et al: **Optimizing cancer genome sequencing and analysis.** *Cell Syst* 2015, **1:**210-223.

8. Oh E, Choi YL, Kwon MJ, Kim RN, Kim YJ, Song JY, Jung KS, Shin YK: **Comparison of Accuracy of Whole-Exome Sequencing with Formalin-Fixed Paraffin-Embedded and Fresh Frozen Tissue Samples.** *PLoS One* 2015, **10:**e0144162.

9. Bhagwate AV, Liu Y, Winham SJ, McDonough SJ, Stallings-Mann ML, Heinzen EP, Davila JI, Vierkant RA, Hoskin TL, Frost M, et al: **Bioinformatics and DNA-extraction strategies to reliably detect genetic variants from FFPE breast tissue samples.** *BMC Genomics* 2019, **20:**689.

10. Ellrott K, Bailey MH, Saksena G, Covington KR, Kandoth C, Stewart C, Hess J, Ma S, Chiotti KE, McLellan M, et al: **Scalable Open Science Approach for Mutation Calling of Tumor Exomes Using Multiple Genomic Pipelines.** *Cell Syst* 2018, **6:**271-281 e277.
