# Supplementary material for: FIREVAT: finding reliable variants without artifacts in human cancer samples using etiologically relevant mutational signatures
Source: Genome Med. 2019 Dec 17;11:81. doi: 10.1186/s13073-019-0695-x (PMC6916105; doi:10.1186/s13073-019-0695-x)
Supplement: Supplementary file 2 — Additional file 2. Figure S1 to S4. Artifactual signatures in the TCGA (MC3) dataset, Figure S5. Spectrums of mutational signatures related to sequencing artifact, Figure S6. Hierarchical clustering result of the mutational signatures, Figure S7. Benchmark test results of objective functions and GA parameters, Figure S8 to S10. Convergence of filter parameters in the FIREVAT refinement, Figure S11 to S13. Correlation between FIREVAT performance and the artifactual signature weights, Figure S14 to S17. Scatterplots of performance evaluation metrics on the MC3 validation dataset from the FIREVAT and other post variant-caller filtering methods, Figure S18 and S19. FIREVAT refinement on the multi-region whole exome sequencing data of breast cancer cases, Figure S20. Before and after FIREVAT refinement on the TCGA-HNSC, Figure S21. Before and after FIREVAT refinement on the TCGA platinum therapy responder and non-responder samples, Figure S22. FIREVAT results of TCGA-FP-8211, Figure S23. Characteristics of artifactual variants in TCGA-BRCA, TCGA-GBM, TCGA-KIRC, and TCGA-PAAD. [file 13073_2019_695_MOESM2_ESM.docx]

**SUPPLEMENTARY FIGURES**

**FIREVAT: finding reliable variants without artifacts in human cancer samples using etiologically relevant mutational signatures**

**Hyunbin Kim^1,*^, Andy Jinseok Lee^1,*^, Jongkeun Lee^1^, Hyonho Chun^2^, Young Seok Ju^3^, and Dongwan Hong^1,#^**

**Affiliations**

1. Bioinformatics Analysis Team, National Cancer Center, Goyang 10408, Republic of Korea
2. Department of Mathematics and Statistics, Boston University, Boston, MA 02215, USA
3. Graduate School of Medical Science and Engineering, Korea Advanced Institute of Science

and Technology, Daejeon 34141, Republic of Korea

* Co-first authors with equal contribution

# Correspondence

**Address for correspondence**

Dongwan Hong, PhD

Chief Researcher

Bioinformatics Analysis Team, National Cancer Center,

323 Ilsan-ro, Ilsandong-gu, Goyang-si, Gyeonggi-do 10408, Republic of Korea

E-mail: dwhong@ncc.re.kr

Tel: +82-31-920-2433

Fax: +82-31-920-2006

**Table of Contents**

**Supplementary Figures**

**Fig. S1.** Artifactual signatures in the TCGA (MC3) dataset (30 cohorts) based on
MuTect callset (n = 7,490).

**Fig. S2.** Artifactual signatures in the TCGA (MC3) dataset (33 cohorts) based on
Muse callset (n = 10,809).

**Fig. S3.** Artifactual signatures in the TCGA (MC3) dataset (33 cohorts) based on
SomaticSniper callset (n = 9,847).

**Fig. S4.** Artifactual signatures in the TCGA (MC3) dataset (33 cohorts) based on
Varscan callset (n = 10,982).

**Fig. S5.** Trinucleotide spectrums of mutational signatures related to sequencing artifact in the COSMIC mutational signatures (version 3).

**Fig. S6.** Hierarchical clustering (based on Pearson correlation distance) results of the
COSMIC mutational signatures (version 3).

**Fig. S7.** Benchmark test results on FIREVAT objective functions and genetic algorithm (GA)
input parameters.

**Fig. S8.** Convergence of filter parameters in the FIREVAT refinement results of the MC3

validation dataset (MuTect callset).

**Fig. S9.** Convergence of filter parameters in the FIREVAT refinement results of the MC3

validation dataset (Muse callset).

**Fig. S10.** Convergence of filter parameters in the FIREVAT refinement results of the MC3

validation dataset (Varscan callset).

**Fig. S11.** Correlation between FIREVAT performance and artifactual signature weights in the MC3

validation dataset (MuTect callset).

**Fig. S12.** Correlation between FIREVAT performance and artifactual signature weights in the MC3

validation dataset (Muse callset)**.**

**Fig. S13.** Correlation between FIREVAT performance and artifactual signature weights in the MC3

validation dataset (Varscan callset)**.**

**Fig. S14.** Scatterplots of various performance evaluation metrics on the MC3 validation dataset

(MuTect callset) from the FIREVAT refinement results and other post variant-caller

filtering methods.

**Fig. S15.** Scatterplots of various performance evaluation metrics on the MC3 validation dataset

(Muse callset) from the FIREVAT refinement results and other post variant-caller filtering

methods.

**Fig. S16.** Scatterplots of various performance evaluation metrics on the MC3 validation dataset

(Varscan callset) from the FIREVAT refinement results and other post variant-caller filtering methods.

**Fig. S17.** Scatterplots of various performance evaluation metrics on the MC3 validation dataset (all

callsets combined) from the FIREVAT refinement results and other post variant-caller

filtering methods.

**Fig. S18.** Before (unrefined) and after (refined) FIREVAT refinement on the multi-region whole-

exome sequencing data of breast cancer cases (technical replicates).

**Fig. S19.** UpSet plots of before (orange) and after (green) FIREVAT refinement on the multi-region

whole-exome sequencing data of breast cancer cases (biological replicates).

**Fig S20.** Before and after FIREVAT refinement on the TCGA-HNSC dataset using COSMIC

mutational signatures versions 2 (30 signatures) and 3 (65 signatures).

**Fig. S21.** The HR deficiency signature SBS3 weight before and after FIREVAT variant refinement

in the TCGA platinum therapy responder and non-responder samples (n = 79).

**Fig. S22.** FIREVAT results of the TCGA stomach cancer case (TCGA-FP-8211) with complete

clinical response to a regimen of platinum therapy (oxaliplatin).

**Fig. S23.** Characteristics of artifactual variants identified by FIREVAT in TCGA-BRCA, TCGA-

GBM, TCGA-KIRC, and TCGA-PAAD cohorts.

**Supplementary Figures**


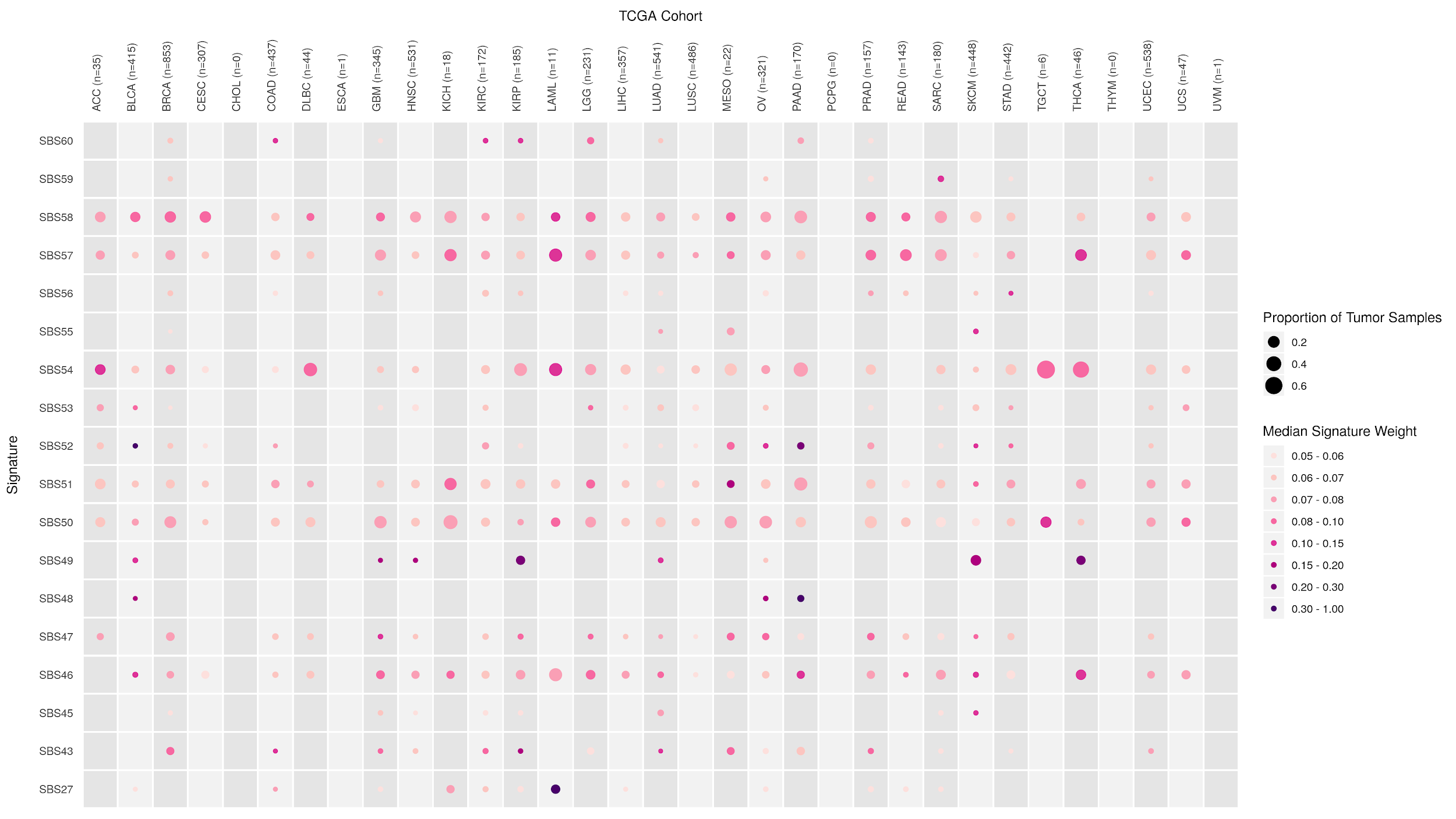


**Fig. S1.** Artifactual signatures in the TCGA (MC3) dataset (30 cohorts) based on MuTect callset (n = 7,490). Only samples with cosine similarity score 0.9 or higher were included in the analysis. For each sample, a given mutational signature was only considered if its weight was 0.05 or higher.


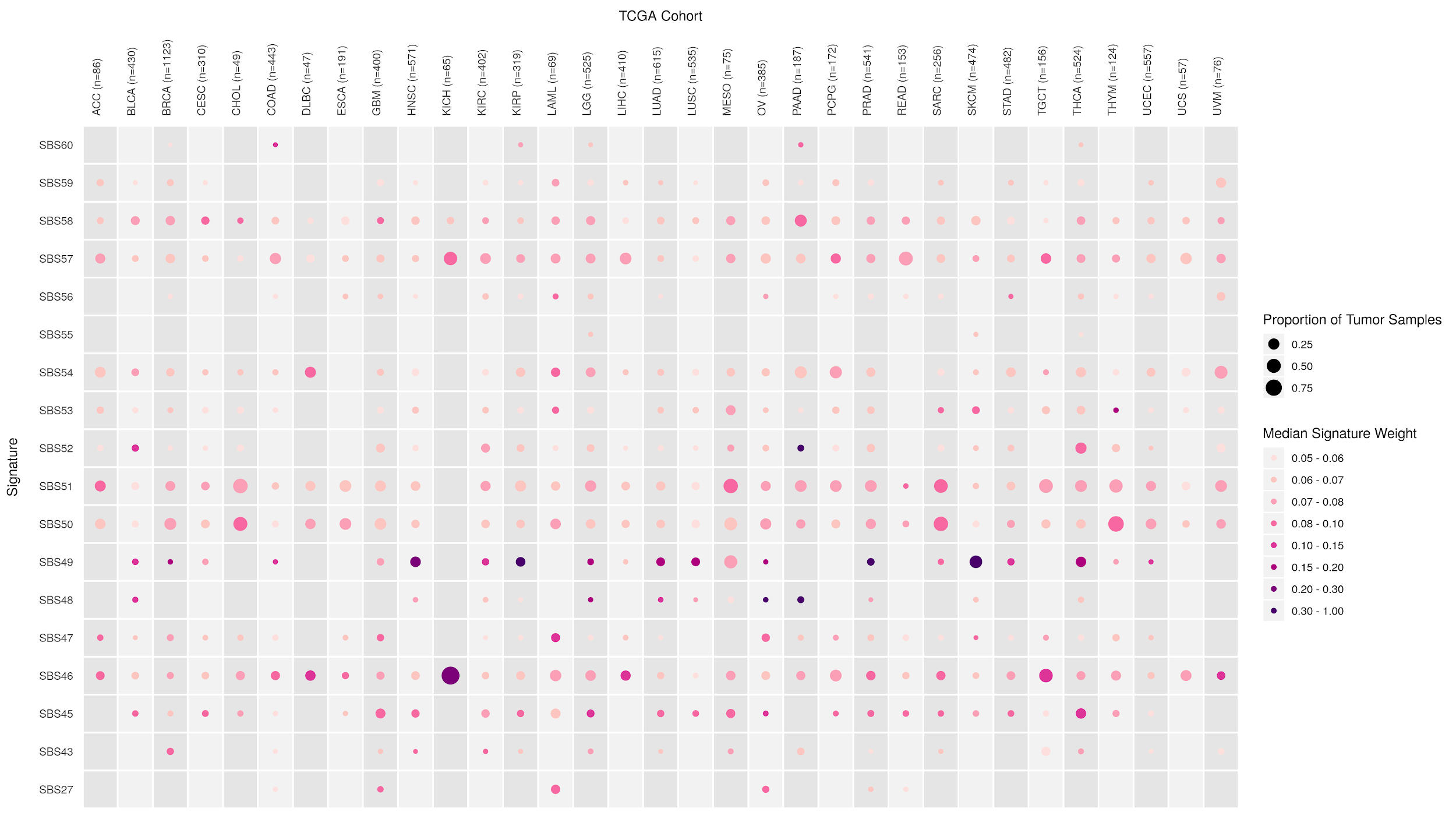


**Fig. S2.** Artifactual signatures in the TCGA (MC3) dataset (33 cohorts) based on Muse callset (n = 10,809). Only samples with cosine similarity score 0.9 or higher were included in the analysis. For each sample, a given mutational signature was only considered if its weight was 0.05 or higher.

**
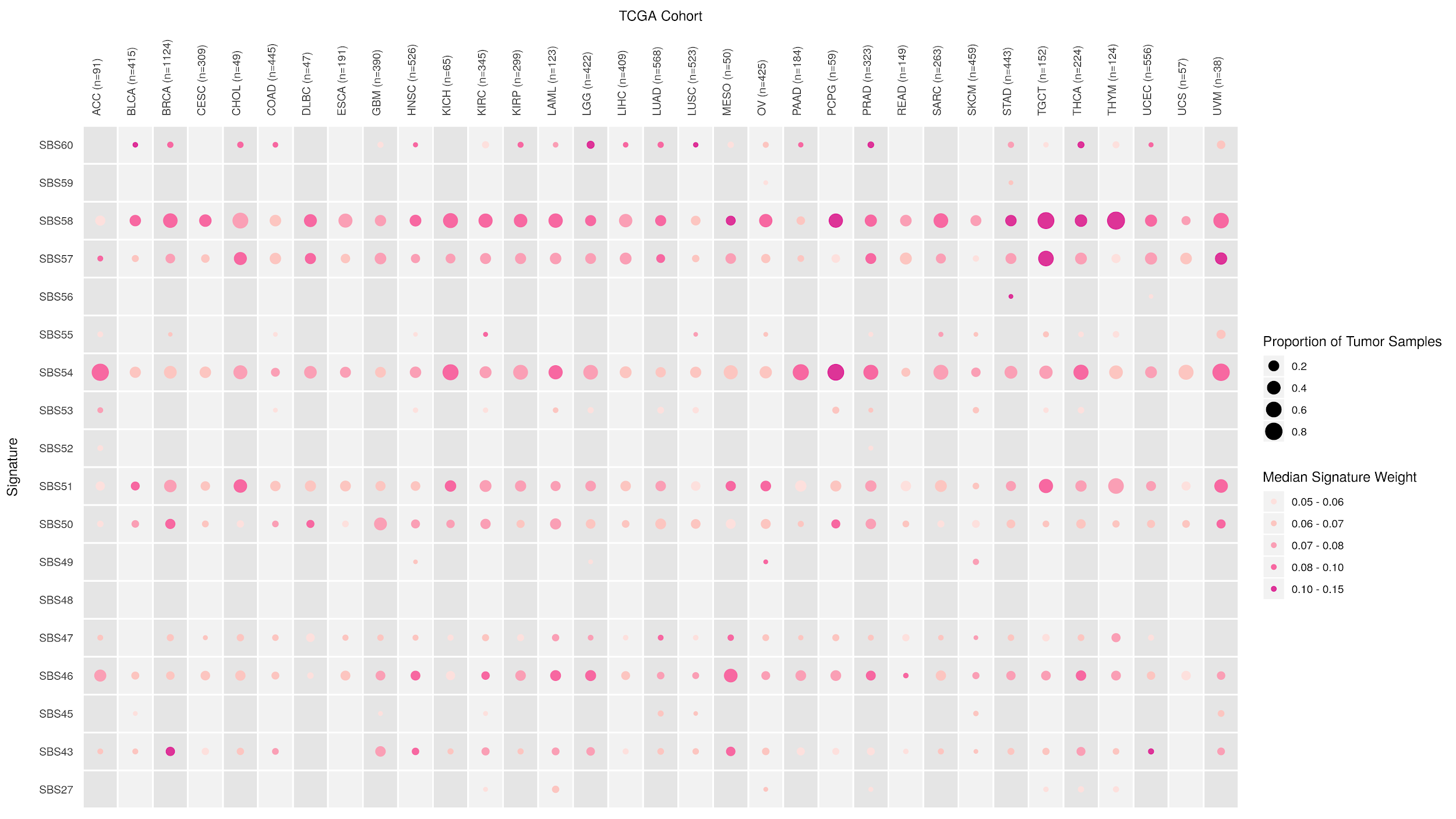
**

**Fig. S3.** Artifactual signatures in the TCGA (MC3) dataset (33 cohorts) based on SomaticSniper callset (n = 9,847). Only samples with cosine similarity score 0.9 or higher were included in the analysis. For each sample, a given mutational signature was only considered if its weight was 0.05 or higher.


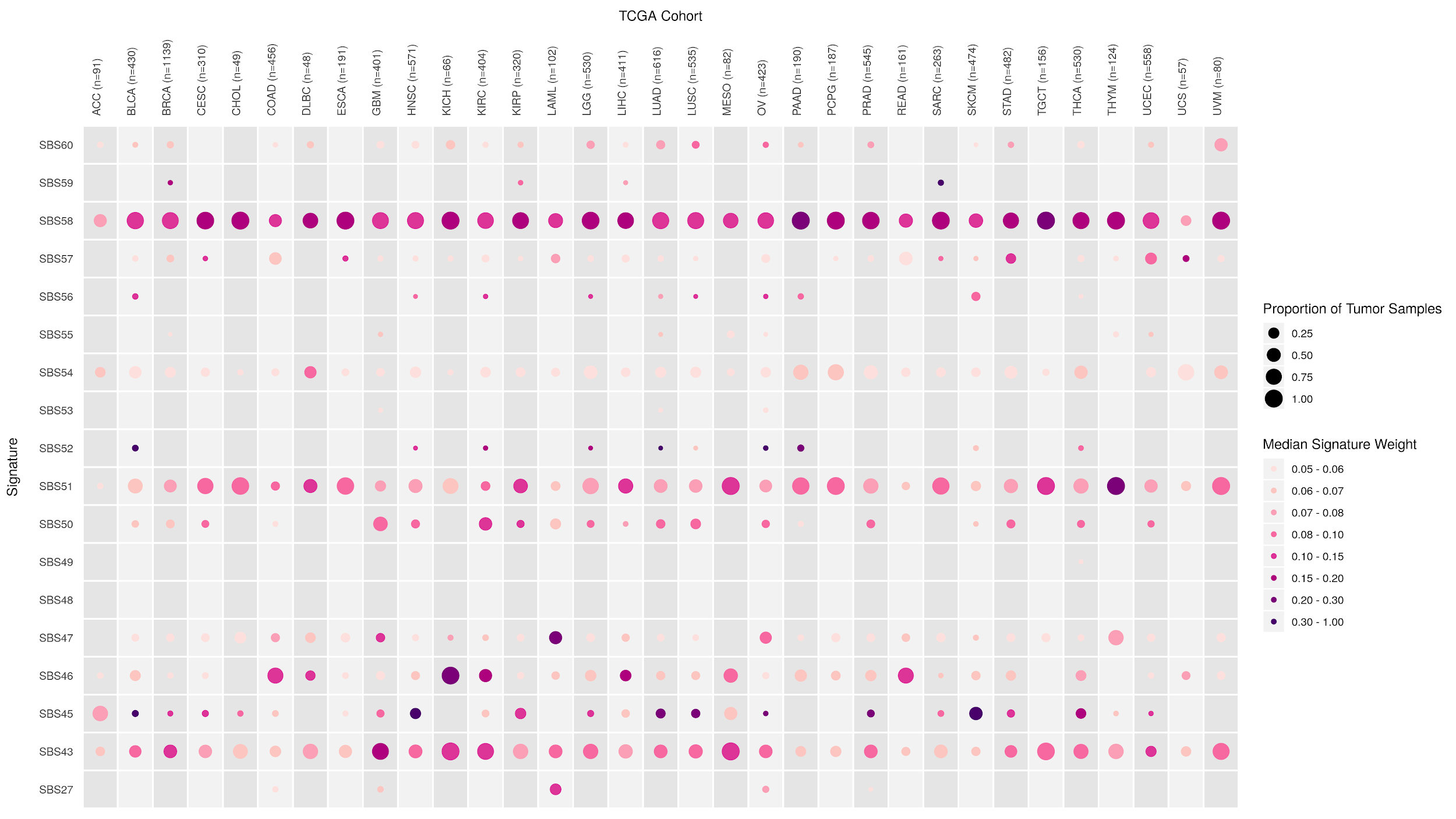


**Fig. S4.** Artifactual signatures in the TCGA (MC3) dataset (33 cohorts) based on Varscan callset (n = 10,982). Only samples with cosine similarity score 0.9 or higher were included in the analysis. For each sample, a given mutational signature was only considered if its weight was 0.05 or higher.


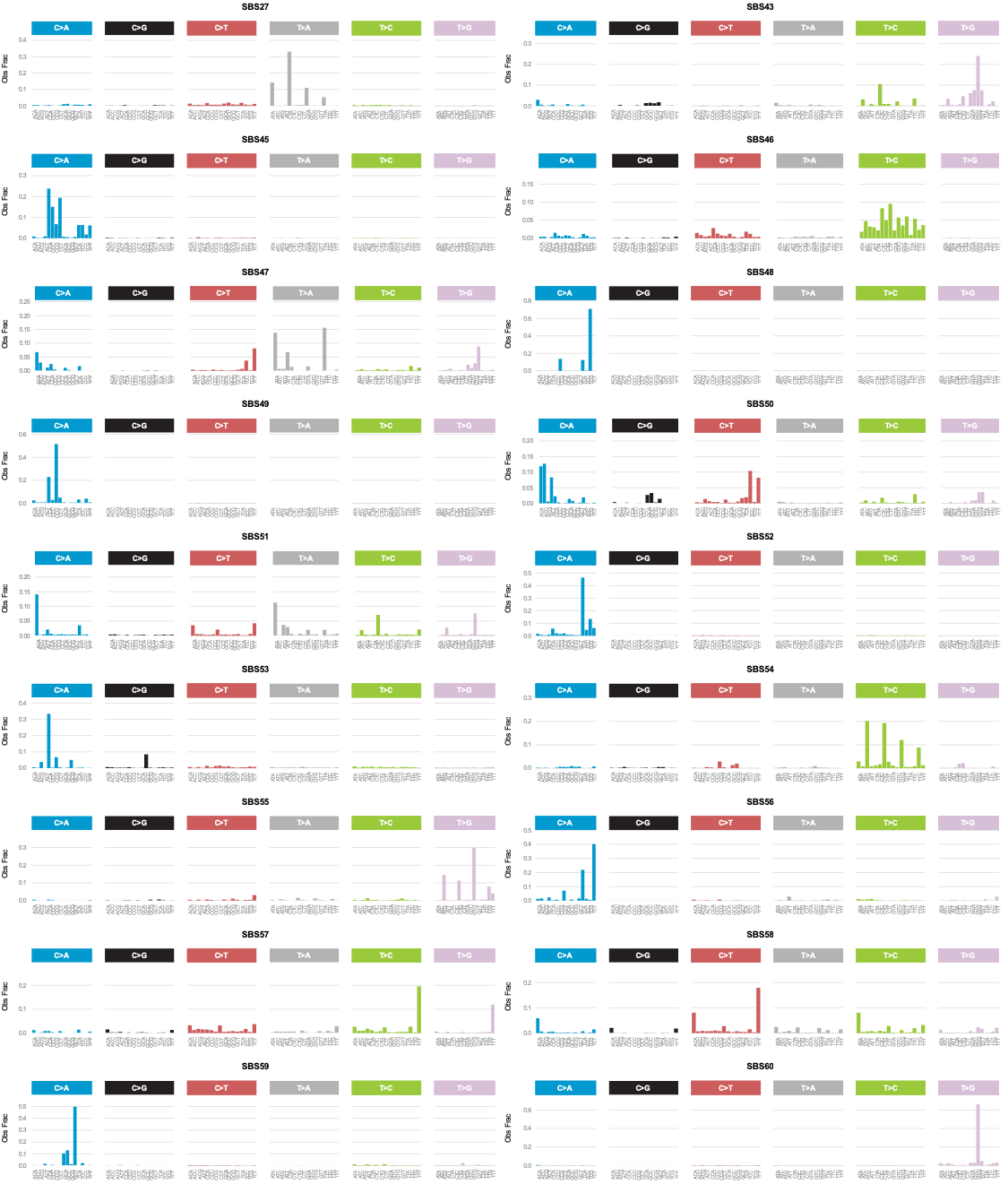


**Fig. S5.** Trinucleotide spectrums of mutational signatures related to sequencing artifact in the COSMIC mutational signatures (version 3).


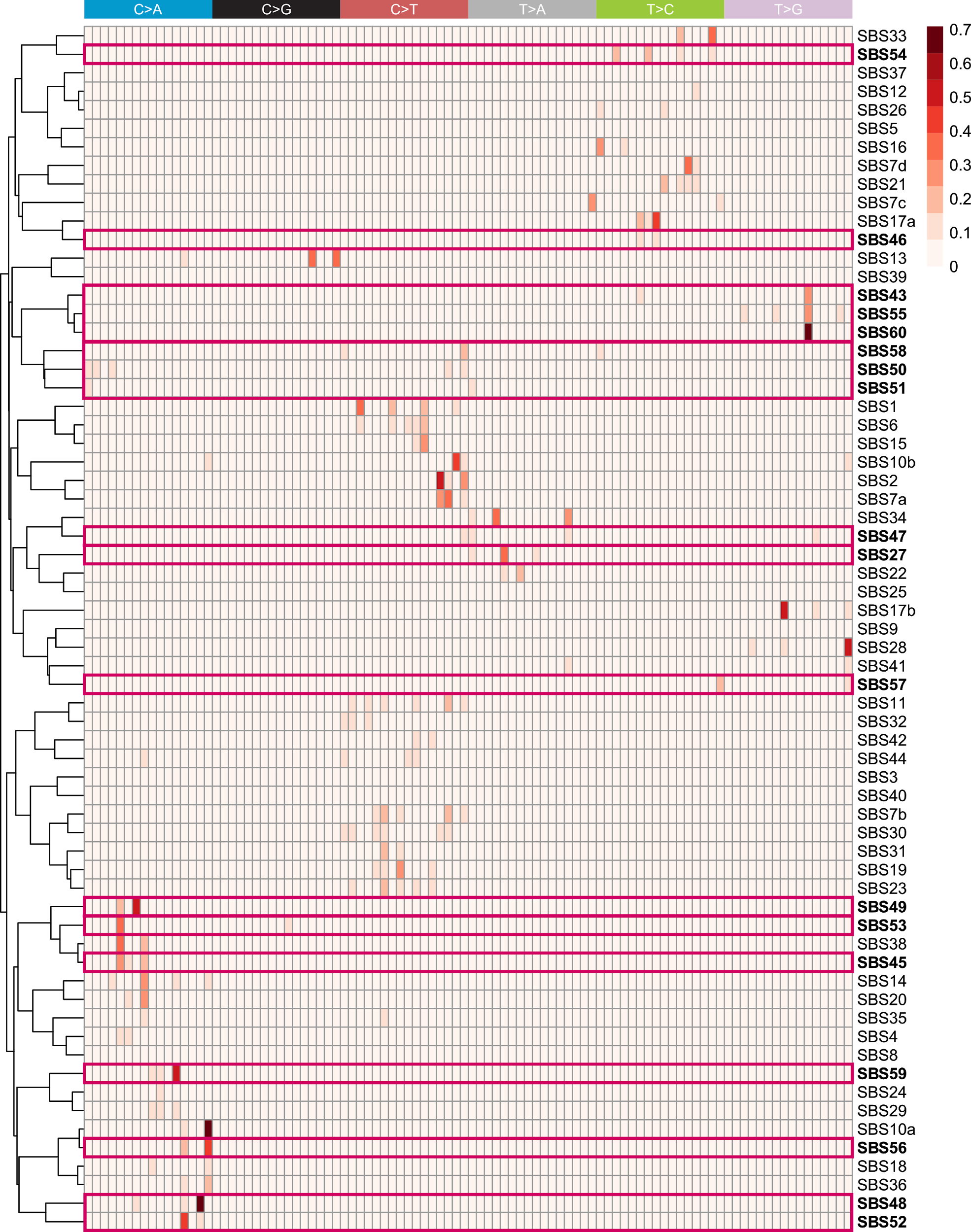


**Fig. S6.** Hierarchical clustering (based on Pearson correlation distance) results of the COSMIC mutational signatures (version 3). The heatmap gradient represents the contribution weight of the corresponding trinucleotide substitution subtype. Signatures related to sequencing artifact are highlighted in red boxes.


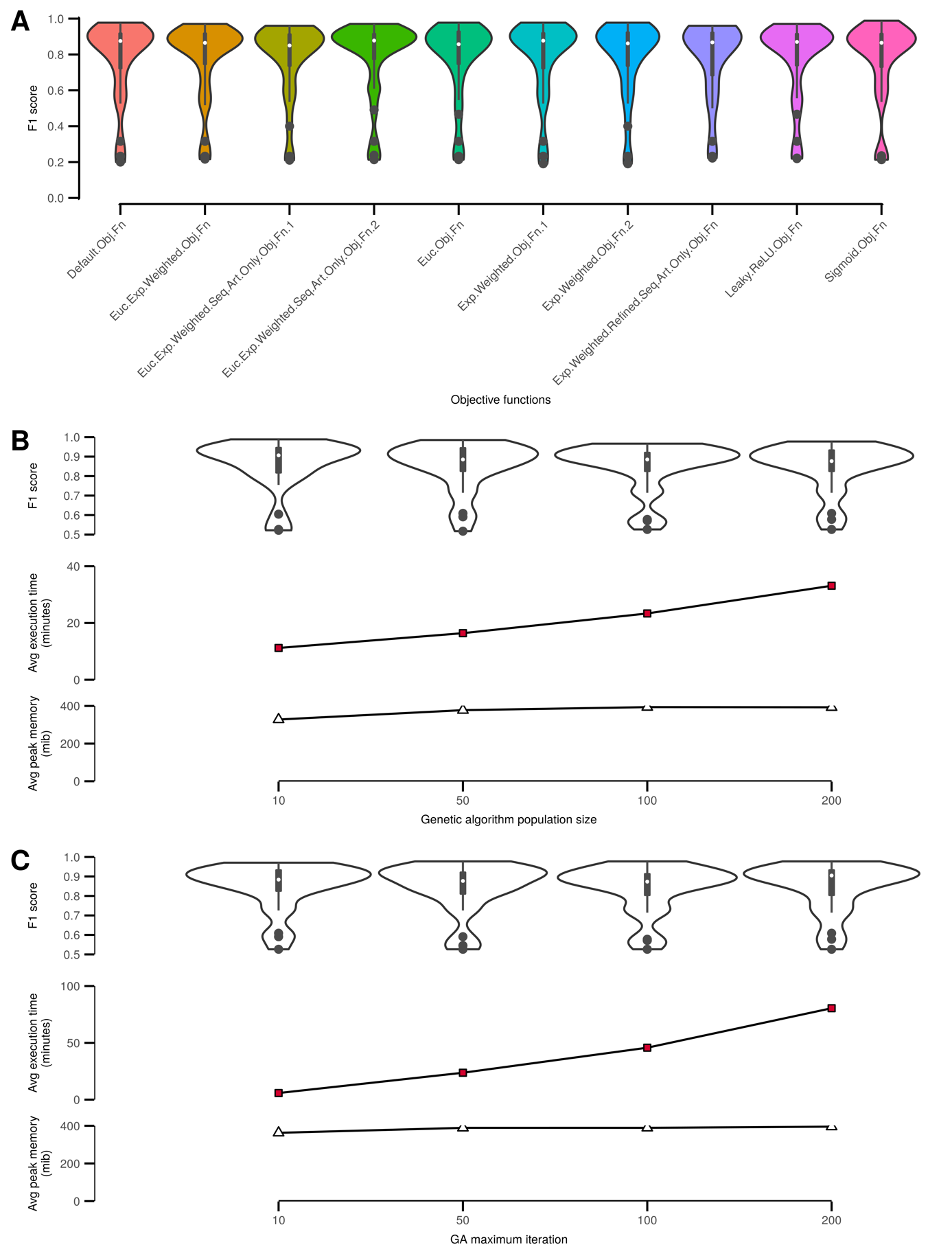


**Fig. S7.** Benchmark test results on FIREVAT objective functions and genetic algorithm (GA) input parameters. A total of 28 MC3 samples (24 HNSC samples and 4 SKCM samples) were used to perform the benchmark tests. **(A)** F1 scores of various objective functions. **(B)** F1 scores, average execution time (minutes) and average peak memory usage (mib) by the GA population sizes. **(C)** F1 scores, average execution time (minutes) and average peak memory usage (mib) by the GA maximum iterations.


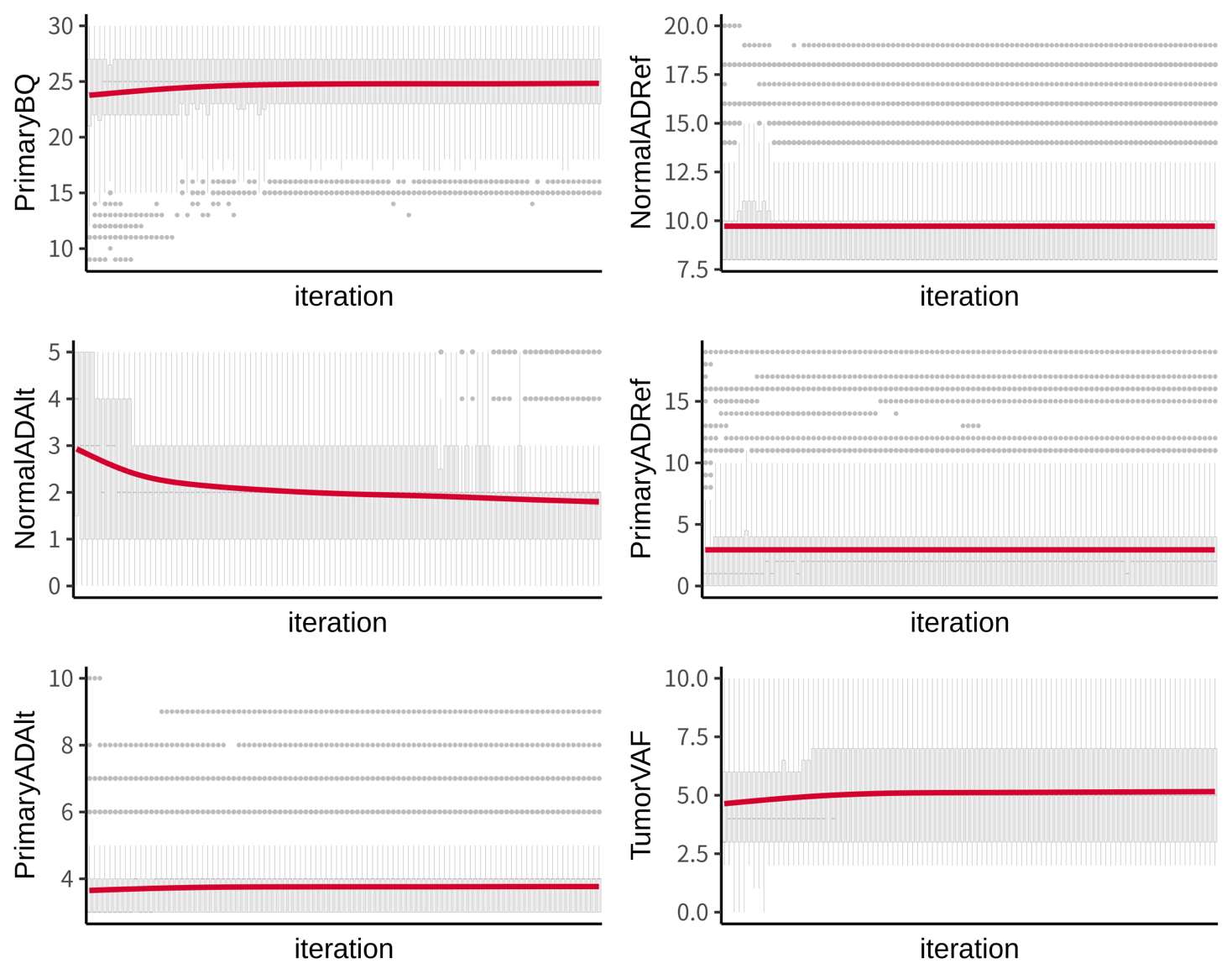


**Fig. S8.** Convergence of filter parameters in the FIREVAT refinement results of the MC3 validation dataset (MuTect callset). The red lines represent the regression of the fittest values in each iteration, drawn using generative additive model (gam in R). PrimaryBQ is the average base quality of the tumor sample reads. NormalADRef is the number of reads supporting the reference allele in the normal sample. NormalADAlt is the number of reads supporting the alternate allele in the normal sample. PrimaryADRef is the number of reads supporting the reference allele in the tumor sample. PrimaryADAlt is the number of reads supporting the alternate allele in the tumor sample. TumorVAF is the variant allele fraction in the tumor sample in percentage.


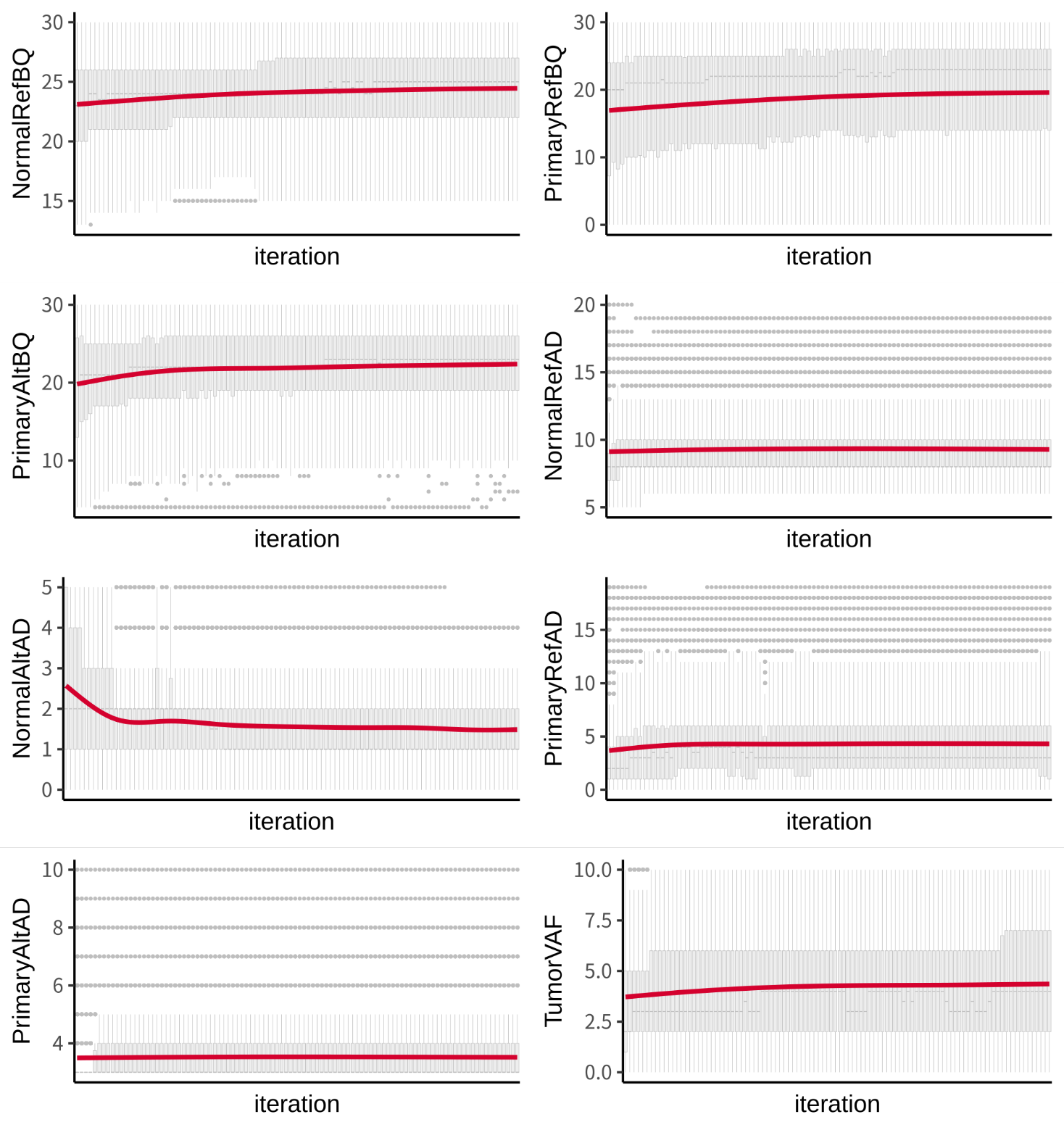


**Fig. S9.** Convergence of filter parameters in the FIREVAT refinement results of the MC3 validation dataset (Muse callset). The red lines represent the regression of the fittest values in each iteration, drawn using generative additive model (gam in R). NormalRefBQ and PrimaryRefBQ are the average base quality of the reads supporting the reference allele in the normal and tumor samples, respectively. PrimaryAltBQ is the average base quality of the reads supporting the alternate allele in the tumor sample. NormalRefAD is the number of reads supporting the reference allele in the normal sample. NormalAltAD is the number of reads supporting the alternate allele in the normal sample. PrimaryRefAD is the number of reads supporting the reference allele in the tumor sample. PrimaryAltAD is the number of reads supporting the alternate allele in the tumor sample. TumorVAF is the variant allele fraction in the tumor sample in percentage.


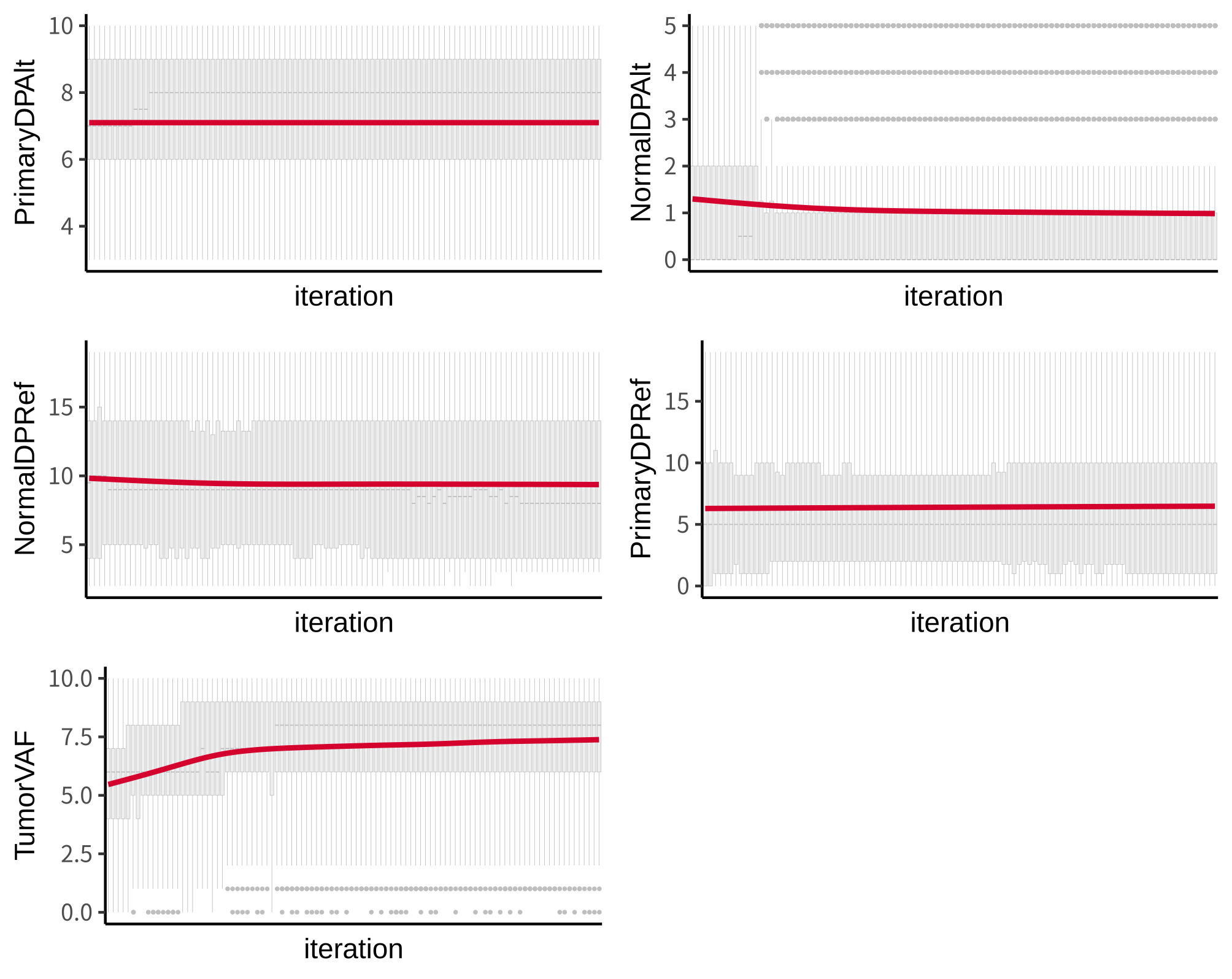


**Fig. S10.** Convergence of filter parameters in the FIREVAT refinement results of the MC3 validation dataset (Varscan callset). The red lines represent the regression of the fittest values in each iteration, drawn using generative additive model (gam in R). PrimaryDPAlt is the number of reads supporting the alternate allele in the tumor sample. NormalDPAlt is the number of reads supporting the alternate allele in the normal sample. NormalDPRef is the number of reads supporting the reference allele in the normal sample. PrimaryDPRef is the number of reads supporting the reference allele in the tumor sample. TumorVAF is the variant allele fraction in the tumor sample in percentage.


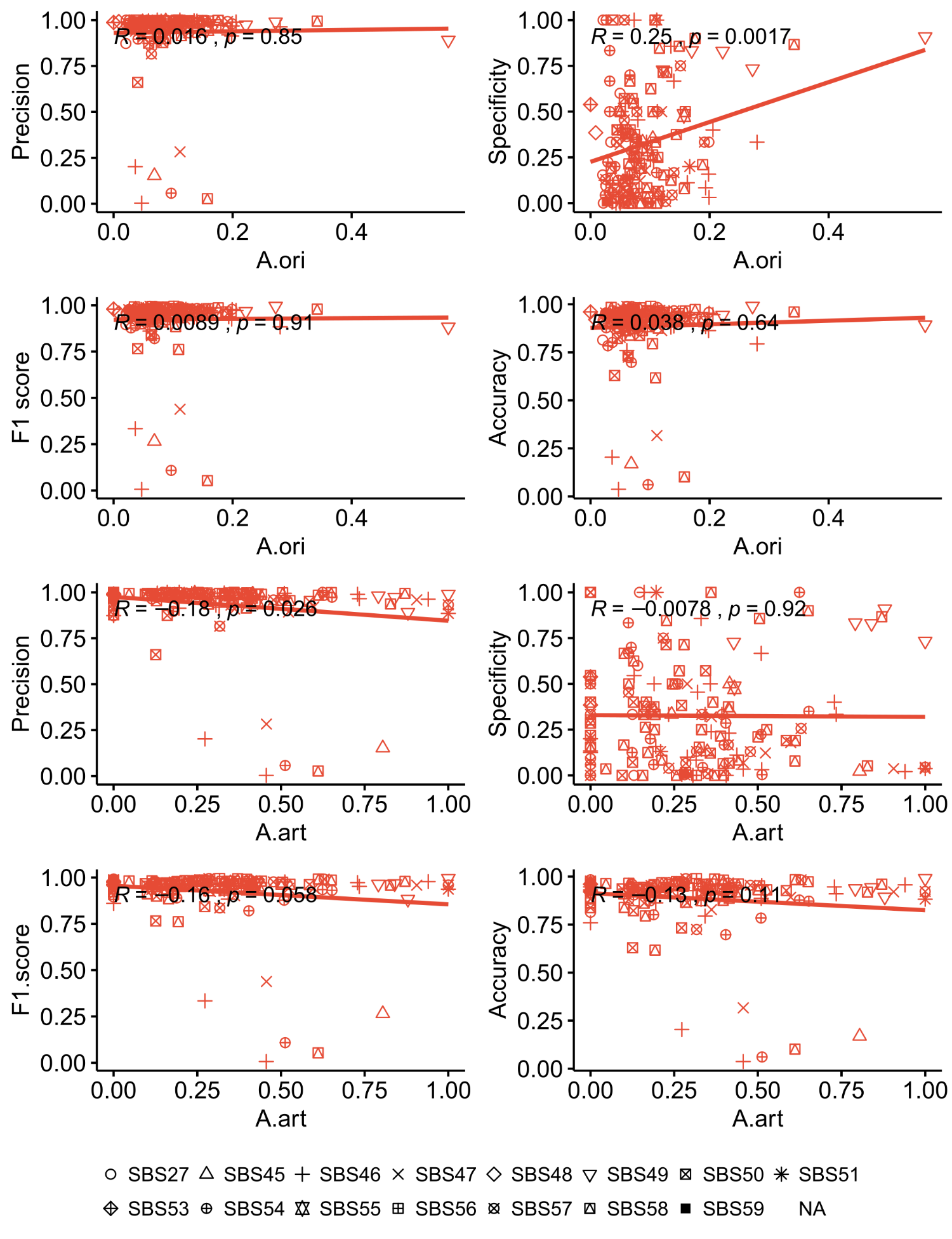


**Fig. S11.** Correlation between FIREVAT performance and artifactual signature weights in the MC3 validation dataset (MuTect callset). A.ori is the sum of artifact signature weights obtained from the signature analysis of unrefined mutations. A.art is the sum of artifact signature weights obtained from the signature analysis of artifactual mutations. In each figure, the artifact signature with the highest weight from each sample is plotted.

**
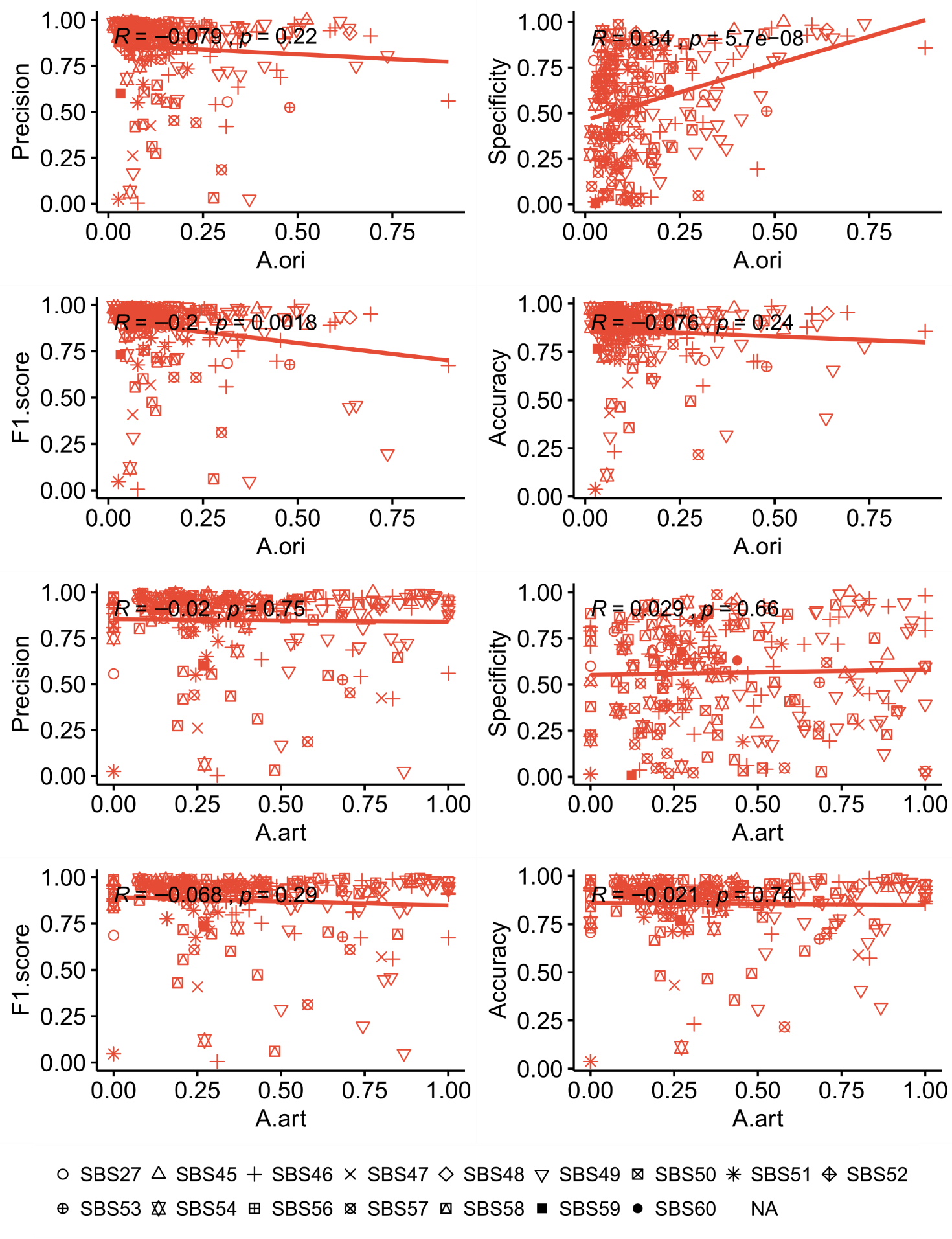
**

**Fig. S12.** Correlation between FIREVAT performance and artifactual signature weights in the MC3 validation dataset (Muse callset)**.** A.ori is the sum of artifact signature weights obtained from the signature analysis of unrefined mutations. A.art is the sum of artifact signature weights obtained from the signature analysis of artifactual mutations. In each figure, the artifact signature with the highest weight from each sample is plotted.


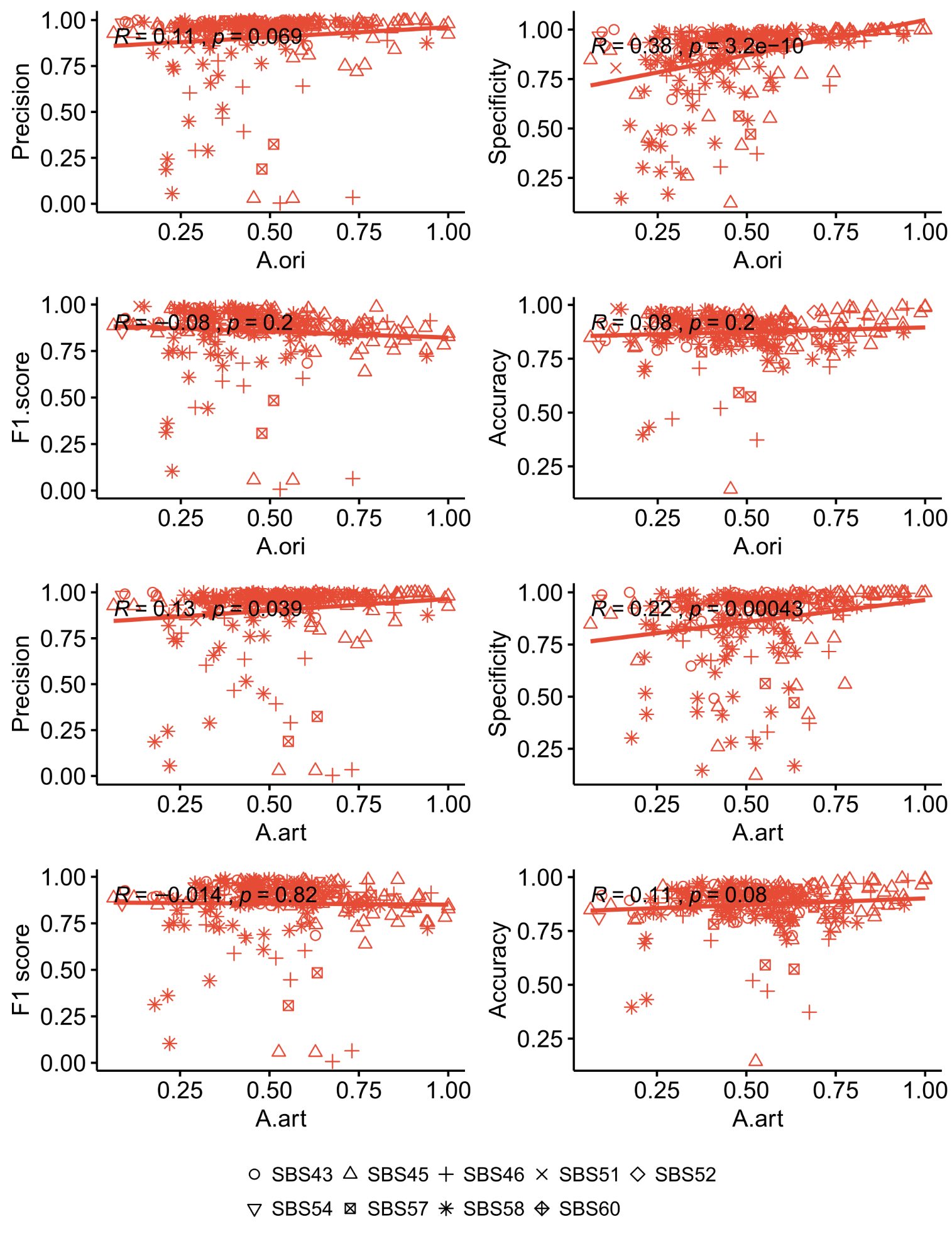


**Fig. S13.** Correlation between FIREVAT performance and artifactual signature weights in the MC3 validation dataset (Varscan callset)**.** A.ori is the sum of artifact signature weights obtained from the signature analysis of unrefined mutations. A.art is the sum of artifact signature weights obtained from the signature analysis of artifactual mutations. In each figure, the artifact signature with the highest weight from each sample is plotted.


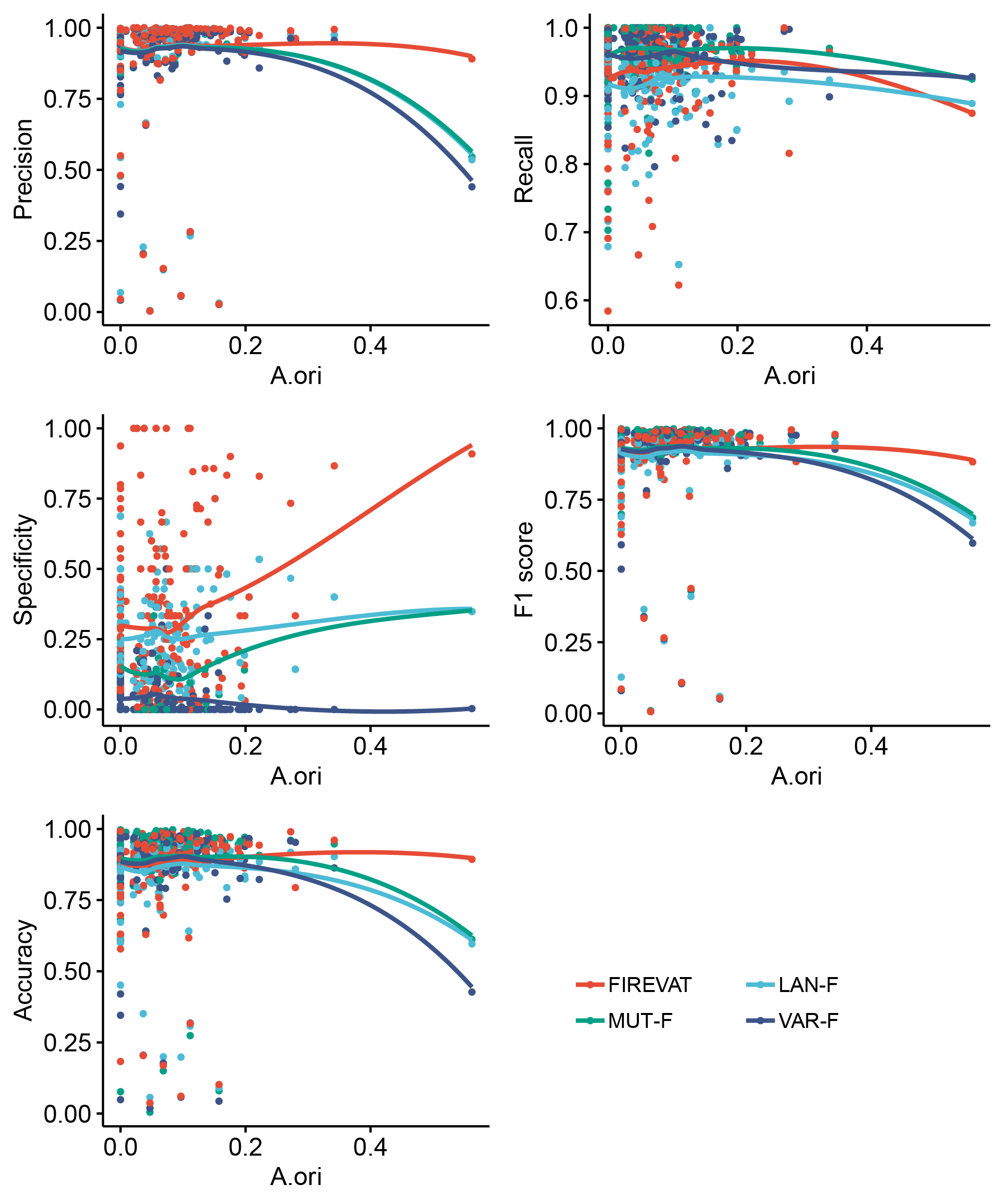


**Fig. S14.** Scatterplots of various performance evaluation metrics on the MC3 validation dataset (MuTect callset) from the FIREVAT refinement results and other post variant-caller filtering methods. A.ori is the sum of artifact signature weights obtained from the signature analysis of unrefined mutations.


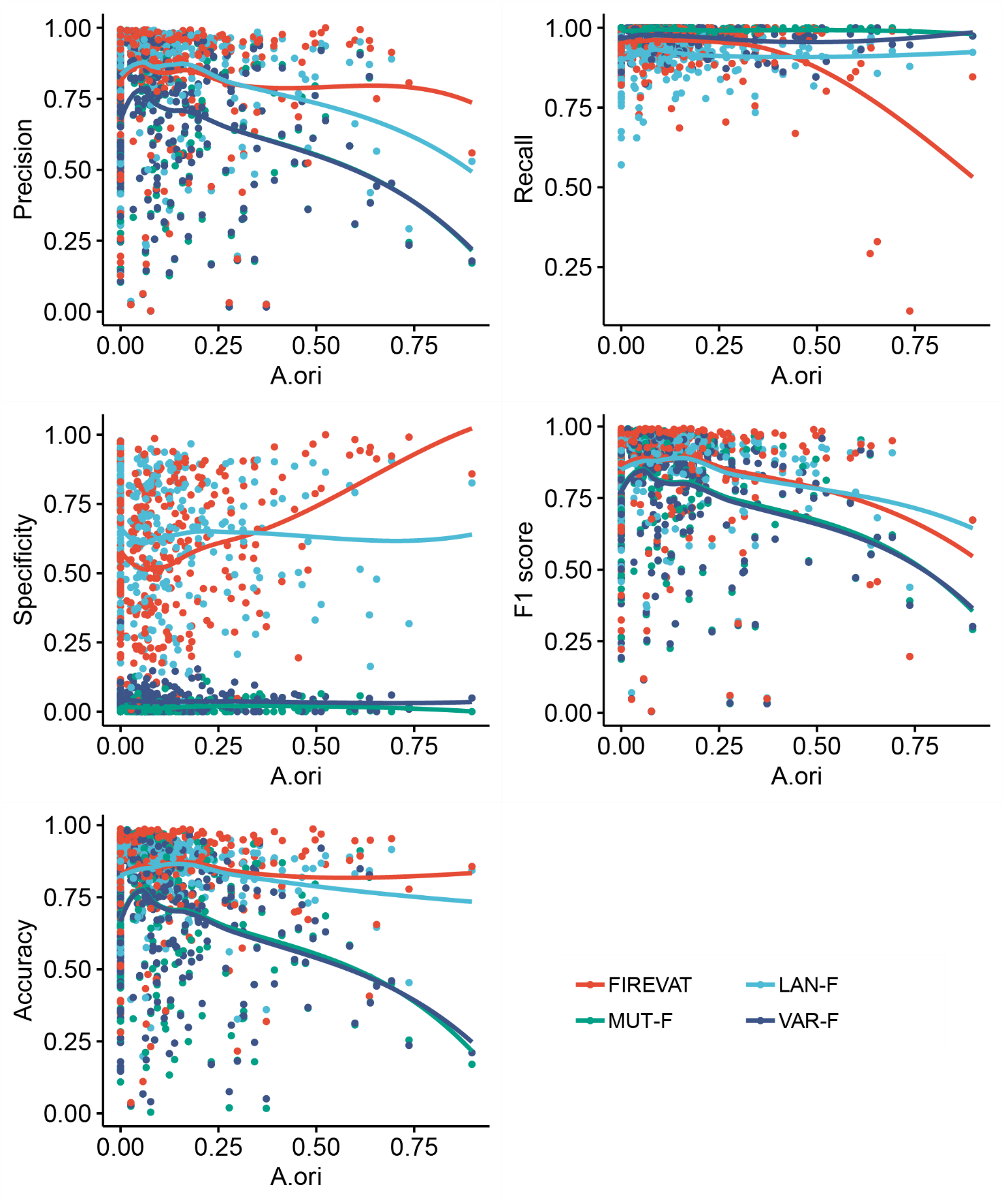


**Fig. S15.** Scatterplots of various performance evaluation metrics on the MC3 validation dataset (Muse callset) from the FIREVAT refinement results and other post variant-caller filtering methods. A.ori is the sum of artifact signature weights obtained from the signature analysis of unrefined mutations.


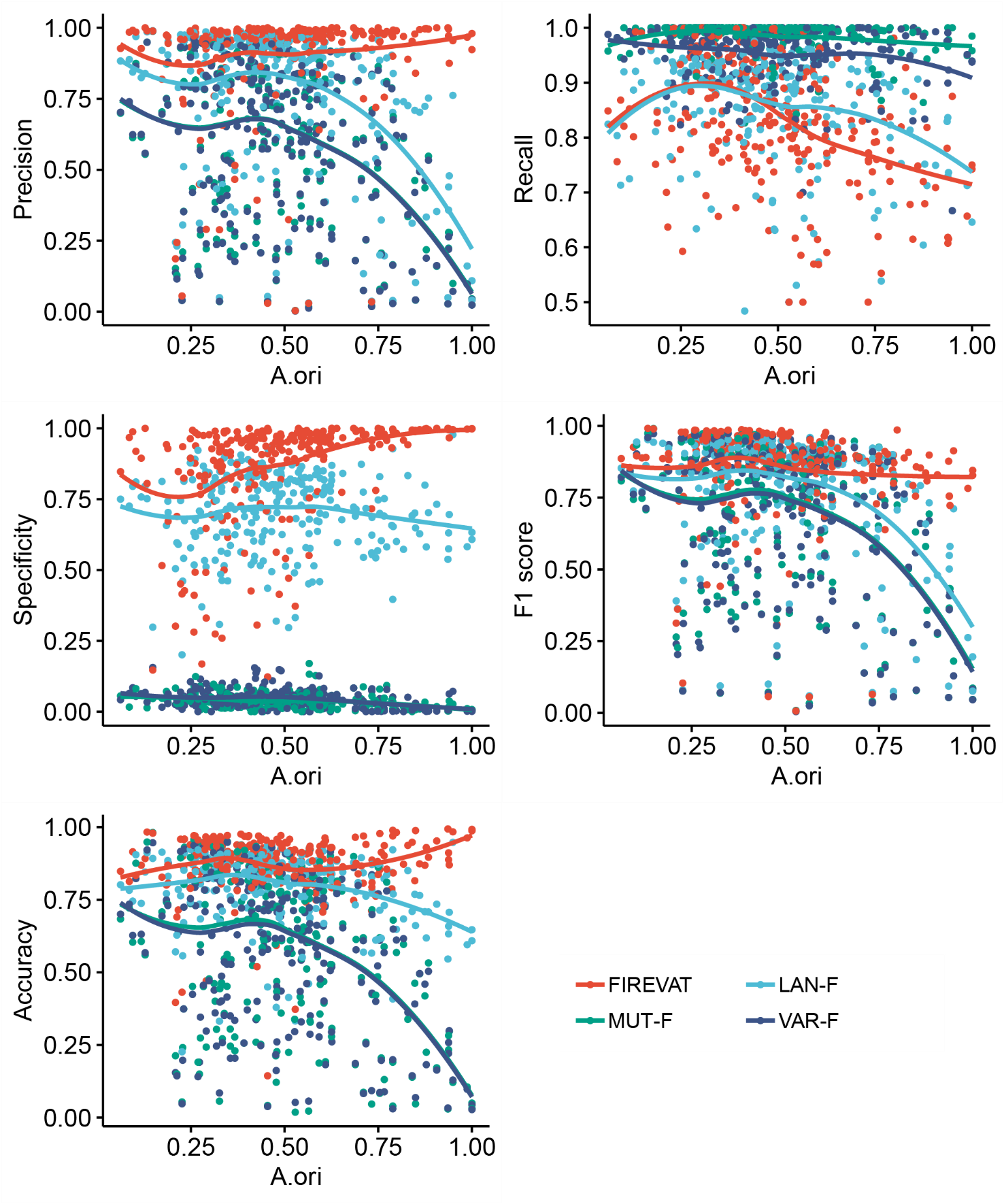


**Fig. S16.** Scatterplots of various performance evaluation metrics on the MC3 validation dataset (Varscan callset) from the FIREVAT refinement results and other post variant-caller filtering methods. A.ori is the sum of artifact signature weights obtained from the signature analysis of unrefined mutations.


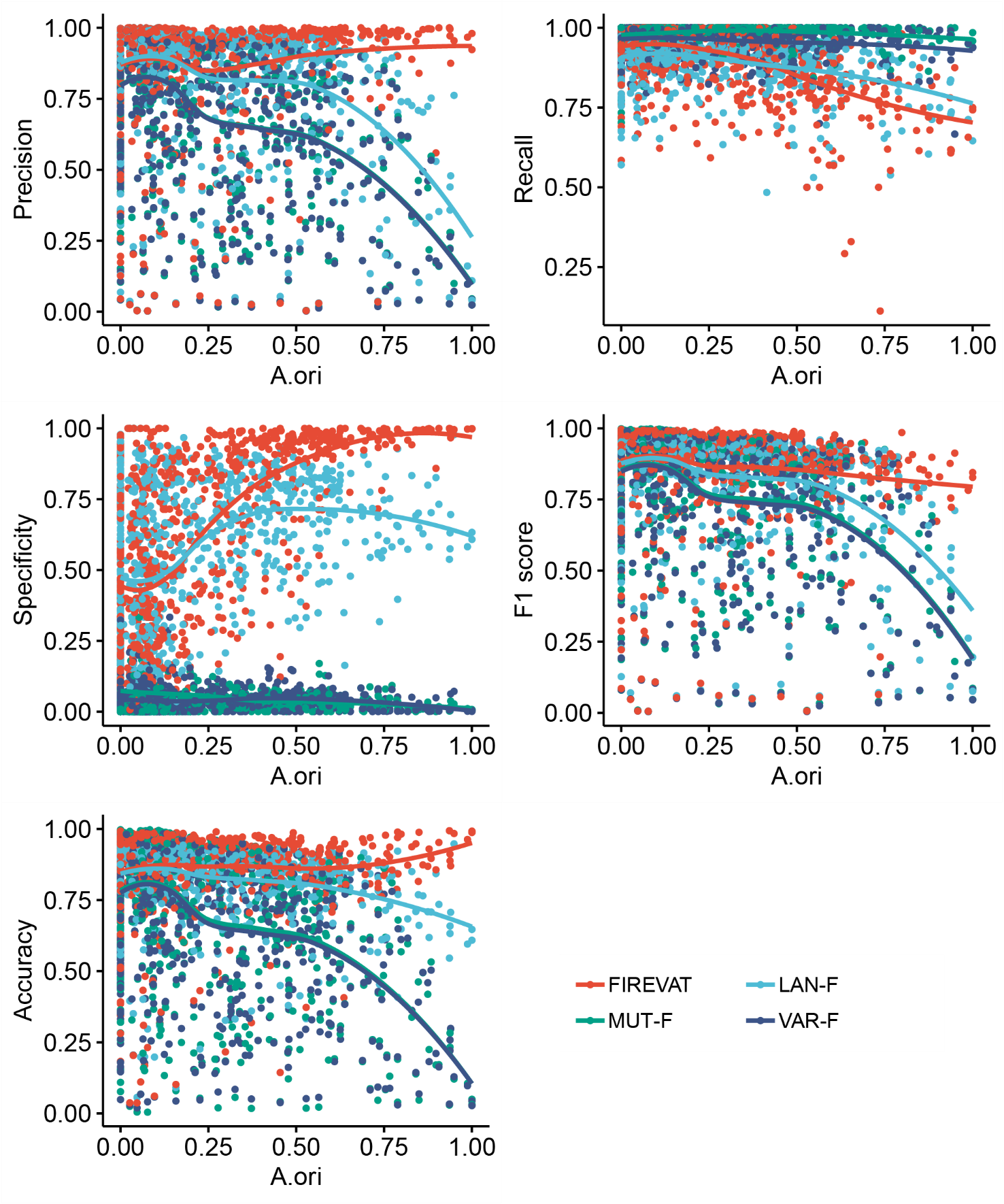


**Fig. S17.** Scatterplots of various performance evaluation metrics on the MC3 validation dataset (all callsets combined) from the FIREVAT refinement results and other post variant-caller filtering methods. A.ori is the sum of artifact signature weights obtained from the signature analysis of unrefined mutations.


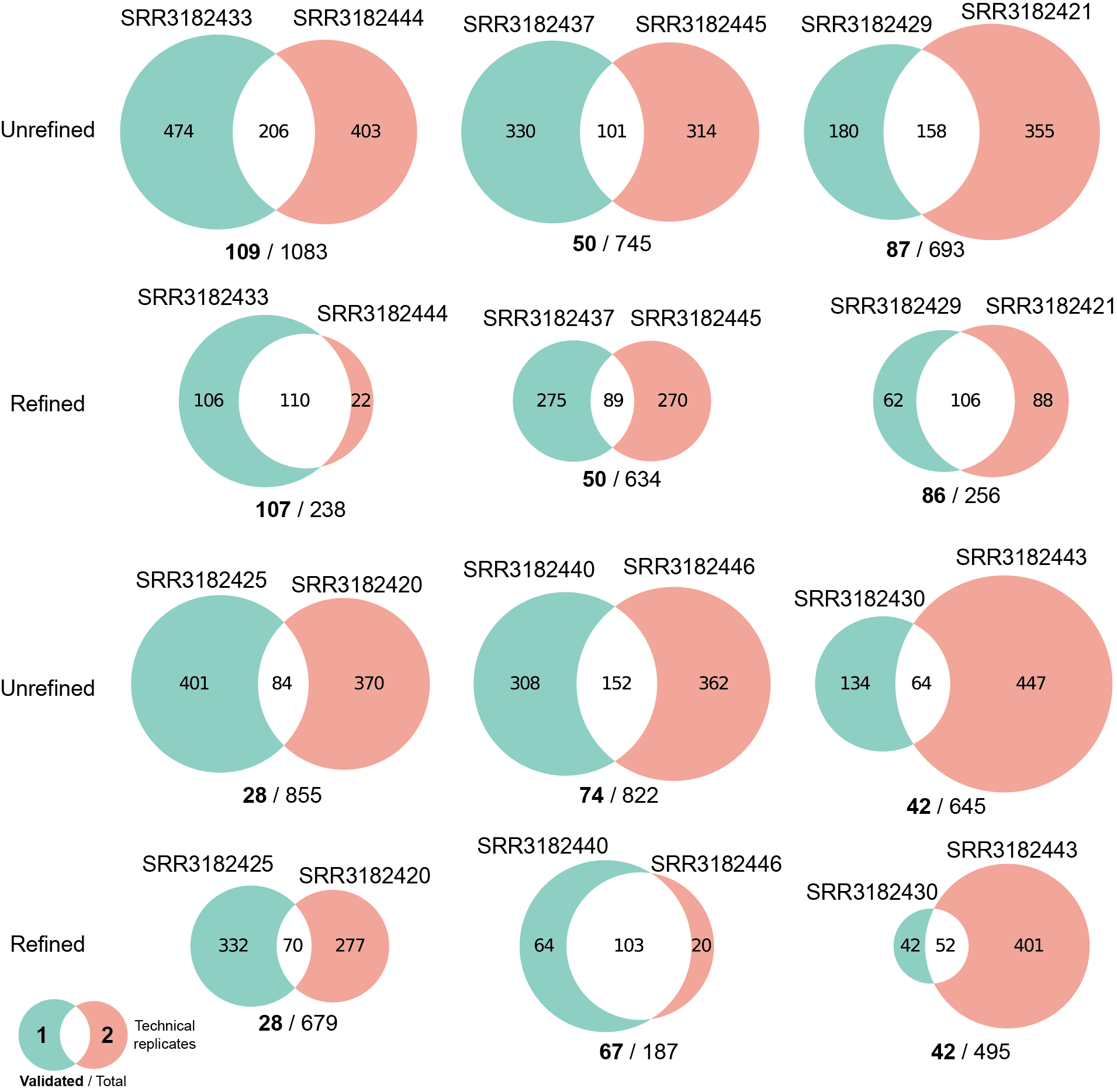


**Fig. S18.** Before (unrefined) and after (refined) FIREVAT refinement on the multi-region whole-exome sequencing data of breast cancer cases (technical replicates).


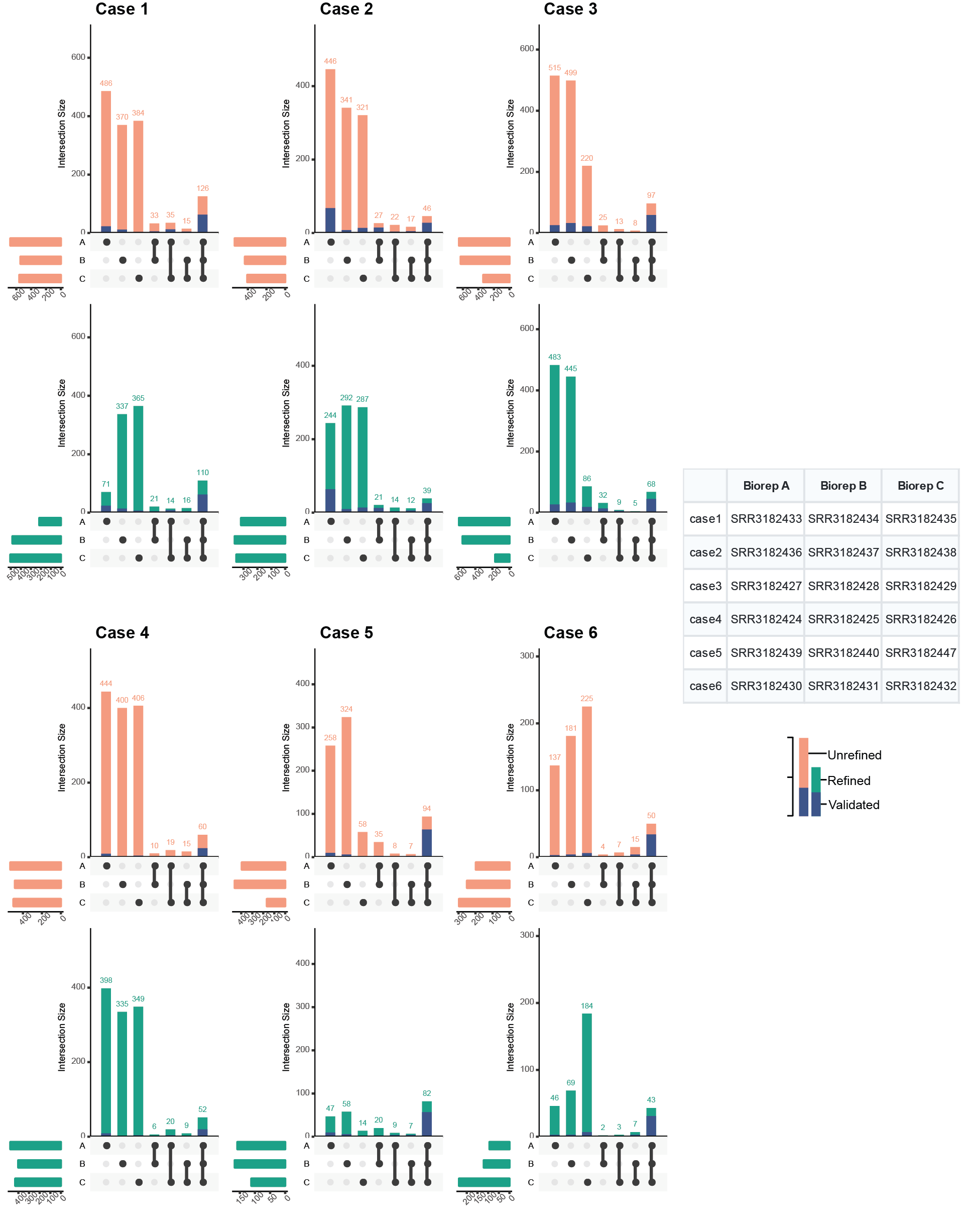


**Fig. S19.** UpSet plots of before (orange) and after (green) FIREVAT refinement on the multi-region whole-exome sequencing data of breast cancer cases (biological replicates).


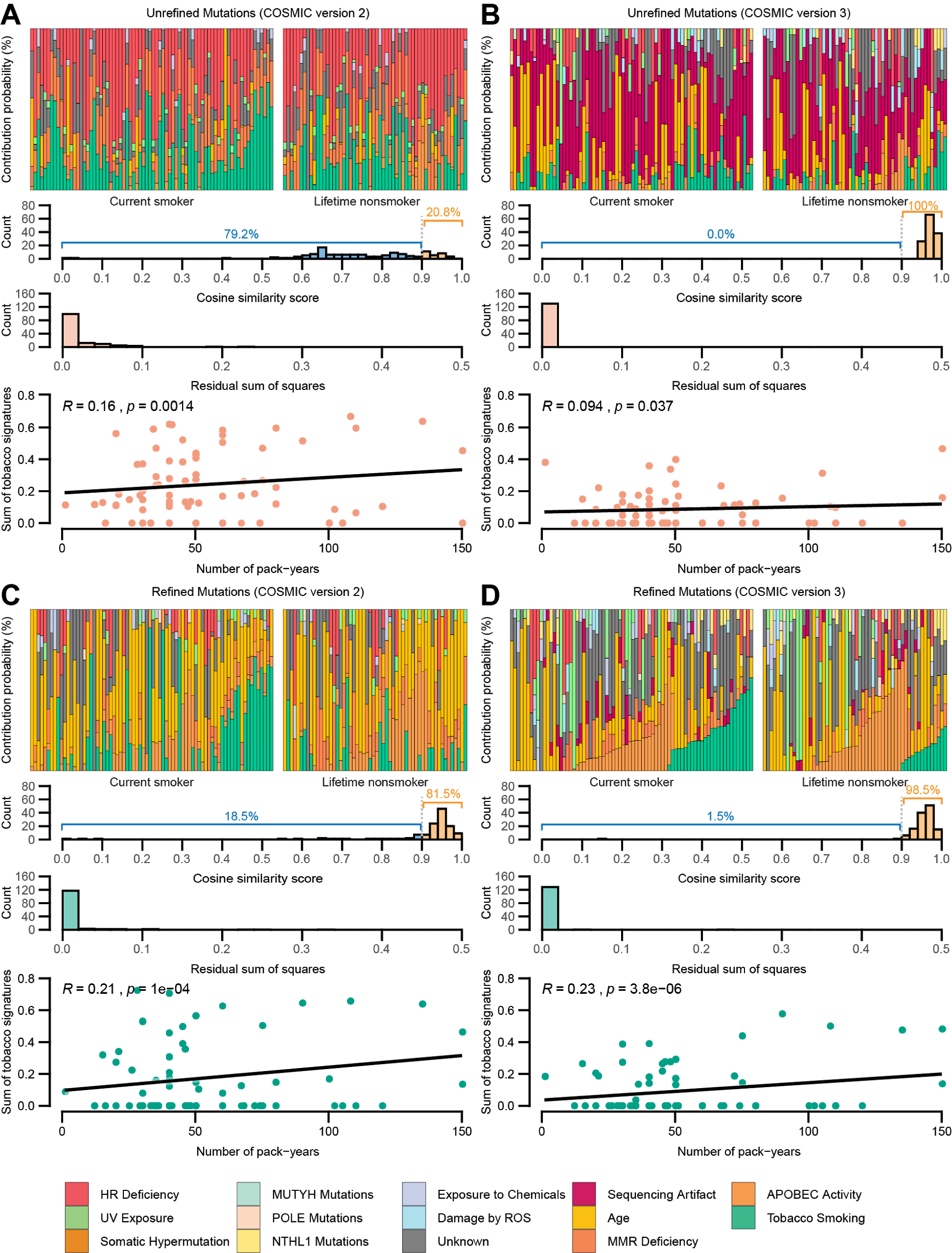


**Fig S20.** Before and after FIREVAT refinement on the TCGA-HNSC dataset using COSMIC mutational signatures versions 2 (30 signatures) and 3 (65 signatures). Of the 130 TCGA-HNSC samples, 103 (79.2%) had a cosine similarity score of less than 0.9 in the analysis of unrefined mutations using the 30 COSMIC signatures. By contrast, all samples had a cosine similarity score of 0.9 or higher using the 65 COSMIC signatures.

**
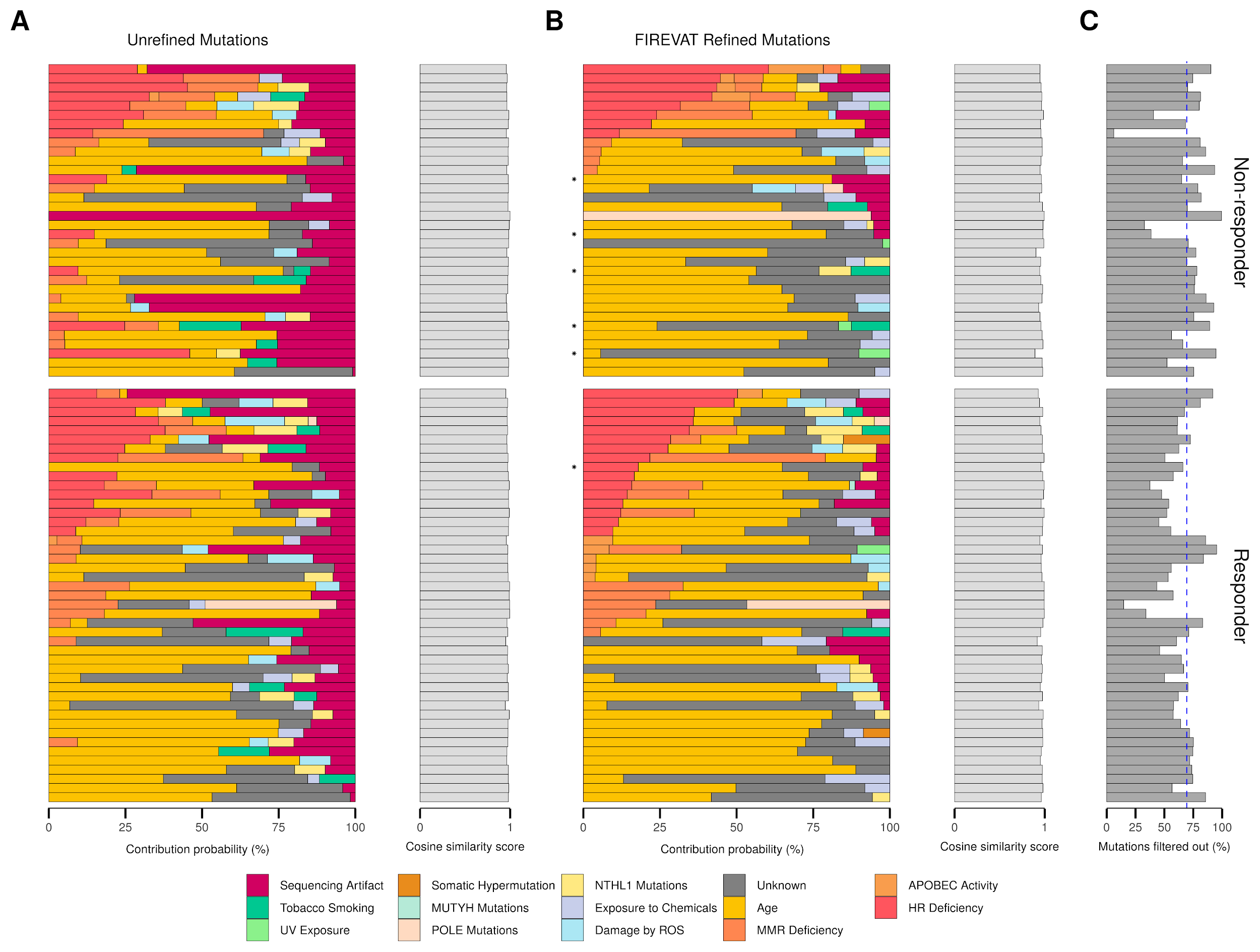
**

**Fig. S21.** The HR deficiency signature SBS3 weight before and after FIREVAT variant refinement in the TCGA platinum therapy responder and non-responder samples (n = 79). The samples shown here include 10 breast cancer (BRCA) samples, 10 pancreatic cancer (PAAD) samples, and 59 stomach cancer (STAD) samples. **(A)** Signature analysis results obtained from unrefined mutations. The first stacked barplot represents the distribution of mutational signature weights (contribution probabilities) using unrefined mutations (before FIREVAT refinement). Samples were grouped by response to therapy. The median sum of artifact signature weights was 15.121%. The second bar plot shows the cosine similarity scores of the samples using the unrefined mutations. The median cosine similarity score was 0.976. **(B)** Signature analysis results obtained from refined mutations. The first stacked barplot represents the distribution of mutational signature weights using FIREVAT refined mutations, grouped by responder and non-responder groups. The median sum of artifactual signature weights was 0%. Samples marked with an asterisk had significant changes in the SBS3 weight before and after FIREVAT refinement. In the non-responder group, all of the samples denoted with asterisk had nonzero SBS3 weight in the analysis of unrefined mutations but had zero SBS3 weight in the analysis of FIREVAT refined mutations. By contrast, in the responder group, the one stomach cancer sample (TCGA-FP-8211) denoted with asterisk had SBS3 weight of zero in the analysis of unrefined mutations but a weight of 17.9% in that of FIREVAT refined mutations. The second bar plot shows the cosine similarity scores of the samples. The median cosine similarity score was 0.970. **(C)** Bar plot of the percentage of mutations filtered out from FIREVAT. The median percentage of filtered out mutations was 69.370% (blue dotted line).


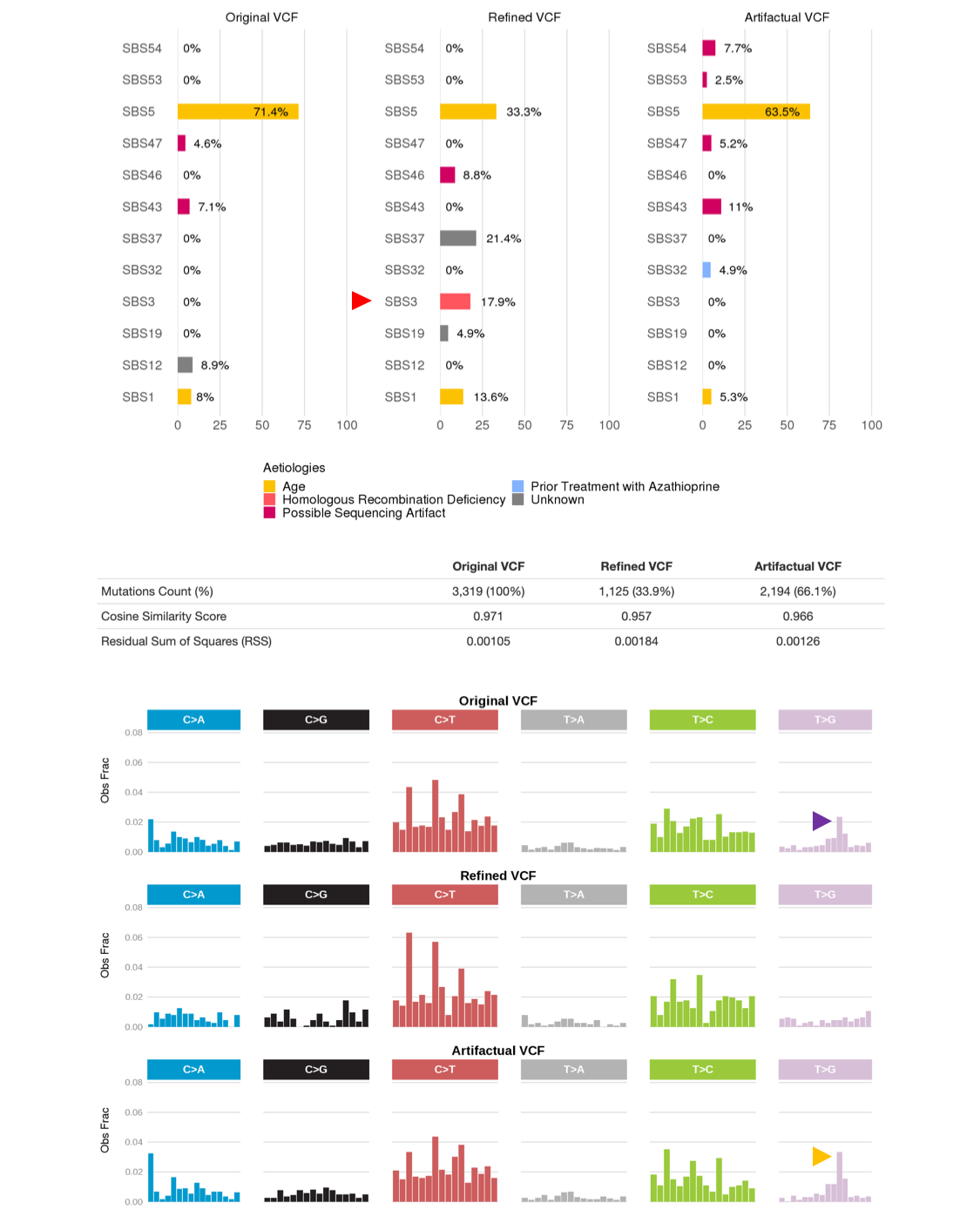


**Fig. S22.** FIREVAT results of the TCGA stomach cancer case (TCGA-FP-8211) with complete clinical response to a regimen of platinum therapy (oxaliplatin). The homologous recombination deficiency signature, SBS3, was not identified using the original unrefined mutations. However, it was detected using the FIREVAT refined mutations with a weight of 17.9% (red arrow). The 96 trinucleotide contexts of the unrefined mutations exhibited a peak (purple arrow) corresponding to the GT>GG peak found in the artifact signature SBS60. These mutations have been filtered out by FIREVAT and are punctuated in the artifactual mutations (yellow arrow).


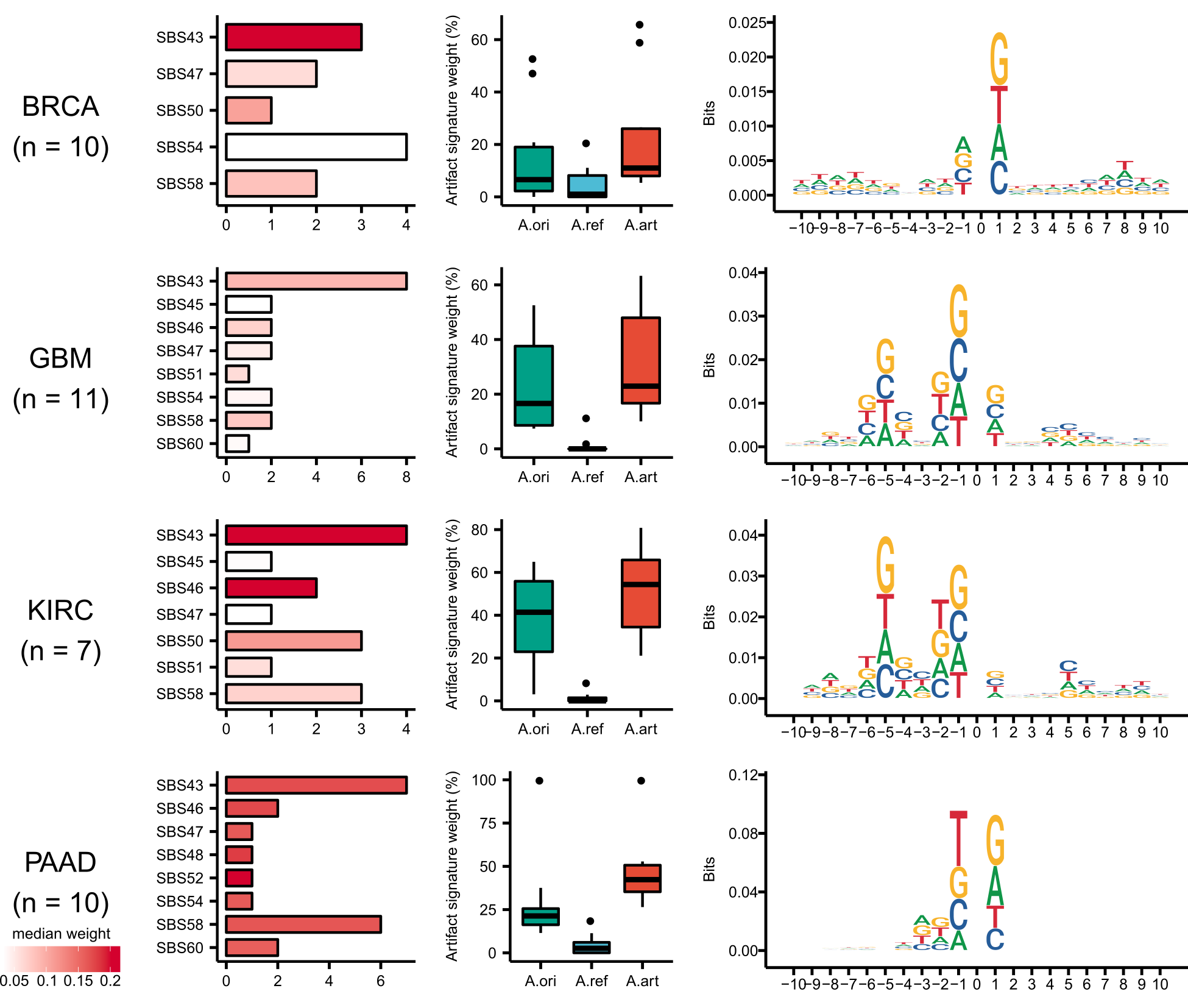


**Fig. S23.** Characteristics of artifactual variants identified by FIREVAT in TCGA-BRCA, TCGA-GBM, TCGA-KIRC, and TCGA-PAAD cohorts. From the left, the first plot for each sample group shows the profiling of the artifact signature occurrences and weights using unrefined mutations. The bar color intensity (white to red) represents the median weight of observed artifactual signature and the bar length represents the number of samples that had the corresponding signature as the most heavily weighted artifactual signature. The second plot shows the distribution of artifactual signatures among the unrefined (green: A.ori), refined (blue: A.ref), and artifactual (orange: A.art) callsets, respectively. The last plot shows the enrichment of sequence motifs in the artifactual variants 10 bases upstream and downstream relative to each mutation position.
