# Supplementary material for: FIREVAT: finding reliable variants without artifacts in human cancer samples using etiologically relevant mutational signatures
Source: Genome Med. 2019 Dec 17;11:81. doi: 10.1186/s13073-019-0695-x (PMC6916105; doi:10.1186/s13073-019-0695-x)
Supplement: Supplementary file 4 — Additional file 4. FIREVAT Report on TCGA-CR-7399. The FIREVAT variant refinement report on the sample TCGA-CR-7399. [file 13073_2019_695_MOESM4_ESM.html]

FIREVAT Report


# **FIREVAT Report**

- **1. Refinement Optimization**
- **2. Optimzed Mutational Signature Identification**
  - **2.1. Identified Signatures**
  - **2.2. Trinucleotide Spectrums**
    - **2.2.1. Observed Spectrum**
    - **2.2.2. Maximum-likelihood Estimaation (MLE) Reconstructed Spectrum**
    - **2.2.3. Residual Spectrum**
  - **2.3. Nucleotide Substitution Types**
- **3. Optimized VCF Statistics**
- **4. Variants with Strand Bias**
  - **4.1. Refined VCF**
  - **4.2. Artifactual VCF**
- **5. VCF Annotation (ClinVar)**
  - **5.1. Refined VCF**
  - **5.2. Artifactual VCF**


---

**Sample ID**

TCGA-CR-7399-01A-11D-2012-08\_TCGA-CR-7399-10A-01D-2013-08\_mutect\_annotated

  

**Sample VCF File**

TCGA-CR-7399-01A-11D-2012-08\_TCGA-CR-7399-10A-01D-2013-08\_mutect\_annotated.vcf

  

**Sample VCF Genome**

hg38

  

**Sample VCF Total Point Mutations**

5,739

  

**FIREVAT Execution Start Datetime**

2019-05-15 22:54:03

  

**FIREVAT Execution End Datetime**

2019-05-16 01:50:47

| FIREVAT Genetic Algorithm (GA) Parameters |  |
| --- | --- |
| GA Population Size | 200 |
| GA Maximum Iteration | 100 |
| GA Run | 100 |
| GA Mutation Probability | 0.100 |

### **1. Refinement Optimization**

| Filter Variable | Filter Direction | Optimized Cutoff |
| --- | --- | --- |
| NormalADRef | >= | 5 |
| NormalADAlt | <= | 4 |
| AvgNormalRefQSS | >= | 18 |
| TumorADRef | >= | 2 |
| AvgTumorRefQSS | >= | 15 |
| TumorADAlt | >= | 5 |
| AvgTumorAltQSS | >= | 14 |
| NormalVAF | <= | 9 |
| TumorVAF | >= | 7 |

| Objective Value | C.refined | W.refined | C.artifact | W.artifact |
| --- | --- | --- | --- | --- |
| 0.817 | 0.979 | 0.0116 | 0.992 | 0.851 |

### **2. Optimzed Mutational Signature Identification**

#### **2.1. Identified Signatures**

---

#### **2.2. Trinucleotide Spectrums**

|  | Original VCF | Refined VCF | Artifactual VCF |
| --- | --- | --- | --- |
| Mutations Count (%) | 5,739 (100%) | 655 (11.41%) | 5,084 (88.59%) |
| Cosine Similarity Score | 0.991 | 0.973 | 0.991 |
| Residual Sum of Squares (RSS) | 0.00151 | 0.00116 | 0.00184 |

---

##### **2.2.1. Observed Spectrum**

---

---

##### **2.2.2. Maximum-likelihood Estimaation (MLE) Reconstructed Spectrum**

---

---

##### **2.2.3. Residual Spectrum**

---

---

#### **2.3. Nucleotide Substitution Types**

### **3. Optimized VCF Statistics**

### **4. Variants with Strand Bias**

#### **4.1. Refined VCF**

```
## None to display.
```

---

#### **4.2. Artifactual VCF**

```
## None to display.
```

### **5. VCF Annotation (ClinVar)**

#### **5.1. Refined VCF**

| CHROM | POS | REF | ALT | GENEINFO | CLNSIG |
| --- | --- | --- | --- | --- | --- |
| chr3 | 179,218,303 | G | A | PIK3CA:5290 | Pathogenic/Likely\_pathogenic |
| chr10 | 94,304,583 | C | T | PLCE1:51196 | Pathogenic |
| chr17 | 7,674,893 | C | A | TP53:7157 | Likely\_pathogenic |

---

#### **5.2. Artifactual VCF**

| CHROM | POS | REF | ALT | GENEINFO | CLNSIG |
| --- | --- | --- | --- | --- | --- |
| chr11 | 68,780,705 | C | A | CPT1A:1374 | Pathogenic |
