# Supplementary material for: FIREVAT: finding reliable variants without artifacts in human cancer samples using etiologically relevant mutational signatures
Source: Genome Med. 2019 Dec 17;11:81. doi: 10.1186/s13073-019-0695-x (PMC6916105; doi:10.1186/s13073-019-0695-x)
Supplement: Supplementary file 5 — Additional file 5. FIREVAT Report on TCGA-44-2662-01B. The FIREVAT variant refinement report on the sample TCGA-44-2662-01B. [file 13073_2019_695_MOESM5_ESM.html]

FIREVAT Report


# **FIREVAT Report**

- **1. Refinement Optimization**
- **2. Optimzed Mutational Signature Identification**
  - **2.1. Identified Signatures**
  - **2.2. Trinucleotide Spectrums**
    - **2.2.1. Observed Spectrum**
    - **2.2.2. Maximum-likelihood Estimaation (MLE) Reconstructed Spectrum**
    - **2.2.3. Residual Spectrum**
  - **2.3. Nucleotide Substitution Types**
- **3. Optimized VCF Statistics**
- **4. Variants with Strand Bias**
  - **4.1. Refined VCF**
  - **4.2. Artifactual VCF**
- **5. VCF Annotation (ClinVar)**
  - **5.1. Refined VCF**
  - **5.2. Artifactual VCF**


---

**Sample ID**

TCGA-44-2662-01B-02D-A271-08\_TCGA-44-2662-10A-01D-A271-08\_mutect\_annotated

  

**Sample VCF File**

TCGA-44-2662-01B-02D-A271-08\_TCGA-44-2662-10A-01D-A271-08\_mutect\_annotated.vcf

  

**Sample VCF Genome**

hg38

  

**Sample VCF Total Point Mutations**

2,592

  

**FIREVAT Execution Start Datetime**

2019-10-15 17:38:52

  

**FIREVAT Execution End Datetime**

2019-10-15 18:56:36

| FIREVAT Genetic Algorithm (GA) Parameters |  |
| --- | --- |
| GA Population Size | 100 |
| GA Maximum Iteration | 20 |
| GA Run | 20 |
| GA Mutation Probability | 0.100 |

### **1. Refinement Optimization**

| Filter Variable | Filter Direction | Optimized Cutoff |
| --- | --- | --- |
| NormalADRef | >= | 1 |
| NormalADAlt | <= | 5 |
| AvgNormalRefQSS | >= | 13 |
| TumorADRef | >= | 1 |
| AvgTumorRefQSS | >= | 31 |
| TumorADAlt | >= | 3 |
| AvgTumorAltQSS | >= | 12 |
| NormalVAF | <= | 10 |
| TumorVAF | >= | 0 |
| TLOD | >= | 4 |

| Objective Value | C.refined | W.refined | C.artifact | W.artifact |
| --- | --- | --- | --- | --- |
| 0.288 | 0.988 | 0.0712 | 0.982 | 0.32 |

### **2. Optimzed Mutational Signature Identification**

#### **2.1. Identified Signatures**

---

#### **2.2. Trinucleotide Spectrums**

|  | Original VCF | Refined VCF | Artifactual VCF |
| --- | --- | --- | --- |
| Mutations Count (%) | 2,592 (100%) | 642 (24.77%) | 1,950 (75.23%) |
| Cosine Similarity Score | 0.982 | 0.984 | 0.97 |
| Residual Sum of Squares (RSS) | 0.000937 | 0.00158 | 0.00177 |

---

##### **2.2.1. Observed Spectrum**

---

---

##### **2.2.2. Maximum-likelihood Estimaation (MLE) Reconstructed Spectrum**

---

---

##### **2.2.3. Residual Spectrum**

---

---

#### **2.3. Nucleotide Substitution Types**

### **3. Optimized VCF Statistics**

### **4. Variants with Strand Bias**

#### **4.1. Refined VCF**

---

#### **4.2. Artifactual VCF**

### **5. VCF Annotation (ClinVar)**

#### **5.1. Refined VCF**

---

#### **5.2. Artifactual VCF**
