# Supplementary material for: FIREVAT: finding reliable variants without artifacts in human cancer samples using etiologically relevant mutational signatures
Source: Genome Med. 2019 Dec 17;11:81. doi: 10.1186/s13073-019-0695-x (PMC6916105; doi:10.1186/s13073-019-0695-x)
Supplement: Supplementary file 6 — Additional file 6. FIREVAT Report on TCGA-EE-A29B. The FIREVAT variant refinement report on the sample TCGA-EE-A29B. [file 13073_2019_695_MOESM6_ESM.html]

FIREVAT Report


# **FIREVAT Report**

- **1. Refinement Optimization**
- **2. Optimzed Mutational Signature Identification**
  - **2.1. Identified Signatures**
  - **2.2. Trinucleotide Spectrums**
    - **2.2.1. Observed Spectrum**
    - **2.2.2. Maximum-likelihood Estimation (MLE) Reconstructed Spectrum**
    - **2.2.3. Residual Spectrum**
  - **2.3. Nucleotide Substitution Types**
- **3. Optimized VCF Statistics**
- **4. Variants with Strand Bias**
  - **4.1. Refined VCF**
  - **4.2. Artifactual VCF**
- **5. VCF Annotation (ClinVar)**
  - **5.1. Refined VCF**
  - **5.2. Artifactual VCF**


---

**Sample ID**

C828.TCGA-EE-A29B-06A-11D-A197-08.2.mutect.tcga\_filtered.reheadered

  

**Sample VCF File**

C828.TCGA-EE-A29B-06A-11D-A197-08.2.mutect.tcga\_filtered.reheadered.vcf

  

**Sample VCF Genome**

hg19

  

**Sample VCF Total Point Mutations**

107,372

  

**FIREVAT Execution Start Datetime**

2019-10-16 05:47:09

  

**FIREVAT Execution End Datetime**

2019-10-16 10:33:15

| FIREVAT Genetic Algorithm (GA) Parameters |  |
| --- | --- |
| GA Population Size | 200 |
| GA Maximum Iteration | 100 |
| GA Run | 100 |
| GA Mutation Probability | 0.100 |

### **1. Refinement Optimization**

| Filter Variable | Filter Direction | Optimized Cutoff |
| --- | --- | --- |
| PrimaryBQ | >= | 16 |
| NormalADRef | >= | 16 |
| NormalADAlt | <= | 1 |
| PrimaryADRef | >= | 16 |
| PrimaryADAlt | >= | 9 |
| TumorVAF | >= | 5 |

| Objective Value | C.refined | W.refined | C.artifact | W.artifact |
| --- | --- | --- | --- | --- |
| 0.297 | 0.998 | 0.0339 | 0.993 | 0.31 |

### **2. Optimzed Mutational Signature Identification**

#### **2.1. Identified Signatures**

---

#### **2.2. Trinucleotide Spectrums**

|  | Original VCF | Refined VCF | Artifactual VCF |
| --- | --- | --- | --- |
| Mutations Count (%) | 107,372 (100%) | 816 (0.76%) | 106,556 (99.24%) |
| Cosine Similarity Score | 0.978 | 0.997 | 0.978 |
| Residual Sum of Squares (RSS) | 0.000819 | 0.000704 | 0.000809 |

---

##### **2.2.1. Observed Spectrum**

---

---

##### **2.2.2. Maximum-likelihood Estimation (MLE) Reconstructed Spectrum**

---

---

##### **2.2.3. Residual Spectrum**

---

---

#### **2.3. Nucleotide Substitution Types**

### **3. Optimized VCF Statistics**

### **4. Variants with Strand Bias**

#### **4.1. Refined VCF**

```
## None to display.
```

---

#### **4.2. Artifactual VCF**

```
## None to display.
```

### **5. VCF Annotation (ClinVar)**

#### **5.1. Refined VCF**

| CHROM | POS | REF | ALT | GENEINFO | CLNSIG |
| --- | --- | --- | --- | --- | --- |
| chr2 | 209,113,113 | G | A | IDH1:3417 | Pathogenic/Likely\_pathogenic |
| chr15 | 35,083,353 | C | T | ACTC1:70|LOC101928174:101928174 | Likely\_pathogenic |

---

#### **5.2. Artifactual VCF**

| CHROM | POS | REF | ALT | GENEINFO | CLNSIG |
| --- | --- | --- | --- | --- | --- |
| chr1 | 151,338,921 | C | A | SELENBP1:8991 | Likely\_pathogenic |
| chr3 | 70,008,546 | A | G | MITF:4286 | Likely\_pathogenic |
| chr3 | 165,548,529 | T | C | BCHE:590 | Pathogenic/Likely\_pathogenic |
| chr5 | 39,342,214 | G | T | C9:735 | Pathogenic/Likely\_pathogenic |
| chr4 | 187,158,034 | G | A | KLKB1:3818 | Pathogenic |
| chr7 | 44,192,981 | G | T | GCK:2645 | Likely\_pathogenic |
| chr7 | 142,458,451 | A | T | PRSS1:5644|TRB:6957 | Pathogenic |
| chr8 | 11,606,312 | T | C | GATA4:2626 | Pathogenic |
| chr8 | 11,615,695 | A | G | GATA4:2626 | Pathogenic |
| chr11 | 18,290,859 | C | T | SAA1:6288 | Pathogenic |
| chr11 | 46,761,055 | G | A | F2:2147 | Pathogenic |
| chr11 | 111,635,566 | C | T | PPP2R1B:5519 | Pathogenic |
| chr10 | 70,645,376 | A | C | STOX1:219736 | Pathogenic |
| chr12 | 25,380,276 | T | C | KRAS:3845 | Pathogenic |
| chr15 | 43,552,349 | C | A | TGM5:9333 | Pathogenic |
| chr14 | 73,685,900 | C | A | PSEN1:5663 | Pathogenic |
| chr14 | 74,060,517 | A | G | ACOT4:122970 | Likely\_pathogenic |
| chr16 | 16,244,584 | C | T | ABCC6:368 | Pathogenic |
| chrM | 14,766 | C | T | MT-CYB:4519 | Likely\_pathogenic |
