# Supplementary material for: FIREVAT: finding reliable variants without artifacts in human cancer samples using etiologically relevant mutational signatures
Source: Genome Med. 2019 Dec 17;11:81. doi: 10.1186/s13073-019-0695-x (PMC6916105; doi:10.1186/s13073-019-0695-x)
Supplement: Supplementary file 7 — Additional file 7. FIREVAT Report on HCC1954. The FIREVAT variant refinement report on the sample HCC1954. [file 13073_2019_695_MOESM7_ESM.html]

FIREVAT Report


# **FIREVAT Report**

- **1. Refinement Optimization**
- **2. Optimzed Mutational Signature Identification**
  - **2.1. Identified Signatures**
  - **2.2. Trinucleotide Spectrums**
    - **2.2.1. Observed Spectrum**
    - **2.2.2. Maximum-likelihood Estimation (MLE) Reconstructed Spectrum**
    - **2.2.3. Residual Spectrum**
  - **2.3. Nucleotide Substitution Types**
- **3. Optimized VCF Statistics**
- **4. Variants with Strand Bias**
  - **4.1. Refined VCF**
  - **4.2. Artifactual VCF**
- **5. VCF Annotation (ClinVar)**
  - **5.1. Refined VCF**
  - **5.2. Artifactual VCF**


---

**Sample ID**

DCC\_PCAWG\_Cell\_Lines\_HCC1954

  

**Sample VCF File**

DCC\_PCAWG\_Cell\_Lines\_HCC1954.vcf

  

**Sample VCF Genome**

hg19

  

**Sample VCF Total Point Mutations**

62,176

  

**FIREVAT Execution Start Datetime**

2019-10-22 09:05:03

  

**FIREVAT Execution End Datetime**

2019-10-22 12:53:59

| FIREVAT Genetic Algorithm (GA) Parameters |  |
| --- | --- |
| GA Population Size | 100 |
| GA Maximum Iteration | 20 |
| GA Run | 20 |
| GA Mutation Probability | 0.100 |

### **1. Refinement Optimization**

| Filter Variable | Filter Direction | Optimized Cutoff |
| --- | --- | --- |
| ControlVAF | <= | 1 |
| TumorVAF | >= | 4 |
| MappingQuality | >= | 35 |
| ControlDPRef | >= | 5 |
| ControlDPAlt | <= | 3 |
| TumorDPRef | >= | 0 |
| TumorDPAlt | >= | 6 |

| Objective Value | C.refined | W.refined | C.artifact | W.artifact |
| --- | --- | --- | --- | --- |
| 0.806 | 0.995 | 0.116 | 0.978 | 0.938 |

### **2. Optimzed Mutational Signature Identification**

#### **2.1. Identified Signatures**

---

#### **2.2. Trinucleotide Spectrums**

|  | Original VCF | Refined VCF | Artifactual VCF |
| --- | --- | --- | --- |
| Mutations Count (%) | 62,176 (100%) | 19,425 (31.24%) | 42,751 (68.76%) |
| Cosine Similarity Score | 0.978 | 0.94 | 0.978 |
| Residual Sum of Squares (RSS) | 0.00626 | 0.00201 | 0.0127 |

---

##### **2.2.1. Observed Spectrum**

---

---

##### **2.2.2. Maximum-likelihood Estimation (MLE) Reconstructed Spectrum**

---

---

##### **2.2.3. Residual Spectrum**

---

---

#### **2.3. Nucleotide Substitution Types**

### **3. Optimized VCF Statistics**

### **4. Variants with Strand Bias**

#### **4.1. Refined VCF**

---

#### **4.2. Artifactual VCF**

### **5. VCF Annotation (ClinVar)**

#### **5.1. Refined VCF**

---

#### **5.2. Artifactual VCF**
